# Use of fluoroquinolones and risk of rhegmatogenous retinal detachment: a retrospective cohort study using two nationwide representative claims databases

**DOI:** 10.3389/fphar.2024.1414221

**Published:** 2024-12-11

**Authors:** Ting-Yu Lin, Jiun-Ling Wang, Grace Hsin-Min Wang, Yu-Yun Huang, Ming-Ching Chen, Yaa-Hui Dong, Wei-Hsuan Lo-Ciganic

**Affiliations:** ^1^ Department of Pharmacy, College of Pharmaceutical Sciences, National Yang Ming Chiao Tung University, Taipei, Taiwan; ^2^ Department of Pharmacy Administration, Chang Gung Medical Foundation, Taoyuan City, Taiwan; ^3^ Department of Internal Medicine, National Cheng Kung University Hospital, Tainan, Taiwan; ^4^ Department of Medicine, College of Medicine, National Cheng Kung University, Tainan, Taiwan; ^5^ Department of Pharmaceutical Outcomes and Policy, College of Pharmacy, University of Florida, Gainesville, FL, United States; ^6^ Department of Ophthalmology, Taipei Veterans General Hospital, Taipei, Taiwan; ^7^ Institute of Public Health, School of Medicine, National Yang Ming Chiao Tung University, Taipei, Taiwan; ^8^ Division of General Internal Medicine, Department of Medicine, School of Medicine, University of Pittsburgh, Pittsburgh, PA, United States; ^9^ Center for Pharmaceutical Policy and Prescribing, University of Pittsburgh, Pittsburgh, PA, United States; ^10^ Center for Clinical Artificial Intelligence, University of Pittsburgh, Pittsburgh, United States; ^11^ North Florida/South Georgia Veterans Health System Geriatric Research Education and Clinical Center, Gainesville, FL, United States

**Keywords:** fluoroquinolones, rhegmatogenous retinal detachment, cohort study, pharmacoepidemiology, real-world data

## Abstract

**Background:**

Although biological plausibility suggests that fluoroquinolones could lead to rhegmatogenous retinal detachment (RRD) through collagen degradation, real-world evidence on their relative risk of RRD is inconsistent, with limited information on absolute risk estimates.

**Objective:**

The study aimed to estimate the RRD risk associated with fluoroquinolones versus other antibiotics with similar indications (i.e., comparison antibiotics).

**Methods:**

We conducted a retrospective cohort study analyzing claims data from adult patients who initiated fluoroquinolones or amoxicillin/clavulanate or ampicillin/sulbactam or extended-spectrum cephalosporins using the Taiwan National Health Insurance Research Database (2009–2018) and the United States IBM MarketScan Database (2011–2020). Patients were followed for up to 90 days after cohort entry. For each country’s data, after 1:1 propensity score (PS) matching, we used Cox regression models to estimate RRD risks, presented with hazard ratios (HR) with 95% confidence interval (95% CI). We used random-effects meta-analyses to derive pooled HRs across both counties.

**Results:**

Of 24,172,032 eligible patients comprising 7,944,620 insured Taiwanese (mean age [SD], 46 [18] years; 45% male) and 16,227,412 United States commercially insured individuals (mean age [SD], 47 [16] years; 40% male), 10,137,468 patients initiated fluoroquinolones, 10,203,794 initiated amoxicillin/clavulanate or ampicillin/sulbactam, and 3,830,770 initiated extended-spectrum cephalosporins. After PS matching, similar RRD incidence rates were observed between fluoroquinolones and amoxicillin/clavulanate or ampicillin/sulbactam users (0.33 [95% CI, 0.19–0.56] versus 0.35 [95% CI, 0.26–0.46] per 1,000 person-years), yielding an HR of 0.97 (95% CI, 0.76–1.23). The RRD incidence rates were also similar comparing fluoroquinolones to extended-spectrum cephalosporins (0.36 [95% CI, 0.22–0.57] versus 0.34 [95% CI, 0.22–0.50] per 1,000 person-years; HR, 1.08 [95% CI, 0.92–1.27]). The comparative safety profiles remained consistent by country, various patient characteristic (e.g., diabetes or ophthalmic conditions), type of fluoroquinolones, follow-up duration, or treatment setting.

**Conclusion:**

This large-scale study, leveraging real-world data from Taiwan and the United States, showed a low and comparable RRD risk among adults who initiated fluoroquinolones or other antibiotics with similar indications. This suggests that the RRD risk should not deter the use of fluoroquinolone when clinically indicated.

## Introduction

Fluoroquinolones are antibiotics known for their effective tissue penetration; they are widely prescribed for various infections, including lower respiratory tract infections and genitourinary tract infections (Metlay et al., 2019; Kalil et al., 2016; Gupta et al., 2011; Chou et al., 2019; Taiwan Urological Association, 2022; Choe et al., 2018; Van Boeckel et al., 2014). Rhegmatogenous retinal detachment (RRD), the predominant type of retinal detachment (RD), involves a retinal break which allows vitreous fluid to enter the subretinal space, leading to the separation of the neurosensory retina from the retinal pigment epithelium (UpToDate^®^, 2023). The RRD incidence rate is low, ranging from 0.098 to 0.26 per 1,000 person-years (Park et al., 2021; Chen et al., 2016; Achour et al., 2022; van Leeuwen et al., 2021). Nevertheless, without appropriate management, it may lead to significant visual impairment. The known risk factors for RRD include older age, male gender, family history, ocular trauma, intra-ocular surgery, and existing ophthalmic disorders (UpToDate^®^, 2023; Sodhi et al., 2008; Mitry et al., 2010a).

There have been mixed reports about the association between oral fluoroquinolone use and increased RD risk (mainly RRD) (Chui et al., 2014; Raguideau et al., 2016; Shin et al., 2018; Baek et al., 2018; Londhe et al., 2022; Etminan et al., 2012; Fife et al., 2014; Choi et al., 2018; Taher et al., 2022; Pasternak et al., 2013; Kuo et al., 2014; Eftekhari et al., 2014; Kapoor et al., 2014; Daneman et al., 2015), possibly through degrading collagen in the ocular structures (Ghazi and Green, 2002; Ponsioen et al., 2008; Mitry et al., 2010b). However, the results of these real-world studies using various designs were inconsistent. Notably, most studies comparing use versus non-use of fluoroquinolones observed a 1.3- to 4.5-fold higher risk (Chui et al., 2014; Raguideau et al., 2016; Shin et al., 2018; Baek et al., 2018; Londhe et al., 2022; Etminan et al., 2012; Fife et al., 2014; Pasternak et al., 2013; Daneman et al., 2015), while a couple of studies comparing the use of fluoroquinolones versus other comparable antibiotics suggested a null association (Eftekhari et al., 2014; Kapoor et al., 2014). These raise methodological issues about potential residual confounding when utilizing a non-user comparison design. Moreover, the clinical challenge for physicians is not the use or non-use of fluoroquinolones but which alternative antibiotics should be prescribed if any concerns about adverse drug reaction exist. However, little information exists on the RRD incidence rates associated with fluoroquinolones and other antibiotics with similar indications (Pasternak et al., 2013; Eftekhari et al., 2014; Daneman et al., 2015).

There has been an increasing use of multi-databases from different healthcare systems or countries to elucidate drug safety issues because this facilitates the identification of rare adverse outcomes and improves the generalizability of findings (Dong et al., 2018; Pradhan et al., 2022; Maro and Toh, 2022). This retrospective cohort study aims to estimate the RRD incidence rates associated with fluoroquinolones and other antibiotics with similar indications using two nationwide representative claims databases from Taiwan and the United States.

## Materials and methods

### Data sources

We identified eligible patients from (1) the Taiwan National Health Insurance Research Database (NHIRD), which comprises the data of approximately 23 million residents enrolled in a single-payer national health insurance system (National Health Insurance Administration, 2023; Lin et al., 2018) and (2) the United States IBM MarketScan Database that comprises data from more than 200 million beneficiaries enrolled in employer-sponsored healthcare programs since 1995 (Butler et al., 2021). See the online data supplement for detailed descriptions of data sources.

### Application of a common protocol

We used a common protocol to conduct this study for both databases. Given the inherent variations in healthcare systems and data structures across countries, certain database-specific modifications were required (Supplementary Table S1). For example, both the Taiwan NHIRD and United States IBM MarketScan Database contain pharmacy dispensing claims from outpatient visits. However, only the Taiwan NHIRD provides information on drug use at emergency department (ED) visits and during hospitalization, including oral and injectable study antibiotics. Therefore, steps involving medication use in outside outpatient settings or injectable medication use were only applied to the Taiwanese data.

### Study populations and exposure

From each database, we identified patients aged ≥18 years who initiated oral fluoroquinolones, amoxicillin/clavulanate or ampicillin/sulbactam, or extended-spectrum cephalosporins (second-, third-, or fourth-generation cephalosporins) at outpatient, ED, or inpatient visits in corresponding cohort identification periods (Taiwan NHIRD, 2009/1/1–2018/8/31; United States IBM MarketScan, 2011/1/1–2020/9/30). The cohort entry date was defined as the date of the first dispensing of a study antibiotic (codes in Supplementary Table S2). We determined amoxicillin/clavulanate or ampicillin/sulbactam and extended-spectrum cephalosporins as comparison antibiotics, given that their indications are generally similar to those of fluoroquinolones according to the treatment guidelines in Taiwan, other Asian regions, and the United States (Metlay et al., 2019; Kalil et al., 2016; Gupta et al., 2011; Chou et al., 2019; Taiwan Urological Association, 2022; Choe et al., 2018).

We applied several exclusion criteria (for details, see the online data supplement and Supplementary Table S3). Patients with more severe infections are likely to receive injectable treatment, so we excluded patients initiating oral and injectable study antibiotics simultaneously at cohort entry to mitigate confounding by infection severity. We also excluded patients with any history of RD or retinal defects or ever having received RD-related clinical management (scleral buckling, cryotherapy, laser therapy, pneumatic retinopexy, and vitrectomy mainly) to prevent confounding by disease history. Notably, patients who were not at risk of RRD, including those who were blind or receiving evisceration or enucleation of eyeball, were also excluded from the analysis.

### Follow-up and the study outcome

We followed up patients from cohort entry to the first occurrence of RRD, death, disenrollment, or the study’s end within 90 days of treatment initiation (Taiwan NHIRD, 2018/8/31; U.S. IBM MarketScan, 2020/9/30). Previous cohort studies examining fluoroquinolones-related RRD had a wide range of follow-up durations, including 30 (Daneman et al., 2015), 90 (Kuo et al., 2014), 180 (Pasternak et al., 2013), or 365 days (Eftekhari et al., 2014; Kapoor et al., 2014). To ensure a sufficient number of events and minimize potential exposure misclassification, our main analysis truncated the follow-up at 90 days post-cohort entry. We also conducted additional sensitivity analyses to explore variations in follow-up duration.

Due to the absence of validated claims-based codes for RRD, our study team—comprising experts in ophthalmology, pharmacy, and epidemiology—conducted a thorough review of existing observational studies and adopted clinically relevant codes for our analysis. We defined RRD as the first occurrence of any relevant International Classification of Diseases, 9^th^ or 10^th^ Revision, Clinical Modification (ICD-9-CM or ICD-10-CM) codes recorded in any diagnosis position during an outpatient, ED, or inpatient visit within 90 days of cohort entry. To enhance the outcome validity, we only considered cases with RD-related clinical management 14 days before or after the diagnosis date (Supplementary Table S4).

### Covariates

We measured ≥80 potential confounders, including age at cohort entry, sex, acute infection diagnoses (which may serve as indications for study antibiotics), individual comorbidities, Deyo version of Charlson comorbidity index (Deyo et al., 1992; Sundararajan et al., 2004), adapted Diabetes Complications Severity Index (Chang HY. et al., 2012; Glasheen et al., 2017), medications, and resource utilization that may be associated with both study antibiotic use and RRD risk. Our analysis differs from previous studies (Chui et al., 2014; Raguideau et al., 2016; Shin et al., 2018; Baek et al., 2018; Londhe et al., 2022; Etminan et al., 2012; Fife et al., 2014; Choi et al., 2018; Taher et al., 2022; Pasternak et al., 2013; Kuo et al., 2014; Eftekhari et al., 2014; Kapoor et al., 2014; Daneman et al., 2015) by including a more comprehensive list of ophthalmic disorders (e.g., endophthalmitis, myopia, glaucoma, cataract, diabetic retinopathy, disorders of vitreous body, severe eye trauma, intravitreal injection, and intraocular surgery), ophthalmic medications (e.g., anti-bacterial, anti-viral, and corticosteroid eye drops), and resource utilization specific to ophthalmic diseases. These characteristics were identified using diagnosis files or pharmacy dispensing records derived from outpatient, ED, or inpatient claims within 7 days before cohort entry or at cohort entry (acute infection diagnoses) or within 180 days before cohort entry or at cohort entry (comorbidities, other medication use, and resource utilization). Supplementary Tables S5 and S6 provide detailed covariate information.

### Statistical analysis

Using the above covariates, we estimated baseline propensity scores (PS) with multivariable logistic regression models to predict the probability of initiating fluoroquinolones versus comparison antibiotics (either amoxicillin/clavulanate or ampicillin/sulbactam or extended-spectrum cephalosporins). To balance baseline characteristics between study antibiotic groups, we applied a 1:1 PS-matched design for identifying comparison antibiotic initiators, with a nearest-neighbor algorithm without replacement and with a maximum matching caliper 0.025 times the standard deviation (SD) of the logits of PS (SAS Institute Inc, 2023). To assess whether the covariate distributions were balanced after PS matching, we computed absolute standardized mean differences (aSMD) for each covariate, with a value < 0.1 indicating balance between study antibiotic groups (Austin, 2011).

We applied a Poisson distribution to estimate the incidence rate and the 95% confidence interval (CI) of RRD for each study antibiotic group. We used Cox proportional hazard models to estimate the hazard ratio (HR) with 95% CI of RRD comparing fluoroquinolone versus comparison antibiotics. All analyses were conducted by comparison antibiotics (amoxicillin/clavulanate or ampicillin/sulbactam and extended-spectrum cephalosporins). Given the large size of our study populations and the potential for changes in antibiotic prescriptions over time, we adapted our approach to account for these variations. Specifically, PS matching was conducted separately for each calendar year of cohort entry. We pooled matched cohorts across calendar years and used Cox models stratified on calendar year to estimate summary HRs and corresponding 95% CIs.

For each country database, we first identified study cohorts, extracted information on covariates, fitted PS models, performed PS matching, and estimated outcome occurrence separately. We then used the Wald test to examine whether the effect estimates were statistically significant heterogeneity by database (p-value <0.05). To present overall findings across databases, we computed aSMD across databases for each covariate, using pooled means and SD. We also generated pooled incidence rates and pooled HRs with corresponding 95% CIs across databases with inverse variance weighting under random-effects models (Cochrane Training, 2023).

### Sensitivity and subgroup analyses

We performed several pre-specified analyses to ensure the robustness of our findings. First, we generated pooled outcome estimates across databases with inverse variance weighting under fixed-effect models. We then estimated rate differences with corresponding 95% CIs to provide intuitive, clinical insight. Third, we conducted sensitivity analyses by varying the follow-up duration to 60, 30, and 14 days to mitigate the potential impact from exposure misclassification. After that, we estimated incidence rates and HRs in each patient subgroup stratified on age at cohort entry (>65 or ≤65 years), sex (male and female), diabetes (yes or no), and ophthalmic comorbidities or medication use (yes or no). We then evaluated whether the incidence rates and association varied across different fluoroquinolones. Finally, using Taiwan NHIRD with information on where antibiotics were prescribed (outpatient visits, ED visits, or during hospitalizations), we examined whether the risk associated with fluoroquinolones changed materially by treatment setting.

For subgroup analyses by patient characteristic, fluoroquinolone, and treatment setting, we re-estimated PS and re-matched patients within each subgroup (Rassen et al., 2012; Wang et al., 2018).

## Results

### Study cohort

**FIGURE 1:**
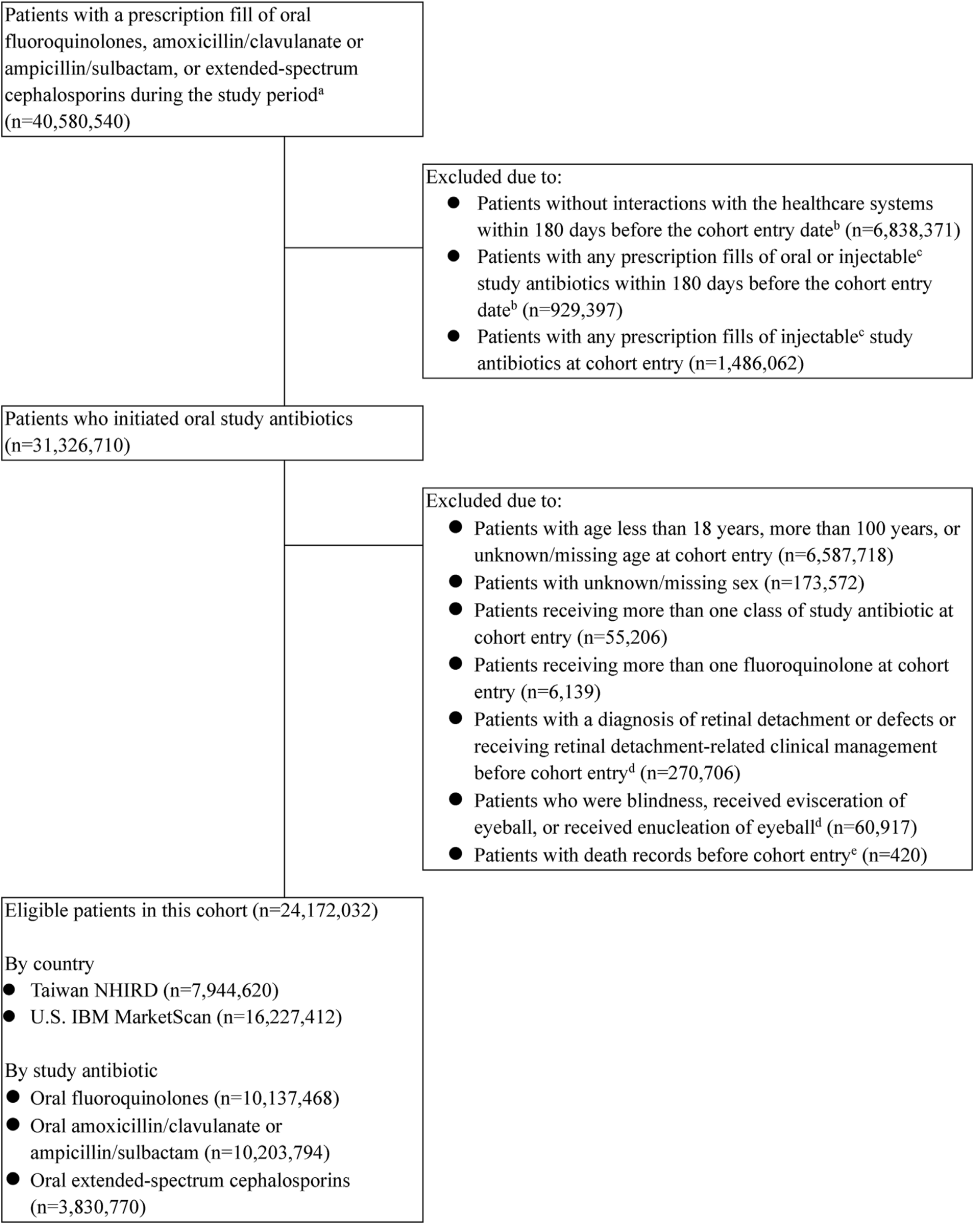
Flowchart of the study cohort assembly. U.S.—United States. ^a^Patients with study antibiotic use were identified from the Taiwanese data between 2009/1/1 and 2018/8/31 and from the U.S. data between 2011/1/1 and 2020/9/30. ^b^The cohort entry date was defined as the date of the first dispensing of a study antibiotic. ^c^Exclusion of baseline injectable antibiotic use was only conducted in the Taiwanese data. ^d^Information on retinal detachment or defects, retinal-related clinical management, blindness, or evisceration or enucleation of eyeball was examined as early as before the cohort entry (2008/1/1 for Taiwanese data and 2010/1/1 for the U.S. data). ^e^Death records were only available in the Taiwanese data.

Among 24,172,032 eligible patients comprising 7,944,620 insured Taiwanese (mean age [SD], 46 [18] years; 45% male) and 16,227,412 United States commercially insured individuals (mean age [SD], 47 [16] years; 40% male), 10,137,468 initiated fluoroquinolones, 10,203,794 initiated amoxicillin/clavulanate or ampicillin/sulbactam, and 3,830,770 initiated extended-spectrum cephalosporins (Figure 1; Supplementary Table S7). Overall, ciprofloxacin (55%) was the most commonly used fluoroquinolone, followed by levofloxacin (26%) and ofloxacin (12%). Amoxicillin/clavulanate (99%) predominated among the groups of amoxicillin/clavulanate or ampicillin/sulbactam. Cefdinir (32%), cefuroxime (29%), and cefaclor (27%) were the top three prescribed extended-spectrum cephalosporins (Supplementary Table S8).

Before PS matching, fluoroquinolone initiators were older (mean age: 49 years versus 44 years) but had less males (39% versus 45%) than amoxicillin/clavulanate or ampicillin/sulbactam initiators. In terms of comorbidities, fluoroquinolone initiators were more likely to have genitourinary tract infections, intra-abdominal infections, hypertension, hyperlipidemia, diabetes, and a higher Deyo version of the Charlson comorbidity index than amoxicillin/clavulanate or ampicillin/sulbactam initiators. Fluoroquinolone initiators were also more likely to receive angiotensin-converting enzyme inhibitors or angiotensin II receptor blockers, beta blocking agents, diuretics, and statins, and they had more frequent outpatient visits (Table 1; Supplementary Tables S9, S10). Similar findings were observed when comparing fluoroquinolone initiators and extended-spectrum cephalosporin initiators (Table 2; Supplementary Tables S11, S12).

**TABLE 1:** Select patient characteristics among initiators of fluoroquinolones and initiators of amoxicillin/clavulanate or ampicillin/sulbactam before and after propensity score matching.

Variable^b^	Before matching (n = 20,341,262)			After matching (n = 8,926,482)		
	Fluoroquinolone	Amoxicillin/clavulanate or ampicillin/sulbactam	aSMD^a^	Fluoroquinolone	Amoxicillin/clavulanate or ampicillin/sulbactam	aSMD^a^
	n = 10,137,468	n = 10,203,794		n = 4,463,241	n = 4,463,241	
Demographics						
Age in years, mean (SD)	49.38 (17.15)	44.13 (16.13)	0.315	48.60 (17.14)	47.91 (16.73)	0.041
Male	3,976,719 (39.23)	4,639,431 (45.47)	−0.127	1,909,099 (42.77)	1,884,812 (42.23)	0.011
Acute infection diagnoses, n (%)						
Lower respiratory tract infections^c^	541,268 (5.34)	432,093 (4.23)	0.052	282,339 (6.33)	271,306 (6.08)	0.010
Genitourinary tract infections	2,521,092 (24.87)	233,576 (2.29)	0.698	254,187 (5.70)	232,637 (5.21)	0.021
Skin, soft tissue, and bone infections^d^	309,667 (3.05)	767,732 (7.52)	−0.201	237,906 (5.33)	235,445 (5.28)	0.002
Intra-abdominal infections	237,144 (2.34)	99,695 (0.98)	0.107	111,418 (2.50)	86,013 (1.93)	0.039
Septicemia	100,762 (0.99)	78,560 (0.77)	0.024	40,565 (0.91)	34,279 (0.77)	0.015
Acute respiratory tract infections^e^	2,279,051 (22.48)	5,165,344 (50.62)	−0.611	1,632,399 (36.57)	1,622,661 (36.36)	0.005
Ophthalmic comorbidities, n (%)						
Endophthalmitis	2,675 (0.03)	2,481 (0.02)	0.006	1,356 (<0.01)	1,269 (<0.01)	0.001
Myopia	56,240 (0.55)	64,793 (0.63)	−0.010	26,336 (0.59)	25,722 (0.58)	0.002
Diabetic retinopathy	53,603 (0.53)	37,943 (0.37)	0.024	23,687 (0.53)	22,430 (0.50)	0.004
Retinal vascular occlusion	13,517 (0.13)	7,522 (0.07)	0.019	4,952 (<0.01)	4,484 (<0.01)	0.003
Other retinal disorders	183,506 (1.81)	107,368 (1.05)	0.064	69,874 (1.57)	63,706 (1.43)	0.011
Chorioretinitis	2,142 (0.02)	1,516 (0.01)	0.008	853 (<0.01)	797 (<0.01)	0.001
Glaucoma	317,844 (3.14)	196,196 (1.92)	0.078	119,832 (2.68)	111,239 (2.49)	0.012
Cataract	452,264 (4.46)	313,378 (3.07)	0.073	203,538 (4.56)	190,518 (4.27)	0.014
Vitreous body disorders	121,024 (1.19)	89,361 (0.88)	0.031	50,611 (1.13)	47,910 (1.07)	0.006
Severe head trauma^f^	4,696 (0.05)	12,203 (0.12)	−0.024	2,699 (<0.01)	2,622 (<0.01)	0.001
Severe eye trauma^g^	7,893 (0.08)	9,450 (0.09)	−0.003	4,109 (<0.01)	3,949 (<0.01)	0.001
Intravitreal injection	22,685 (0.22)	9,557 (0.09)	0.033	7,168 (<0.01)	6,271 (<0.01)	0.005
Other major eye surgery^h^	677,089 (6.68)	489,007 (4.79)	0.081	267,472 (5.99)	253,758 (5.69)	0.013
Non-ophthalmic comorbidities, n (%)						
Hypertension	2,653,533 (26.18)	1,907,918 (18.7)	0.180	1,137,004 (25.47)	1,086,515 (24.34)	0.026
Ischemic heart disease	552,265 (5.45)	362,286 (3.55)	0.092	244,782 (5.48)	225,363 (5.05)	0.019
Myocardial infarction	79,803 (0.79)	43,604 (0.43)	0.046	30,872 (0.69)	27,412 (0.61)	0.010
Cardiac dysrhythmia/atrial fibrillation	460,643 (4.54)	284,122 (2.78)	0.094	188,641 (4.23)	170,241 (3.81)	0.021
Congestive heart failure	207,471 (2.05)	119,726 (1.17)	0.070	89,826 (2.01)	78,730 (1.76)	0.018
Cerebrovascular disease	307,141 (3.03)	208,260 (2.04)	0.063	138,493 (3.10)	126,369 (2.83)	0.016
Peripheral vascular disease	137,066 (1.35)	67,081 (0.66)	0.069	52,695 (1.18)	46,657 (1.05)	0.013
Hyperlipidemia	2,069,703 (20.42)	1,515,166 (14.85)	0.147	838,508 (18.79)	817,436 (18.31)	0.012
Diabetes mellitus	1,181,722 (11.66)	861,880 (8.45)	0.107	519,137 (11.63)	493,615 (11.06)	0.018
Chronic lung disease	1,043,190 (10.29)	902,959 (8.85)	0.049	544,800 (12.21)	525,929 (11.78)	0.013
Chronic liver disease	153,491 (1.51)	165,805 (1.62)	−0.009	96,371 (2.16)	91,351 (2.05)	0.008
Gastritis or peptic ulcer disease	805,048 (7.94)	761,234 (7.46)	0.018	490,979 (11.00)	464,348 (10.40)	0.019
Chronic kidney disease	245,969 (2.43)	139,524 (1.37)	0.078	104,071 (2.33)	92,613 (2.08)	0.017
aDCSI (0–13), mean (SD)	0.12 (0.62)	0.08 (0.47)	0.073	0.12 (0.62)	0.11 (0.58)	0.019
Deyo version of Charlson comorbidity index (0–37), mean (SD)	0.66 (1.40)	0.46 (1.10)	0.159	0.7 (1.39)	0.65 (1.35)	0.039
Ophthalmic medication use, n (%)						
Anti-bacterials	769,267 (7.59)	989,675 (9.70)	−0.075	486,053 (10.89)	470,762 (10.55)	0.011
Anti-virals	2,979 (0.03)	2,772 (0.03)	<0.001	1,539 (<0.01)	1,422 (<0.01)	0.001
Corticosteroids	522,162 (5.15)	596,015 (5.84)	−0.030	333,567 (7.47)	323,228 (7.24)	0.009
NSAIDs	33,859 (0.33)	18,252 (0.18)	0.030	11,944 (<0.01)	10,996 (<0.01)	0.004
Anti-glaucoma	316,738 (3.12)	264,448 (2.59)	0.032	163,882 (3.67)	154,121 (3.45)	0.012
Anti-neovascularization agents	246 (<0.01)	233 (<0.01)	<0.001	125 (<0.01)	126 (<0.01)	<0.001
Non-ophthalmic medication use, n (%)						
ACEIs/ARBs	2,113,635 (20.85)	1,530,837 (15.00)	0.153	882,521 (19.77)	851,277 (19.07)	0.018
Beta blocking agents	1,425,296 (14.06)	998,887 (9.79)	0.132	618,963 (13.87)	585,129 (13.11)	0.022
Calcium channel blockers	1,159,878 (11.44)	882,877 (8.65)	0.093	544,647 (12.2)	516,387 (11.57)	0.020
Diuretics	1,507,354 (14.87)	983,114 (9.63)	0.160	606,966 (13.6)	579,732 (12.99)	0.018
Other anti-hypertensive agents	232,411 (2.29)	147,252 (1.44)	0.063	107,717 (2.41)	98,888 (2.22)	0.013
Nitrates	199,509 (1.97)	149,999 (1.47)	0.038	101,868 (2.28)	94,338 (2.11)	0.012
Anti-arrhythmic agents	91,708 (0.90)	89,588 (0.88)	0.002	51,417 (1.15)	48,161 (1.08)	0.007
Digoxin	66,792 (0.66)	39,054 (0.38)	0.039	28,851 (0.65)	25,773 (0.58)	0.009
Aspirin	240,810 (2.38)	269,810 (2.64)	−0.017	162,692 (3.65)	155,243 (3.48)	0.009
Clopidogrel	205,526 (2.03)	115,651 (1.13)	0.072	82,307 (1.84)	75,143 (1.68)	0.012
Warfarin	153,505 (1.51)	85,500 (0.84)	0.062	62,278 (1.40)	55,434 (1.24)	0.013
New oral anticoagulants	59,743 (0.59)	39,805 (0.39)	0.029	23,924 (0.54)	21,894 (<0.01)	0.006
Statins	1,822,670 (17.98)	1,222,742 (11.98)	0.169	709,343 (15.89)	682,775 (15.30)	0.016
Fibrates	232,991 (2.30)	172,093 (1.69)	0.044	102,934 (2.31)	99,036 (2.22)	0.006
Insulin	276,915 (2.73)	202,344 (1.98)	0.049	124,252 (2.78)	117,624 (2.64)	0.009
Metformin	750,754 (7.41)	599,859 (5.88)	0.061	341,168 (7.64)	329,794 (7.39)	0.010
Sulfonylurea	396,160 (3.91)	310,474 (3.04)	0.048	194,292 (4.35)	185,499 (4.16)	0.010
Alpha-glucosidase inhibitors	36,934 (0.36)	41,885 (0.41)	−0.008	25,566 (0.57)	24,687 (0.55)	0.003
Thiazolidinedione	108,134 (1.07)	76,377 (0.75)	0.034	49,166 (1.10)	47,050 (1.05)	0.005
Glinides	29,190 (0.29)	27,661 (0.27)	0.004	18,188 (<0.01)	17,116 (<0.01)	0.004
DPP-4is	184,555 (1.82)	151,413 (1.48)	0.027	88,068 (1.97)	84,645 (1.90)	0.006
GLP-1 RAs	54,842 (0.54)	42,294 (0.41)	0.019	21,320 (<0.01)	21,084 (<0.01)	0.001
SGLT2is	26,122 (0.26)	29,533 (0.29)	−0.006	11,114 (<0.01)	11,048 (<0.01)	<0.001
Non-study antibiotics	4,792,340 (47.27)	8,650,670 (84.78)	−0.862	3,587,473 (80.38)	3,574,750 (80.09)	0.007
Systemic corticosteroids	1,798,398 (17.74)	1,971,070 (19.32)	−0.041	1,045,363 (23.42)	1,036,405 (23.22)	0.005
Resource utilization, mean (SD)						
No. of outpatient visits	8.47 (8.91)	7.52 (7.58)	0.115	9.49 (8.65)	9.23 (8.89)	0.030
No. of hospitalizations	0.12 (0.45)	0.09 (0.39)	0.071	0.14 (0.49)	0.12 (0.48)	0.043
No. of ophthalmological outpatient visits	0.23 (0.87)	0.21 (0.84)	0.023	0.29 (1.00)	0.27 (1.00)	0.014
No. of ophthalmological hospitalizations	<0.001 (0.02)	<0.001 (0.01)	<0.001	<0.01 (0.02)	<0.01 (0.02)	<0.001

ACEIs, angiotensin-converting enzyme inhibitors; aDCSI, adapted diabetes complications severity index; ARBs, angiotensin II receptor blockers; aSMD, absolute standardized mean difference; DPP-4is, dipeptidyl peptidase-4 inhibitors; GLP-1 RAs, glucagon-like peptide-1 receptor agonists; NSAIDs, non-steroidal anti-inflammatory drugs; SD, standard deviation; SGLT-2is, sodium glucose cotransporter 2 inhibitors.

^a^ aSMD, estimated for each covariate based on pooled means and SD across databases.

^b^ Data presented as the number and proportion of patents unless otherwise specified.

^c^ Lower respiratory tract infections included pneumonia, influenza, empyema, and lung and mediastinum abscess.

^d^ Bone infections included osteomyelitis and necrotizing fasciitis.

^e^ Acute respiratory tract infections included acute nasopharyngitis, acute sinusitis, acute pharyngitis, acute tonsillitis, acute laryngitis and tracheitis, acute upper respiratory infections of multiple or unspecified sites, and acute bronchitis and bronchiolitis.

^f^ Severe head trauma included fracture of skull base, orbital floor, and other skull or facial bones.

^g^ Severe eye trauma included contusion of orbital tissues and eyeball, unspecified contusion of eye, penetrating wound of orbit, open wound of eyeball, and burn of eye.

^h^ Other major eye surgery included operations on the cornea, iris, ciliary body, sclera, anterior chamber, extraocular muscles, orbit, and eyeball.

**TABLE 2:** Selected patient characteristics among initiators of fluoroquinolones and initiators of extended-spectrum cephalosporins before and after propensity score matching.

	Before matching (n = 13,968,238)			After matching (n = 7,010,718)		
Variable^b^	Fluoroquinolone	Extended-spectrum cephalosporin	aSMD^a^	Fluoroquinolone	Extended-spectrum cephalosporin	aSMD^a^
	n = 10,137,468	n = 3,830,770		n = 3,505,359	n = 3,505,359	
Demographics						
Age in years, mean (SD)	49.38 (17.15)	44.83 (16.85)	0.268	47.27 (16.68)	45.97 (16.71)	0.078
Male	3,976,719 (39.23)	1,499,457 (39.14)	0.002	1,424,924 (40.65)	1,400,525 (39.95)	0.014
Acute infection diagnoses, n (%)						
Lower respiratory tract infections^c^	541,268 (5.34)	141,670 (3.70)	0.079	168,600 (4.81)	138,848 (3.96)	0.041
Genitourinary tract infections	2,521,092 (24.87)	317,307 (8.28)	0.458	304,765 (8.69)	317,025 (9.04)	−0.012
Skin, soft tissue, and bone infections^d^	309,667 (3.05)	119,660 (3.12)	−0.004	133,947 (3.82)	117,451 (3.35)	0.025
Intra-abdominal infections	237,144 (2.34)	16,739 (0.44)	0.163	21,030 (0.60)	16,527 (<0.01)	0.018
Septicemia	100,762 (0.99)	36,781 (0.96)	0.003	40,871 (1.17)	32,416 (0.92)	0.024
Acute respiratory tract infections^e^	2,279,051 (22.48)	2,180,145 (56.91)	−0.752	1,867,824 (53.28)	1,855,069 (52.92)	0.007
Ophthalmic comorbidities, n (%)						
Endophthalmitis	2,675 (0.03)	891 (0.02)	0.006	984 (0.03)	844 (<0.01)	0.002
Myopia	56,240 (0.55)	23,458 (0.61)	−0.008	24,594 (0.70)	21,122 (0.60)	0.012
Diabetic retinopathy	53,603 (0.53)	14,169 (0.37)	0.024	17,269 (0.49)	13,855 (<0.01)	0.015
Retinal vascular occlusion	13,517 (0.13)	3,245 (0.08)	0.015	4,009 (0.11)	3,125 (<0.01)	0.008
Other retinal disorders	183,506 (1.81)	47,125 (1.23)	0.047	56,539 (1.61)	45,207 (1.29)	0.027
Chorioretinitis	2,142 (0.02)	614 (0.02)	<0.001	684 (0.02)	560 (<0.01)	0.003
Glaucoma	317,844 (3.14)	81,130 (2.12)	0.064	96,285 (2.75)	77,929 (2.22)	0.034
Cataract	452,264 (4.46)	132,436 (3.46)	0.051	154,085 (4.40)	128,718 (3.67)	0.037
Disorders of vitreous body	121,024 (1.19)	35,968 (0.94)	0.024	41,707 (1.19)	34,325 (0.98)	0.020
Severe head trauma^f^	4,696 (0.05)	1,691 (0.04)	0.005	2,035 (0.06)	1,616 (<0.01)	0.005
Severe eye trauma^g^	7,893 (0.08)	2,825 (0.07)	0.004	3,215 (0.09)	2,681 (<0.01)	0.005
Intravitreal injection	22,685 (0.22)	4,468 (0.12)	0.024	5,852 (0.17)	4,315 (<0.01)	0.012
Other major eye surgery^h^	677,089 (6.68)	168,721 (4.40)	0.100	195,576 (5.58)	156,361 (4.46)	0.051
Non-ophthalmic comorbidities, n (%)						
Hypertension	2,653,533 (26.18)	758,527 (19.8)	0.152	873,461 (24.92)	734,145 (20.94)	0.095
Ischemic heart disease	552,265 (5.45)	155,104 (4.05)	0.066	185,826 (5.30)	151,110 (4.31)	0.046
Myocardial infarction	79,803 (0.79)	19,282 (0.50)	0.036	24,611 (0.70)	18,649 (0.53)	0.022
Cardiac dysrhythmia/atrial fibrillation	460,643 (4.54)	129,161 (3.37)	0.060	156,980 (4.48)	122,080 (3.48)	0.051
Congestive heart failure	207,471 (2.05)	55,723 (1.45)	0.046	70,594 (2.01)	54,098 (1.54)	0.036
Cerebrovascular disease	307,141 (3.03)	86,341 (2.25)	0.049	104,172 (2.97)	84,159 (2.40)	0.035
Peripheral vascular disease	137,066 (1.35)	27,829 (0.73)	0.061	35,932 (1.03)	27,334 (0.78)	0.026
Hyperlipidemia	2,069,703 (20.42)	591,142 (15.43)	0.130	666,631 (19.02)	570,019 (16.26)	0.072
Diabetes mellitus	1,181,722 (11.66)	333,767 (8.71)	0.098	394,154 (11.24)	324,447 (9.26)	0.066
Chronic lung disease	1,043,190 (10.29)	358,286 (9.35)	0.032	411,073 (11.73)	340,652 (9.72)	0.065
Chronic liver disease	153,491 (1.51)	64,024 (1.67)	−0.013	70,561 (2.01)	62,661 (1.79)	0.017
Gastritis or peptic ulcer disease	805,048 (7.94)	318,862 (8.32)	−0.014	344,151 (9.82)	313,483 (8.94)	0.030
Chronic kidney disease	245,969 (2.43)	62,803 (1.64)	0.056	77,503 (2.21)	61,342 (1.75)	0.033
aDCSI (0–13), mean (SD)	0.12 (0.62)	0.08 (0.49)	0.072	0.11 (0.58)	0.09 (0.51)	0.050
Deyo version of Charlson comorbidity index (0–37), mean (SD)	0.66 (1.40)	0.48 (1.12)	0.142	0.62 (1.27)	0.51 (1.15)	0.092
Ophthalmic medication use, n (%)						
Anti-bacterials	769,267 (7.59)	387,395 (10.11)	−0.089	402,781 (11.49)	360,867 (10.29)	0.038
Anti-virals	2,979 (0.03)	1,033 (0.03)	<0.001	1,241 (0.04)	987 (<0.01)	0.004
Corticosteroids	522,162 (5.15)	258,297 (6.74)	−0.067	272,082 (7.76)	246,446 (7.03)	0.028
NSAIDs	33,859 (0.33)	7,797 (0.20)	0.025	9,580 (0.27)	7,491 (<0.01)	0.012
Anti-glaucoma	316,738 (3.12)	114,618 (2.99)	0.008	126,423 (3.61)	111,527 (3.18)	0.023
Anti-neovascularization agents	246 (<0.01)	98 (<0.01)	<0.001	104 (<0.01)	92 (<0.01)	0.001
Non-ophthalmic medication use, n (%)						
ACEIs/ARBs	2,113,635 (20.85)	603,088 (15.74)	0.132	694,309 (19.81)	582,423 (16.62)	0.083
Beta blocking agents	1,425,296 (14.06)	423,485 (11.05)	0.091	491,871 (14.03)	407,400 (11.62)	0.072
Calcium channel blockers	1,159,878 (11.44)	360,032 (9.40)	0.067	416,843 (11.89)	350,507 (10.00)	0.061
Diuretics	1,507,354 (14.87)	404,829 (10.57)	0.129	472,676 (13.48)	389,165 (11.10)	0.073
Other anti-hypertensive agents	232,411 (2.29)	63,313 (1.65)	0.046	76,007 (2.17)	61,678 (1.76)	0.029
Nitrates	199,509 (1.97)	61,189 (1.60)	0.028	72,616 (2.07)	60,095 (1.71)	0.026
Anti-arrhythmic agents	91,708 (0.90)	41,175 (1.07)	−0.017	45,506 (1.30)	38,383 (1.09)	0.019
Digoxin	66,792 (0.66)	19,021 (0.50)	0.021	23,791 (0.68)	18,292 (0.52)	0.020
Aspirin	240,810 (2.38)	102,636 (2.68)	−0.019	113,425 (3.24)	100,964 (2.88)	0.021
Clopidogrel	205,526 (2.03)	50,493 (1.32)	0.055	61,967 (1.77)	49,383 (1.41)	0.029
Warfarin	153,505 (1.51)	45,815 (1.20)	0.027	58,402 (1.67)	42,468 (1.21)	0.038
New oral anticoagulants	59,743 (0.59)	18,175 (0.47)	0.017	22,068 (0.63)	16,702 (<0.01)	0.021
Statins	1,822,670 (17.98)	490,945 (12.82)	0.143	567,729 (16.20)	474,502 (13.54)	0.075
Fibrates	232,991 (2.30)	69,750 (1.82)	0.034	81,346 (2.32)	68,010 (1.94)	0.026
Insulin	276,915 (2.73)	74,465 (1.94)	0.052	90,612 (2.58)	72,089 (2.06)	0.035
Metformin	750,754 (7.41)	228,102 (5.95)	0.059	264,324 (7.54)	220,116 (6.28)	0.050
Sulfonylurea	396,160 (3.91)	124,260 (3.24)	0.036	144,710 (4.13)	121,725 (3.47)	0.034
Alpha-glucosidase inhibitors	36,934 (0.36)	16,664 (0.44)	−0.013	18,307 (0.52)	16,370 (<0.01)	0.008
Thiazolidinedione	108,134 (1.07)	31,699 (0.83)	0.025	37,130 (1.06)	31,146 (0.89)	0.017
Glinides	29,190 (0.29)	10,891 (0.28)	0.002	12,324 (0.35)	10,757 (<0.01)	0.008
DPP-4is	184,555 (1.82)	58,793 (1.53)	0.023	68,681 (1.96)	57,438 (1.64)	0.024
GLP-1 RAs	54,842 (0.54)	15,856 (0.41)	0.019	18,693 (0.53)	14,690 (<0.01)	0.017
SGLT2is	26,122 (0.26)	9,564 (0.25)	0.002	10,592 (0.30)	8,511 (<0.01)	0.011
Non-study antibiotics	4,792,340 (47.27)	1,736,005 (45.32)	0.039	1,685,400 (48.08)	1,641,798 (46.84)	0.025
Systemic corticosteroids	1,798,398 (17.74)	821,805 (21.45)	−0.094	849,064 (24.22)	749,668 (21.39)	0.068
Resource utilization, mean (SD)						
No. of outpatient visits	8.47 (8.91)	8.10 (7.86)	0.044	9.27 (8.62)	8.34 (8.00)	0.111
No. of hospitalizations	0.12 (0.45)	0.09 (0.39)	0.071	0.12 (0.46)	0.09 (0.40)	0.073
No. of ophthalmological outpatient visits	0.23 (0.87)	0.24 (0.91)	−0.011	0.29 (0.99)	0.26 (0.94)	0.031
No. of ophthalmological hospitalizations	<0.001 (0.02)	<0.001 (0.01)	<0.001	<0.01 (0.02)	<0.01 (0.02)	<0.001

ACEIs, angiotensin-converting enzyme inhibitors; aDCSI, adapted diabetes complications severity index; ARBs, angiotensin II receptor blockers; aSMD, absolute standardized mean difference; DPP-4is, dipeptidyl peptidase-4 inhibitors; GLP-1 RAs, glucagon-like peptide-1 receptor agonists; NSAIDs, non-steroidal anti-inflammatory drugs; SD, standard deviation; SGLT-2is, sodium glucose cotransporter 2 inhibitors.

^a^ aSMD, estimated for each covariate based on pooled means and SD, across databases.

^b^ Data presented as the number and proportion of patents unless otherwise specified.

^c^ Lower respiratory tract infections included pneumonia, influenza, empyema, and lung and mediastinum abscess.

^d^ Bone infections included osteomyelitis and necrotizing fasciitis.

^e^ Acute respiratory tract infections included acute nasopharyngitis, acute sinusitis, acute pharyngitis, acute tonsillitis, acute laryngitis and tracheitis, acute upper respiratory infection of multiple or unspecified sites, and acute bronchitis and bronchiolitis.

^f^ Severe head trauma included fracture of skull base, orbital floor, and other skull or facial bones.

^g^ Severe eye trauma included contusion of orbital tissues and eyeball, unspecified contusion of eye, penetrating wound of orbit, open wound of eyeball, and burn of the eye.

^h^ Other major eye surgery included operations on the cornea, iris, ciliary body, sclera, anterior chamber, extraocular muscles, orbit, and eyeball.

Fluoroquinolones or amoxicillin/clavulanate or ampicillin/sulbactam were initiated by 8,926,482 patients, and 7,010,718 patients initiating fluoroquinolones or extended-spectrum cephalosporins in each pairwise PS-matched cohort. The aSMD between fluoroquinolones and comparison antibiotics after PS matching were <0.1, indicating well-balanced baseline characteristics after PS matching (Tables 1, 2; Supplementary Tables S9, S12).

### Risk of RRD associated with fluoroquinolones compared to comparison antibiotics

Overall, 2,077 patients experienced RRD within 90 days of cohort entry. The crude incidence rates were 0.34 (95% CI, 0.18–0.65) for fluoroquinolones, 0.32 (95% CI, 0.29–0.35) for amoxicillin/clavulanate or ampicillin/sulbactam, and 0.32 (95% CI, 0.23–0.43) for extended-spectrum cephalosporins per 1,000 person-years (Tables 3, 4; Supplementary Tables S13, S14). The crude HRs for fluoroquinolones were 1.06 (95% CI, 0.59–1.90) versus amoxicillin/clavulanate or ampicillin/sulbactam, and 1.06 (95% CI, 0.72–1.57) versus extended-spectrum cephalosporins (Tables 3, 4; Supplementary Tables S15, S16).

**TABLE 3:** Risk of rhegmatogenous retinal detachment comparing fluoroquinolones to amoxicillin/clavulanate or ampicillin/sulbactam before and after propensity score matching.

	Before matching (n = 20,341,262)		After matching (n = 8,926,482)	
	Fluoroquinolone	Amoxicillin/clavulanate or ampicillin/sulbactam	Fluoroquinolone	Amoxicillin/clavulanate or ampicillin/sulbactam
No. of patients	10,137,468	10,203,794	4,463,241	4,463,241
No. of events	990	784	374	378
Total person-days	882,720,271	890,443,072	391,502,360	390,702,271
Mean follow-up days, mean (SD)	87.08 (12.74)	87.27 (12.43)	87.72 (11.32)	87.54 (11.79)
Incidence rate (95% CI)^a^ ^,^ ^b^	0.34 (0.18, 0.65)	0.32 (0.29, 0.35)	0.33 (0.19, 0.56)	0.35 (0.26, 0.46)
HR (95% CI)^b^	1.06 (0.59, 1.90)	Ref	0.97 (0.76, 1.23)	Ref

CI, confidence intervals; SD, standard deviation.

^a^ Per 1,000 person-years.

^b^ Data pooled across databases using random-effects meta-analysis.

**TABLE 4:** Risk of rhegmatogenous retinal detachment comparing fluoroquinolones to extended-spectrum cephalosporins before and after propensity score matching.

	Before matching (n = 13,968,238)		After matching (n = 7,010,718)	
	Fluoroquinolone	Extended-spectrum cephalosporins	Fluoroquinolone	Extended-spectrum cephalosporins
No. of patients	10,137,468	3,830,770	3,505,359	3,505,359
No. of events	990	303	316	292
Total person-days	882,720,271	334,940,182	306,934,986	306,769,892
Mean follow-up days, mean (SD)	87.08 (12.74)	87.43 (12.06)	87.56 (11.70)	87.52 (11.88)
Incidence rate (95% CI)^a^ ^,^ ^b^	0.34 (0.18, 0.65)	0.32 (0.23, 0.43)	0.36 (0.22, 0.57)	0.34 (0.22, 0.50)
HR (95% CI)^b^	1.06 (0.72, 1.57)	Ref	1.08 (0.92, 1.27)	Ref

CI, confidence interval; SD, standard deviation.

^a^ Per 1,000 person-years.

^b^ Data pooled across databases using random-effects meta-analysis.

After PS matching, similar RRD incidence rates were observed between fluoroquinolones and amoxicillin/clavulanate or ampicillin/sulbactam initiators (0.33 [95% CI, 0.19–0.56] versus 0.35 [95% CI, 0.26–0.46] per 1,000 person-years), yielding a HR of 0.97 (95% CI, 0.76–1.23) (Table 3). The RRD incidence rates were also similar when comparing fluoroquinolones to extended-spectrum cephalosporins (0.36 [95% CI, 0.22–0.57] versus 0.34 [95% CI, 0.22–0.50] per 1,000 person-years; HR, 1.08 [95% CI, 0.92–1.27]) (Table 4).

For each country database’s effect estimates after PS matching, HRs for fluoroquinolones versus amoxicillin/clavulanate or ampicillin/sulbactam were 0.84 (95% CI, 0.66–1.07) in the Taiwan NHIRD and 1.08 (95% CI, 0.90–1.29) in the United States IBM MarketScan Database (p-value for heterogeneity testing: 0.101, Supplementary Table S15). Similarly, HRs for fluoroquinolones versus extended-spectrum cephalosporins did not suggest differential risk across databases (1.02 [95% CI, 0.77–1.35] in the Taiwanese data and 1.11 [95% CI, 0.92–1.35] in the United States data; p-value for heterogeneity testing: 0.628; Supplementary Table S16).

### Findings of sensitivity and subgroup analyses

The HRs after PS matching for fluoroquinolones using fixed-effect models were similar to main analyses using random-effects models (0.99 [95% CI, 0.85–1.14] versus amoxicillin/clavulanate or ampicillin/sulbactam and 1.08 [95% CI, 0.92–1.27] versus extended-spectrum cephalosporins). The rate differences after PS matching were −0.008 (95% CI, −0.085 to 0.068) versus amoxicillin/clavulanate or ampicillin/sulbactam and 0.026 (95% CI, −0.014–0.065) versus extended-spectrum cephalosporins per 1,000 person-years. The findings using various follow-up durations remain consistent with primary analyses. Specifically, HRs after PS matching versus amoxicillin/clavulanate or ampicillin/sulbactam were 1.02 (95% CI, 0.85–1.22), 0.98 (95% CI, 0.54–1.77), and 0.91 (95% CI, 0.44–1.89) during 60-, 30-, and 14-day follow-ups, respectively (Supplementary Table S17). Similar results were observed for fluoroquinolones versus extended-spectrum cephalosporins (HRs after PS matching: 1.10 [95% CI, 0.86–1.40] during 60-day follow-up and 1.06 [95% CI, 0.81–1.40] during 30-day follow-up), although the relative risk was numerically higher and imprecise during 14-day follow-up (1.45 [95% CI, 0.89–2.36]) (Supplementary Table S18).

**FIGURE 2:**
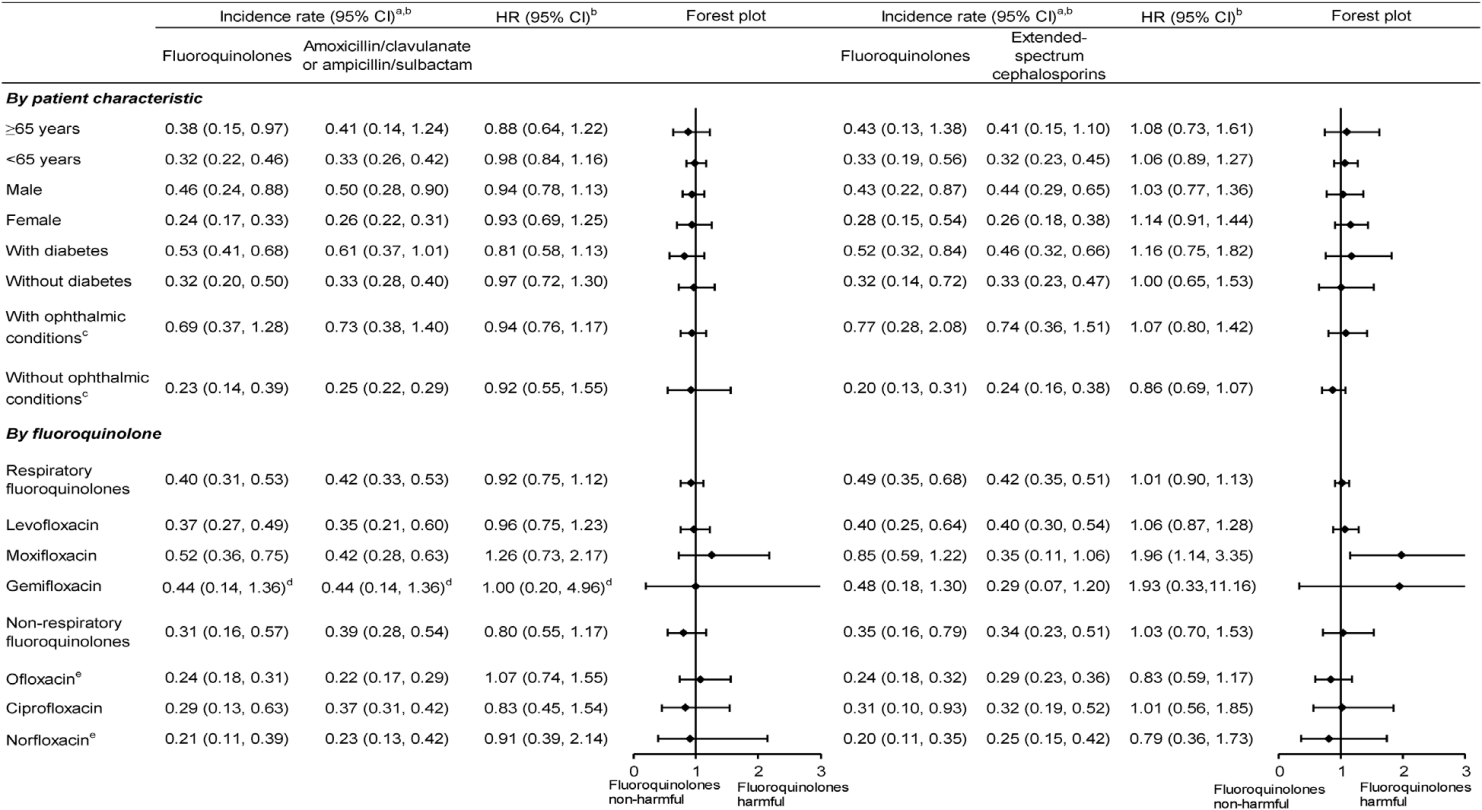
Subgroup analyses of the incidence rate and HR of rhegmatogenous retinal detachment comparing fluoroquinolones versus comparison antibiotics after propensity score matching. CI, confidence intervals; HR,— hazard ratios; ^a^Per 1,000 person-years. ^b^Data are pooled across databases using random-effects meta-analysis. ^c^Ophthalmic conditions included ophthalmic comorbidities and ophthalmic medication use. ^d^Only Taiwanese data contributed to the analysis, given no cases receiving amoxicillin/clavulanate or ampicillin/sulbactam in the United States data. ^e^Only Taiwanese data contributed to the analysis, given few patients receiving ofloxacin and norfloxacin in the United States data.

In the subgroup analyses stratified by patient characteristic, incidence rates after PS matching for fluoroquinolone initiators were higher in elderly, males, and those with diabetes or ophthalmic conditions (ranging from 0.38 to 0.77 per 1,000 person-years) than that in non-elderly, females, and those without diabetes or ophthalmic condition (0.20–0.33 per 1,000 person-years). Respiratory fluoroquinolone initiators also showed higher incidence rates after PS matching (0.37–0.85 per 1,000 person-years) than non-respiratory fluoroquinolone initiators (0.20–0.35 per 1,000 person-years). However, there was generally no increased risk comparing fluoroquinolones versus either comparison antibiotic in each patient subgroup. Notably, the risk associated with moxifloxacin (versus both comparison antibiotics) and gemifloxacin (versus extended-spectrum cephalosporins) appeared to be more elevated than other fluoroquinolones, although the 95% CIs were wide (Figure 2; Supplementary Tables S19, S22). Based on the Taiwanese data, around 80% of study patients received study or comparison antibiotics from outpatient visits and 20% received antibiotic treatment from ED visits or during hospitalization. The HRs of fluoroquinolones versus comparison antibiotics did not differ by treatment setting (Supplementary Table S23).

## Discussion

Leveraging claims data of >24 million adult patients from Taiwan and United States daily practice settings, this cohort study found a low and similar RRD risk across study antibiotic groups, including fluoroquinolones, after controlling for numerous potential confounders. The RRD incidence rates for fluoroquinolones were below 0.36 in the general population and varied 0.37–0.85 per 1,000 person-years in patients considered at high risk, such as the elderly, males, those with diabetes or ophthalmic conditions, and those receiving respiratory fluoroquinolones. The HRs did not suggest an increased risk associated with fluoroquinolone use and showed no significant variations across different countries, patient characteristics, types of fluoroquinolones, treatment settings, or length of follow-up.

### Comparison with existing real-world evidence

#### Methodological challenges

While several observational studies have previously reported a 1.25- to 4.50-fold elevated RD risk (RRD mainly) with fluoroquinolone use compared to non-use of fluoroquinolones (see summary findings in Supplementary Figure S1), (Chui et al., 2014; Raguideau et al., 2016; Shin et al., 2018; Baek et al., 2018; Londhe et al., 2022; Etminan et al., 2012; Fife et al., 2014; Pasternak et al., 2013; Daneman et al., 2015), our findings do not support a significant RRD risk elevation. It is worth noting that earlier studies may have been influenced by residual confounding, as fluoroquinolone users often had conditions such as pneumonia (Daneman et al., 2015), diabetes (Pasternak et al., 2013; Daneman et al., 2015), retinopathy (Pasternak et al., 2013), and ophthalmologic visits (Pasternak et al., 2013) and tended to be male (Pasternak et al., 2013; Daneman et al., 2015); these are independently associated with increased RRD risk. For example, pneumonia may have accompanying severe cough precipitating RRD (Kuo et al., 2014). Proliferative diabetic retinopathy, ophthalmic disorders, and male gender are also risk factors for RRD (UpToDate^®^, 2023; Sodhi et al., 2008; Mitry et al., 2010a). Prior studies may not have adjusted well for these variables and thus overestimated the RRD risk associated with fluoroquinolones. By contrast, our study employed PS matching to comprehensively adjust for over 80 covariates, including 25 related to ophthalmic conditions and treatments, and it provided a robust comparison that revealed no elevated RRD risk with fluoroquinolones versus comparison antibiotics.

Furthermore, a Taiwanese cohort study comparing fluoroquinolones to amoxicillin showed a two-fold increase in risk (Kuo et al., 2014). However, amoxicillin was more likely to be used for mild infection and may not be comparable to fluoroquinolones (Gopalakrishnan et al., 2020). The present study selected amoxicillin/clavulanate or ampicillin/sulbactam or extended-spectrum cephalosporins as comparison antibiotics, which are potential alternative treatments based on clinical guideline suggestions. Our findings were consistent with the null association found in the few available active comparison studies (fluoroquinolones versus beta-lactam or macrolide antibiotics) (Eftekhari et al., 2014; Kapoor et al., 2014). Our approach was also in line with the recommendation of Douglas et al. (2016) and Lund et al. (2015) that a “similar control exposure” is useful for mitigating potential confounding.

#### Insights into absolute risk estimates

Three Western cohort studies reported crude incidence rates of RD ranging from 0.09 to 0.3 per 1,000 person-years 10–365 days after fluoroquinolone use (Pasternak et al., 2013; Eftekhari et al., 2014; Daneman et al., 2015). Our cohort study with Taiwanese and United States data showed a crude incidence rate of RRD of 0.34 per 1,000 person-years within 90 days after fluoroquinolone initiation (see Supplementary Table S24 for crude incidence rates across studies). In particular, our study provided additional information on adjusted incidence rates after carefully accounting for imbalance in patient characteristics between exposure groups (0.33 [fluoroquinolones] versus 0.35 [amoxicillin/clavulanate or ampicillin/sulbactam] and 0.36 [fluoroquinolones] versus 0.34 [extended-spectrum cephalosporins] per 1,000 person-years in each PS-matched cohort, respectively). The rate differences associated with fluoroquinolones were 0.008 fewer events versus amoxicillin/clavulanate or ampicillin/sulbactam and 0.026 more events versus extended-spectrum cephalosporins per 1,000 person-years. Therefore, even if there are harmful effects associated with fluoroquinolone use, this may not be clinically meaningful.

Our study further demonstrated higher incidence rates for fluoroquinolones in the elderly, males, patients with diabetes or ophthalmic conditions, and patients receiving respiratory fluoroquinolones (0.37–0.85 per 1,000 person-years). This may reflect a higher baseline risk profile for patients with potential risk factors of RRD. This was also in line with prior observations that patients using respiratory fluoroquinolones tend to be more vulnerable (Dong et al., 2024). However, there was no excess comparative risk comparing fluoroquinolones versus comparison antibiotics in each patient subgroup. Our findings provide comprehensive safety information for treatment decisions in various clinical settings.

### Exploring the link between collagen degradation and fluoroquinolone-associated RRD

Collagen is the substantial component of the extracellular matrix in tendon cells (Tsai et al., 2011; Chang HN. et al., 2012), the aorta (Berillis, 2013), and ocular structures (e.g., vitreoretinal interface) (Bu et al., 2015). Previous experimental studies have found that fluoroquinolones may increase the activity of matrix metalloproteinase, disrupt the integrity of collagen, and result in tendon rupture or aortopathy (Tsai et al., 2011; Chang HN. et al., 2012; LeMaire et al., 2022; LeMaire et al., 2018; Guzzardi et al., 2019). It is possible that fluoroquinolones may also impair the function of collagen in the vitreoretinal interface and the adhesion of the retina to surrounding tissues, leading to retinal break or even RRD. However, the major types of collagen in the tendon cells and aorta (types I and III) (Tsai et al., 2011; Chang HN. et al., 2012; Berillis, 2013) are different from that in the vitreoretinal interface (types II, IV, and VI) (Bu et al., 2015). Moreover, the lack of direct experimental evidence linking fluoroquinolones to vitreoretinal collagen degradation suggests that this risk may not be as pronounced as feared.

### Strengths and limitations of the present study

The present study had several unique strengths. First, to our knowledge, our cohort study including United States and Taiwanese data had the largest number of patients, yielding more precise absolute risk estimates and generalizable findings. Unlike prior meta-analyses which pooled results from various study designs using aggregated counts (Chui et al., 2015; Alves et al., 2016; Yu et al., 2019), our common protocol approach analyzing individual-level data mitigated heterogeneity issues of the designs when pooling different sources of data. Second, as mentioned above, although the United States data can only capture medication information from outpatient settings, the Taiwanese data can additionally ascertain medication use from ED visits and hospital admissions and distinguish oral and injectable antibiotics. The comparative risk associated with fluoroquinolones did not vary by country data; this minimized concerns about exposure misclassification and confounding by infection severity due to injectable antibiotic use. The comparative risk associated with fluoroquinolones also did not differ between outpatient visits and ED visits/during hospitalization (based on Taiwanese data); this mitigated the issue of confounding by infection severity due to treatment settings.

Third, our data demonstrated fluoroquinolone prescription patterns across geographic areas. Specifically, ciprofloxacin and levofloxacin were predominantly prescribed in the United States, while ofloxacin and norfloxacin were used more commonly in Taiwan (Supplementary Table S8). This allowed us to provide safety information for older fluoroquinolones not available in prior epidemiological studies. Fourth, we chose appropriate comparison antibiotics, adjusted for comprehensive covariate information, and generated risk estimates in each well-balanced PS-matched cohort. All of these lend support to our study’s validity.

Despite its strengths, we also acknowledge some limitations. First, we only examined the RRD risk associated with fluoroquinolone use during a 90-day follow-up. Future studies are necessary to assess the long-term safety of fluoroquinolones (e.g., multidrug-resistance tuberculosis requiring >12-month fluoroquinolone treatment) (Taiwan Center for Disease Control, 2022). Second, although our study had a large sample size to estimate the incidence rates stratified on patient characteristics, the event numbers were smaller when examining the effect of individual fluoroquinolones. For example, there were few RRD events in the gemifloxacin group, which may lead to imprecise risk estimates (Figure 2; Supplementary Tables S21, S22).

Finally, residual confounding may not be entirely ruled out in our results. For example, we observed a higher HR associated with moxifloxacin, especially versus extended-spectrum cephalosporins (Figure 2, 1.96 [95% CI, 1.14–3.35]). However, no experimental evidence has suggested intra-class differences for collagen damage in the eye. One possibility is due to chance findings because many comparisons were conducted. Another explanation is that residual confounding because fluoroquinolone users (especially respiratory fluoroquinolone users) may have more ophthalmological or non-ophthalmological comorbidities, leading to a higher RRD risk (Pasternak et al., 2013; Daneman et al., 2015; Lund et al., 2015). If that impact occurred in our study, we would expect the results to be more toward the null association after a comprehensive adjustment for confounding.

## Conclusion

Our comprehensive, large-scale cohort study which leveraged real-world claims data from Taiwan and the United States showed a low and comparable RRD risk among adults initiating fluoroquinolones or comparator antibiotics with similar indications. Our findings provide reassuring evidence regarding the safety of fluoroquinolones in relation to RRD risk, supporting their use in clinical practice with an awareness of the risk factors and patient characteristics that may influence outcomes.

## Data Availability

The data analyzed in this study are subject to the following licenses/restrictions. The data used in the current study are not publicly available due to the data protection policy declared by the Ministry of Health and Welfare in Taiwan, and restrictions apply to the availability of the US IBM MarketScan Database, which was used under license. Requests about database accessibility should be directed to Yaa-Hui Dong (yaahuidong@gmail.com) and Wei-Hsuan Lo-Ciganic (jenny.lociganic@pitt.edu).
