# Supplementary material for: Use of fluoroquinolones and risk of rhegmatogenous retinal detachment: a retrospective cohort study using two nationwide representative claims databases
Source: Front Pharmacol. 2024 Dec 11;15:1414221. doi: 10.3389/fphar.2024.1414221 (PMC11668581; doi:10.3389/fphar.2024.1414221)
Supplement: Supplementary file 1 [file DataSheet1.docx]

**Online data supplement**

**Use of fluoroquinolones and risk of rhegmatogenous retinal detachment: a retrospective cohort study using two nationwide representative claims databases**

| **DATA SOURCES** | P4 |
| --- | --- |
|  |  |
| **EXCLUSION CRITERIA** | P5 |
|  |  |
| **Table S1.** Specific protocol modifications for individual databases | P7 |
|  |  |
| **Table S2.** Anatomical Therapeutic Chemical (ATC) classification system codes used to identify study antibiotics marketed in Taiwan and in the U.S. | P9 |
|  |  |
| **Table S3**. International Classification of Diseases, 9th or 10th Revision, Clinical Modification (ICD-9-CM or ICD-10-CM) diagnosis or procedure codes, Taiwan health insurance service claims codes, Taiwan medical device codes, or U.S. Current Procedure Terminology (CPT) codes used to exclude patients with retinal detachment or defects, patients receiving retinal detachment-related clinical management, or patients who were non- at-risk of the outcome of interest at baseline | P11 |
|  |  |
| **Table S4**. International Classification of Diseases, 9th or 10th Revision, Clinical Modification (ICD-9-CM or ICD-10-CM) diagnosis or procedure codes, Taiwan health insurance service claims codes, Taiwan medical device codes, or U.S. Current Procedure Terminology (CPT) codes, used to identify the outcome of interest | P13 |
|  |  |
| **Table S5**. International Classification of Diseases, 9th or 10th Revision, Clinical Modification (ICD-9-CM or ICD-10-CM) diagnosis or procedure codes, Taiwan health insurance service claims codes, Taiwan medical device codes, or U.S. Current Procedure Terminology (CPT) codes used to identify potential indications for study antibiotics and comorbidities at baseline | P14 |
|  |  |
| **Table S6**. Anatomical Therapeutic Chemical (ATC) classification system codes used to identify medication use at baseline | P24 |
|  |  |
| **Table S7.** Summary of study cohort assembly in each database | P26 |
|  |  |
| **Table S8.** Distribution of study antibiotics in the eligible cohort in each database and across databases | P28 |
|  |  |
| **Table S9.** Patient characteristics among initiators of fluoroquinolones and initiators of amoxicillin/clavulanate or ampicillin/sulbactam before and after propensity score matching in the Taiwan NHIRD | P30 |
|  |  |
| **Table S10.** Patient characteristics among initiators of fluoroquinolones and initiators of amoxicillin/clavulanate or ampicillin/sulbactam before and after propensity score matching in the U.S. IBM MarketScan Database | P35 |
|  |  |
| **Table S11.** Patient characteristics among initiators of fluoroquinolones and initiators of extended-spectrum cephalosporins before and after propensity score matching in the Taiwan NHIRD | P40 |
|  |  |
| **Table S12.** Patient characteristics among initiators of fluoroquinolones and initiators of extended-spectrum cephalosporins before and after propensity score matching in the U.S. IBM MarketScan Database | P45 |
|  |  |
| **Table S13.** Number of patients and events, follow-up duration, and incidence rate of rhegmatogenous retinal detachment comparing fluoroquinolones to amoxicillin/clavulanate or ampicillin/sulbactam before and after propensity score matching in each database | P50 |
|  |  |
| **Table S14.** Number of patients and events, follow-up duration, and incidence rate of rhegmatogenous retinal detachment comparing fluoroquinolones to extended-spectrum cephalosporins before and after propensity score matching in each database | P51 |
|  |  |
| **Table S15.** Association between fluoroquinolones to amoxicillin/clavulanate ampicillin/sulbactam and rhegmatogenous retinal detachment before and after propensity score matching in each database | P52 |
|  |  |
| **Table S16.** Association between fluoroquinolones to extended-spectrum cephalosporins and rhegmatogenous retinal detachment before and after propensity score matching | P53 |
|  |  |
| **Table S17.** Sensitivity analysis of number of patients and events, incidence rate, and HR of rhegmatogenous retinal detachment comparing fluoroquinolones to amoxicillin/clavulanate or ampicillin/sulbactam before and after propensity score matching across databases, by follow-up duration | P54 |
|  |  |
| **Table S18.** Sensitivity analysis of number of patients and events, incidence rate, and HR of rhegmatogenous retinal detachment comparing fluoroquinolones to extended-spectrum cephalosporins before and after propensity score matching across databases, by type of fluoroquinolone and by follow-up duration | P56 |
|  |  |
| **Table S19.** Subgroup analysis of number of patients and events, incidence rate, and HR of rhegmatogenous retinal detachment comparing fluoroquinolones to amoxicillin/clavulanate or ampicillin/sulbactam before and after propensity score matching across databases, by patient characteristic | P58 |
|  |  |
| **Table S20.** Subgroup analysis of number of patients and events, follow-up duration, incidence rate, and HR of rhegmatogenous retinal detachment comparing fluoroquinolones to extended-spectrum cephalosporins before and after propensity score matching across databases, by patient characteristic | P61 |
|  |  |
| **Table S21.** Subgroup analysis of number of patients and events, incidence rate, and HR of rhegmatogenous retinal detachment comparing fluoroquinolones to amoxicillin/clavulanate or ampicillin/sulbactam before and after propensity score matching across databases, by fluoroquinolone | P64 |
|  |  |
| **Table S22.** Subgroup analysis of number of patients and events, follow-up duration, incidence rate, and HR of rhegmatogenous retinal detachment comparing fluoroquinolones to extended-spectrum cephalosporins before and after propensity score matching across databases, by fluoroquinolone | P67 |
|  |  |
| **Table S23.** Association between fluoroquinolones versus comparison antibiotics and rhegmatogenous retinal detachment before and after propensity score matching in the Taiwan NHIRD, by treatment setting | P70 |
|  |  |
| **Table S24.** Crude incidence rates of retinal detachment between fluoroquinolones users and comparison groups in available cohort studies and our study | P71 |
|  |  |
| **Figure S1.** Summary of available studies examining the association between fluoroquinolones use and retinal detachment (rhegmatogenous type mainly) | P72 |
|  |  |
| **REFERENCES** | P74 |

**DATA SOURCES**

**Taiwan National Health Insurance Research Database (NHIRD)[1,2]**

Taiwan began a single-payer national health insurance program in 1995, which enrolled 99% of the 23-million residents by 2022. The Taiwan National Health Insurance Research Database (NHIRD) is a research data environment which consists of claims data generated from the national health program and is updated annually by the National Health Insurance Administration in Taiwan.

**United States (U.S.) IBM MarketScan Database[3]**

The U.S. IBM MarketScan Database comprises data of more than 200 million beneficiaries from two core databases since 1995, including the IBM MarketScan Commercial Database and the IBM MarketScan Medicare Supplemental Database. The IBM MarketScan Commercial Database includes active employees (as well as their dependents) enrolled in the employer-sponsored private health insurance programs. The IBM MarketScan Medicare Supplemental Database includes Medicare-eligible retirees enrolled in the employer-sponsored Medicare Supplemental programs.

Similar to most health insurance claims databases, the Taiwan NHIRD and the U.S. IBM MarketScan Database contain comprehensive demographic and enrollment records; diagnosis, procedure, and health services codes from outpatient visits, emergency department visits, and hospital admissions; as well as pharmacy dispensing claims from outpatient visits. Of note, the Taiwan NHIRD additionally captures medication use at emergency department visits and during hospitalization, including oral and injectable drug use. The Taiwan NHIRD also can be linked to the National Death Registry and therefore enables to ascertain death information. All the data are anonymized by scrambling the identification codes of patients, healthcare providers, and healthcare facilities.

**EXCLUSION CRITERIA**

From each database, we identified adult patients aged ≥18 years who initiated oral fluoroquinolones, amoxicillin/clavulanate or ampicillin/sulbactam, or extended-spectrum cephalosporins (2^nd^-, 3^rd^-, or 4^th^-generation cephalosporins) at outpatient, emergency department, or inpatient visits in corresponding cohort identification periods (Taiwan NHIRD, 2009/1/1 to 2018/8/31; U.S. IBM MarketScan, 2011/1/1 to 2020/9/30). The cohort entry date was defined as the date of the first dispensing of a study antibiotic.

We applied several exclusion criteria: (1) to ascertain more comprehensive capture of patient characteristics, we required patients to have interactions with the healthcare systems within 180 days before cohort entry; (2) to employ an incident-user design, we required patients who initiated an oral study antibiotic to have no dispensing of any oral or injectable study drugs within 180 days before cohort entry; (3) to reduce confounding by infection severity (patients with more severe infections tend to receive injectable treatment), patients who simultaneously initiated oral and injectable study antibiotics at cohort entry were not included in the analysis; (4) we excluded patients aged <18 years or >100 years at cohort entry and those with unknown or missing age or sex information; (5) we excluded patients who received more than one class of study antibiotic or more than one fluoroquinolone at cohort entry; (6) to reduce confounding by disease history and to prevent including patients who were non- at-risk of the outcome of interest, we also excluded patients who had prior retinal detachment (RD) or retinal defects, who received RD-related clinical management (scleral buckling, cryotherapy, laser therapy, pneumatic retinopexy, and vitrectomy mainly), who were blindness, or who received evisceration or enucleation of eyeball (looking back as early as possible: Taiwan NHIRD, 2008/1/1; U.S. IBM MarketScan, 2010/1/1); and (7) to ensure data quality, we also excluded a very small proportion of patients who had death records before cohort entry (looking back as early as possible: Taiwan NHIRD, 2008/1/1).

**Table S1.** Specific protocol modifications for individual databases

| Condition | Modification in each database |
| --- | --- |
| Study populations and exposure | |
| We identified eligible patients who initiated oral fluoroquinolones, amoxicillin/clavulanate or ampicillin/sulbactam, or extended-spectrum cephalosporins during the study period. | - Taiwanese data: information on oral study antibiotics was captured from outpatient, emergency department, and inpatient visits - U.S. data: information on oral study antibiotics was only ascertained from outpatient visits. |
| To ascertain comprehensive capture of patient characteristics, we required patients to have interactions with the healthcare systems within 180 days before cohort entry. | - Taiwanese data: interactions with the healthcare system were defined as having at least one outpatient, emergency department, or inpatient visits during the 180-day window. - U.S. data: interactions with the healthcare system were operationally defined as having no insurance coverage gap during the baseline window. |
| To employ an incident-user design | - Taiwanese data: we required patients who initiated an oral study antibiotic to have no dispensing of any oral or injectable study drugs from outpatient, emergency department, and inpatient visits during the baseline window. - U.S. data: we only required patients without any oral study drugs (regardless of injectable drug information) from outpatient visits within the baseline window given lack of information from emergency department and inpatient visits which was important for distinguishing injectable study antibiotic use. |
| To reduce confounding by infection severity | - Taiwanese data: patients who simultaneously initiated oral and injectable study antibiotics at cohort entry were not included in the analysis. - U.S. data: not applicable (same reason addressed above). |
| To ensure data quality | - Taiwanese data: we excluded a very small proportion of patients who had death records before cohort entry - U.S. data: not applicable given lack of complete death information. |
|  |  |
|  | Follow-up |
| Censor criteria | - Taiwanese data: patients were followed for up to 90 days from cohort entry to the earliest of outcome occurrence, death, or end of data. We did not need a disenrollment censor criterion given the single-payer, compulsory health insurance system. - U.S. data: patients were followed for up to 90 days from cohort entry to the earliest of outcome occurrence, disenrollment, or end of data. A disenrollment criterion was required given that patients may change jobs and health insurance plans. The death information was not available and therefore not applicable. |
|  |  |
| Covariates | |
| Hospital accreditation levels | - Taiwanese data: available and applicable (medical centers, regional hospitals, district hospitals, and primary care clinics). - U.S. data: not available and not applicable. |
| Medication use | - Taiwanese data: information on medication use was captured from outpatient, emergency department, and inpatient claims. - U.S. data: information on medication use was only available from outpatient claims. |

U.S. United States.

**Table S2.** Anatomical Therapeutic Chemical (ATC) classification system codes used to identify study antibiotics marketed in Taiwan and in the U.S.^a,b^

| Study antibiotics | ATC classification system codes | Dosage forms^c^ | Taiwan available | U.S. available |
| --- | --- | --- | --- | --- |
| Fluoroquinolones | | | | |
| Respiratory fluoroquinolones | | |  |  |
| Levofloxacin | J01MA12 | oral, injectable | V | V |
| Moxifloxacin | J01MA14 | oral, injectable | V | V |
| Gemifloxacin | J01MA15 | oral, injectable | V | V |
| Non-respiratory fluoroquinolones | | |  |  |
| Ofloxacin | J01MA01 | oral, injectable | V | V |
| Ciprofloxacin | J01MA02 | oral, injectable | V | V |
| Pefloxacin | J01MA03 | oral, injectable | V |  |
| Enoxacin | J01MA04 | oral | V | V |
| Norfloxacin | J01MA06 | oral | V | V |
| Lomefloxacin | J01MA07 | oral | V | V |
| Sparfloxacin | J01MA09 | oral | V | V |
| Grepafloxacin | J01MA11 | oral |  | V |
| Trovafloxacin | J01MA13 | oral, injectable |  | V (oral only) |
| Delafloxacin | J01MA23 | oral, injectable |  | V |
|  |  |  |  |  |
| Amoxicillin/ampicillin and beta-lactamase inhibitors | | | | |
| Amoxicillin and beta-lactamase Inhibitor | J01CR02 | oral, injectable | V | V (oral only) |
| Sultamicillin | J01CR04 | oral | V |  |
|  |  |  |  |  |
| Extended-spectrum cephalosporins | | | | |
| 2^nd^-generation cephalosporins | | | | |
| Cefoxitin | J01DC01 | injectable | V | V |
| Cefuroxime | J01DC02 | oral, injectable | V | V |
| Cefamandole | J01DC03 | injectable | V | V |
| Cefaclor | J01DC04 | oral | V | V |
| Cefotetan | J01DC05 | injectable | V | V |
| Cefonicid | J01DC06 | injectable | V | V |
| Cefotiam | J01DC07 | oral, injectable | V (injection only) | V (injection only) |
| Loracarbef | J01DC08 | oral |  | V |
| Cefmetazole | J01DC09 | injectable | V | V |
| Cefprozil | J01DC10 | oral |  | V |
| Ceforanide | J01DC11 | injectable |  | V |
| Flomoxef | J01DC14 | injectable | V |  |
| 3^rd^-generation cephalosporins | | | | |
| Cefotaxime | J01DD01 | injectable | V | V |
| Ceftazidime | J01DD02 | injectable | V | V |
| Ceftriaxone | J01DD04 | injectable | V | V |
| Cefmenoxime | J01DD05 | injectable | V | V |
| Latamoxef | J01DD06 | injectable | V |  |
| Ceftizoxime | J01DD07 | injectable | V | V |
| Cefixime | J01DD08 | oral | V | V |
| Cefpiramide | J01DD11 | injectable |  | V |
| Cefoperazone | J01DD12 | injectable | V | V |
| Cefpodoxime | J01DD13 | oral | V | V |
| Ceftibuten | J01DD14 | oral | V | V |
| Cefdinir | J01DD15 | oral |  | V |
| Cefditoren | J01DD16 | oral |  | V |
| Ceftazidime and beta-lactamase Inhibitor | J01DD52 | injectable | V | V |
| Cefoperazone and beta-lactamase Inhibitor | J01DD62 | injectable | V |  |
| 4^th^-generation cephalosporins | | | | |
| Cefepime | J01DE01 | injectable | V | V |
| Cefpirome | J01DE02 | injectable | V |  |
| Cefozopran | J01DE03 | injectable |  |  |

U.S. United States.

^a^Only study antibiotics that were marketed in Taiwan (2008-2018) and in the U.S. (2010-2020) were listed and were included in the analysis.

^b^Based on outpatient visit, emergency department visit (Taiwanese data only), and inpatient (Taiwanese data only) pharmacy dispensing claims.

^c^Patients who simultaneously initiated oral and injectable study antibiotics at cohort entry in the Taiwanese database were not included in the analysis.

**Table S3**. International Classification of Diseases, 9th or 10th Revision, Clinical Modification (ICD-9-CM or ICD-10-CM) diagnosis or procedure codes, Taiwan health insurance service claims codes,^a^ Taiwan medical device codes,^a^ or U.S. Current Procedure Terminology (CPT) codes^b^ used to exclude patients with retinal detachment or defects, patients receiving retinal detachment-related clinical management, or patients who were non- at-risk of the outcome of interest at baseline

| Variable description | Variable definition |
| --- | --- |
| Retinal detachments or defects | ICD-9-CM diagnosis codes: 361  ICD-10-CM diagnosis codes: H33 |
| Retinal detachment-related clinical management (mainly including scleral buckling, cryotherapy, laser therapy, pneumatic retinopexy, and vitrectomy) | ICD-9 proc codes: 14.3, 14.4, 14.5, 14.7  ICD-10 proc codes: 08QE3ZZ, 08QF3ZZ, 08UE07Z, 08UE0KZ, 08UE37Z, 08UE3KZ, 08UF07Z, 08UF0KZ, 08UF37Z, 08UF3KZ, 08UE0JZ, 08UE3JZ, 08UF0JZ, 08UF3JZ, 08B43ZZ, 08B53ZZ, 08B6XZZ, 08B7XZZ, 3E0CXSF, 08QE3ZZ,08QF3ZZ,3E0C3GC, 08R437Z, 08R43JZ, 08R43KZ, 08R537Z, 08R53JZ, 08R53KZ, 089430Z, 08943ZZ, 089530Z, 08953ZZ, 08B43ZZ, 08B53ZZ, 08T43ZZ, 08T53ZZ, 3E0C3GC, 08543ZZ, 08553ZZ, 08F43ZZ, 08F53ZZ, 08Q43ZZ, 08Q53ZZ  Taiwan health insurance service claims codes: 85608B, 85608BA, 86406B, 85609B, 85610B, 85611B, 86211B, 86411B,86413B, 85608BA, 86402B, 86402C, 86404B, 86411B, 60003C, 60004C, 60005C, 60006C, 86201CA, 86215C, 86203B, 86203C, 86206B, 86206C, 86207B, 86208B, 86208C, 86209B, 86209C, 86409B, 86410B, 86411B, 86413B, 86414B, 86415B, 23809C, 86403B  Taiwan medical device codes: TBS01510101Z, TBS01523101Z, TBS01S51001Z, CXE0360SF7M4, CXE03CRY02FW, CXE03CRY03FW, CXE03CRYCSFW TBS01S52301Z, TBS01S56301Z, FAV03SL12549, FAV03SL1258A, FAV03SLRS77A, FAV03SLS571Z, FAV03SLSS109, TKVC100VC1LG, TKVC105064QQ, TKVC10506NQQ, TKVC105100MV, TKVC105124QQ, TKVC12405CZS, TKVC141018A1, TKVC1CX408B9, TKVC244006B9, TKVC256120B9, TKVC256230B9, TKVC2NGP00A2, TKVS1D4801B9, TKVS1D4803B9, TKVS1D4803SZ, TKVS1SUPRALA, TKF01C9606B9, TKF01DP602B9  U.S. CPT codes: 67107, 67112, 67101, 67105, 67110, 67108, 67112, 67113, 67141, 67145 |
| Blindness | ICD-9-CM diagnosis codes: 369.XX, V19.0X  ICD-10-CM diagnosis codes: H54.XX, Z82.1X |
| Evisceration of eyeball | ICD-9-CM procedure codes: 16.3X  ICD-10-CM procedure codes: 08R00JZ, 08R10JZ, 08T0XZZ, 08T1XZZ  Taiwan health insurance service claims codes: 85002C  U.S. CPT codes: 65091, 65093 |
| Enucleation of eyeball | ICD-9 proc codes: 16.4X  ICD-10 proc codes: 08R00JZ, 08R10JZ, 08R007Z, 08R00KZ, 08R037Z, 08R03JZ, 08R03KZ, 08R107Z, 08R10KZ, 08R137Z, 08R13JZ, 08R13KZ, 08T0XZZ, 08T1XZZ  Taiwan health insurance service claims codes: 85001C  U.S. CPT codes: 65101, 65103, 65105 |

U.S., United States.

^a^Only applied in the Taiwanese data.

^b^Only applied in the U.S. data.

**Table S4**. International Classification of Diseases, 9th or 10th Revision, Clinical Modification (ICD-9-CM or ICD-10-CM) diagnosis or procedure codes, Taiwan health insurance service claims codes,^a^ Taiwan medical device codes,^a^ or U.S. Current Procedure Terminology (CPT) codes,^b^ used to identify the outcome of interest

| Variable description | Variable definition |
| --- | --- |
| Rhegmatogenous retinal detachment | ICD-9-CM diagnosis codes: 361.0 (except 361.06 and 361.07)  ICD-10-CM diagnosis codes: H33.0 |
| Retinal detachment-related clinical management (mainly including scleral buckling, cryotherapy, laser therapy, pneumatic retinopexy, and vitrectomy) | Same as what was shown in Table S3. |

U.S., United States.

^a^Only applied in the Taiwanese data.

^b^Only applied in the U.S. data.

**Table S5**. International Classification of Diseases, 9th or 10th Revision, Clinical Modification (ICD-9-CM or ICD-10-CM) diagnosis or procedure codes, Taiwan health insurance service claims codes,^a^ Taiwan medical device codes,^a^ or U.S. Current Procedure Terminology (CPT) codes^b^ used to identify potential indications for study antibiotics and comorbidities at baseline

| Variable description | Variable definition |
| --- | --- |
| Acute infection that served as potential indications for study antibiotics | |
| Lower respiratory tract infections^c^ | ICD-9-CM diagnosis codes: 480-483, 485-487, 510, 513  ICD-10-CM diagnosis codes: J09-J16, J18 (except J18.2), J85, J86, A48.1 |
| Reproductive and urinary tract infections | ICD-9-CM diagnosis codes: 590, 595.0, 595.9, 597.0, 597.8, 599.0, 601, 604, 614-616  ICD-10-CM diagnosis codes: N10, N11.0, N13.6, N15.1, N30, N34, N39.0, N41, N45, N70- N73, N75.1, N76, N77.1 |
| Skin, soft tissue, and bone infections^d^ | ICD-9-CM diagnosis codes: 680-686, 711.0, 730, 728.86  ICD-10-CM diagnosis codes: B78.1, E83.2, L01-L05, L08, L88, L92.8, L98.0, L98.3, M00, M86, M89.6, M90.8, M462, M463, M72.6 |
| Intra-abdominal infections | ICD-9-CM diagnosis codes: 540-542, 562.01, 562.03, 562.11, 562.13, 566, 567, 569.5, 572.0, 572.1, 575.0  ICD-10-CM diagnosis codes: K35-K37, K57.00, K57.01, K57.12, K57.13, K57.20, K57.21, K57.32, K57.33, K57.40, K57.52, K57.80, K57.81, K57.92, K57.93, K61, K63.0, K65, K67, K68, K75.0, K75.1, K81.0 |
| Septicemia | ICD-9-CM diagnosis codes: 038, 041.9, 790.7, 785.52  ICD-10-CM diagnosis codes: A40, A41, A49.9, B96.89, R65, R78.81 |
| Acute respiratory tract infections^e^ | ICD-9-CM diagnosis codes: 460, 461, 462, 463, 464, 465, 466  ICD-10-CM diagnosis codes: J00, J01, J02.8, J02.9, J03.8, J03.9, J04, J05, J06, J20, J21 |
|  | |
| Ophthalmic comorbidities | |
| Endophthalmitis | ICD-9-CM diagnosis codes: 360.0, 360.1  ICD-10-CM diagnosis codes: H16.24, H21.33, H33.12, H44.0, H44.1, H45.1 |
| Intra-ocular foreign body | ICD-9-CM diagnosis codes: 360.5, 360.6  ICD-10-CM diagnosis codes: H44.6, H44.7 |
| Myopia | ICD-9-CM diagnosis codes: 360.21, 367.1  ICD-10-CM diagnosis codes: H44.2, H52.1 |
| Diabetic retinopathy | ICD-9-CM diagnosis codes: 362.0  ICD-10-CM diagnosis codes: E11.31-E11.35 |
| Retinal vascular occlusion | ICD-9-CM diagnosis codes: 362.3  ICD-10-CM diagnosis codes: H34, G45.3 |
| Other retinal disorders | ICD-9-CM diagnosis codes: 362.1, 362.2, 362.4-362.9  ICD-10-CM diagnosis codes: H35, H36 |
| Choroioretinitis | ICD-9-CM diagnosis codes: 363.0-363.2  ICD-10-CM diagnosis codes: H30, H32 |
| Glaucoma | ICD-9-CM diagnosis codes: 365  ICD-10-CM diagnosis codes: H40, H42  Taiwan health insurance service claims codes: 23304C, 23808B, 60007C, 60008C, 60011C, 60012C, 85601C, 85806CA, 85810CA, 85823B  Taiwan medical device codes: FAD01000S2ZJ, FAD013422652, FAD0150201UX, FAD01K120951  U.S. CPT codes: 65820, 65850, 65855, 65865, 66150, 66155, 66161, 66170, 66172, 66174, 66179, 66180, 66184, 66185, 66186, 66500, 66505, 66600, 66605, 66625, 66630, 66635, 66761, 66762, 0191T, 0449T, 65820, 66170, 66174, 66761, 66840, 66940, 66982, 66984, 66987, 66988 |
| Cataract | ICD-9-CM diagnosis codes: 366  ICD-10-CM diagnosis codes: H25, H26, H28, E11.36  ICD-9-CM procedure codes: 13 (excluded 13.0)  ICD-10-CM procedure codes: 08DJ3ZZ, 08DK3ZZ, 085J3ZZ, 085K3ZZ, 08BJ3ZZ, 08BK3ZZ, 08RJ3JZ, 08RK3JZ, 08QJ3ZZ, 08QK3ZZ, 08RJ3J5, 08RK3J5, 83017B1, 86003CA, 86001C, 86002C, 86003C, 86004C, 86005C, 86006C, 86007C, 86008C, 86009C, 86010A, 86010B, 86011C, 86012C, 86013C, 97601K, 97602A, 97603B, 97605K, 97606A, 97607B, 97608C  Taiwan medical device codes: TKVC256230B9, FALSM050966M, FALSM3LENSHB, FALSM574R1RY, FALSM678946M, FALSMAAB00A2, FALSMACRA11X, FALSMAR40NA2, FALSMAVE10HY, FALSMCR5BUA1, FALSMCSFLXRY, FALSMCT2027Z, FALSMGAB00JJ, FALSMHBEC11B, FALSMHBEC11E, FALSMHBEC21E, FALSMMZ30BA1, FALSMNEFL1RQ, FALSMNERP1RQ, FALSMRA100RY, FALSMSA60AA1, FALSMSL0004H, FALSMT872S41, FALSMUF6005K, FALSN33798A1, FALSN33799A1, FALSN34093A1, FALSN35005A1, FALSN35049A1, FALSNAAXACPJ, FALSNASPPC6C, FALSNAT7097Z, FALSNAT8297Z, FALSNAT9297Z, FALSNAT9497Z, FALSNAVE11HY, FALSNAVE12HY, FALSNAVE13HY, FALSNAVE14HY, FALSNCRYA0B9, FALSNCRYHDB9, FALSNCT4097Z, FALSNDCB00JJ, FALSNED60C6C, FALSNED60Y6C, FALSNEDEN1S1, FALSNHPES04H, FALSNMERV1A2, FALSNMETC221, FALSNMF60C6C, FALSNMF60Y6C, FALSNMFMT16Z, FALSNMLTT24L, FALSNMLTT34L, FALSNMULF17P, FALSNMULT0A1, FALSNMULT14L, FALSNMULT15K, FALSNMULT15V, FALSNMULT17P, FALSNMULT1A1, FALSNMULT1A2, FALSNMULT1LS, FALSNMULT1RY, FALSNMULT221, FALSNMULT24L, FALSNMULT25K, FALSNMULT25V, FALSNMULT2A1, FALSNMULT2A2, FALSNMULT2LS, FALSNMULT2RY, FALSNMULT35K, FALSNMULT35V, FALSNMULT3A1, FALSNMULT3A2, FALSNMULT3RY, FALSNMULT45K, FALSNMULT45V, FALSNMULT4A1, FALSNMULT4A2, FALSNMULT4RY, FALSNMULT55V, FALSNMULT5A2, FALSNMULT65V, FALSNMULT6A2, FALSNMULTT4L, FALSNMULTT8V, FALSNMULTTA1, FALSNMULTVA1, FALSNNATU1A1, FALSNNATU2A1, FALSNNS60Y4N, FALSNPED6C6C, FALSNPLGF17P, FALSNPLSZ14N, FALSNPMF6C6C, FALSNPMSAC1W, FALSNPMSAY1W, FALSNPRE5T6P, FALSNPRE6M6P, FALSNPTF6C6C, FALSNPTF6Y6C, FALSNPTR6C6C, FALSNPTR6Y6C, FALSNPTSAC1W, FALSNPTSAY1W, FALSNSERV17P, FALSNSERV1A2, FALSNSERV2A2, FALSNSERV3A2, FALSNSERVTA2, FALSNSLAF04H, FALSNSLAFE4H, FALSNSLHP04H, FALSNSLMF04H, FALSNT0RC0A1, FALSNT0RC11W, FALSNT0RC15V, FALSNT0RC17P, FALSNT0RC1A1, FALSNT0RC1A2, FALSNT0RC1B9, FALSNT0RC1RY, FALSNT0RC2A1, FALSNT0RC2A2, FALSNT0RC2RY, FALSNT0RC3RY, FALSNT0RCA4L, FALSNT0RCARY, FALSNT0RCY1W, FALSNT0RTU1D, FALSNT0RTY1D, FALSNT1R1C10, FALSNT2ZCUJJ, FALSNTF60C6C, FALSNTF60Y6C, FALSNTR60C6C, FALSNTR60Y6C, FALSNTRFCLRY, FALSNTRFT2RY, FALSNVSTR04H, FALSNWAVE11D, FALSNWAVE11E, FALSNWAVE11K, FALSNWAVE11W, FALSNWAVE11X, FALSNWAVE121, FALSNWAVE13Q, FALSNWAVE15V, FALSNWAVE16C, FALSNWAVE17P, FALSNWAVE1A1, FALSNWAVE1A2, FALSNWAVE1B9, FALSNWAVE1HY, FALSNWAVE1LS, FALSNWAVE1PJ, FALSNWAVE1RQ, FALSNWAVE1RY, FALSNWAVE1S2, FALSNWAVE21D, FALSNWAVE21E, FALSNWAVE21X, FALSNWAVE221, FALSNWAVE25V, FALSNWAVE2A1, FALSNWAVE2A2, FALSNWAVE2B9, FALSNWAVE2HY, FALSNWAVE2LS, FALSNWAVE2RY, FALSNWAVE35V, FALSNWAVE3A1, FALSNWAVE3A2, FALSNWAVE3B9, FALSNWAVE3HY, FALSNWAVE3LS, FALSNWAVE3RY, FALSNWAVE45V, FALSNWAVE4A1, FALSNWAVE4A2, FALSNWAVE4B9, FALSNWAVE4HY, FALSNWAVE4LS, FALSNWAVE4RY, FALSNWAVE55V, FALSNWAVE56Z, FALSNWAVE5A2, FALSNWAVE5B9, FALSNWAVE5HY, FALSNWAVE5RY, FALSNWAVE65V, FALSNWAVE6A2, FALSNWAVE6B9, FALSNWAVE6HY, FALSNWAVE6RY, FALSNWAVE7A2, FALSNWAVE7B9, FALSNWAVE7HY, FALSNWAVE85K, FALSNWAVE8HY, FALSNWAVE9HY, FALSNWAVEPA1, FALSNWAVEY6C, FALSNZFRV1JJ, TKF01B5X20B9, FAV01055CCBH, FAV01085CCBH, FAV01100CCSZ, FAV01C0100A1, FAV01H0506A2, FAV01H0506P6, FAV01HGV85A2, FAV01HGV85P6, FAV01PA001LB, FAV01PA002LB, FAV01PA003LB, FAV01VSC55V3, FAV01VSC85V3, FAV01VT585A2, FAV01AP050B9, FAV01AP050C0, FAV01AP080B9, FAV01AP080C0, FAV01AV050B9, FAV01AV080B9, FAV01AV050C0, FAV01AV080C0, FAV01BC1001X, FAV01BV0553J, FAV01BV1003J, FAV01C0090R9, FAV01C0100R9, FAV01C0150R9, FAV01C0200R9, FAV01C0300R9, FAV01C0400R9, FAV01C0500R9, FAV01D0100A1, FAV01E01003H, FAV01PH1809D, FAV01PH3009D, FAV01RN085RY  U.S. CPT codes: 66820, 66821, 66830, 66982, 66983, 66984, 66985, 66986, 66987, 66988, 66989, 66991 |
| Disorder of orbit | ICD-9-CM diagnosis codes: 376  ICD-10-CM diagnosis codes: H05 |
| Disorders of vitreous body | ICD-9-CM diagnosis codes: 379.2  ICD-10-CM diagnosis codes: H43 |
| Severe head trauma^f^ | ICD-9-CM diagnosis codes: 801, 802.6- 802.9, 803, 804  ICD-10-CM diagnosis codes: S02.1, S02.3, S02.8, S02.9 |
| Severe eye trauma^g^ | ICD-9-CM diagnosis codes: 870.3, 870.4, 871, 921.2, 921.3, 921.9, 940.5, 941.52  ICD-10-CM diagnosis codes: S05.1-S05.9, T26.2, T26.7 |
| Cytomegalovirus diseases  (risk factors of cytomegalovirus retinitis) | ICD-9-CM diagnosis codes: 078.5  ICD-10-CM diagnosis codes: B25.8, B25.9 |
| Acquired immunodeficiency syndrome  (risk factors of cytomegalovirus retinitis) | ICD-9-CM diagnosis codes: 042  ICD-10-CM diagnosis codes: B20 |
| Intravitreal injection | Taiwan health insurance service claims codes: 86201C  U.S. CPT codes: 67028 |
| Choroidal, retinal, or vitreal biopsy | ICD-9-CM procedure codes: 14.1  ICD-10-CM procedure codes: 08943ZX, 08953ZX, 089A0ZX, 089A3ZX, 089B0ZX, 089B3ZX, 089E3ZX, 089F3ZX, 089G3ZX, 089H3ZX, 08B43ZX, 08B53ZX, 08BA0ZX, 08BA3ZX, 08BB0ZX, 08BB3ZX, 08BE3ZX, 08BF3ZX  Taiwan health insurance service claims codes: 86210B, 86212B  U.S. CPT codes: 67015 |
| Removal of lesion of retina and choroid | ICD-9-CM procedure codes: 14.2, 14.9  ICD-10-CM procedure codes: 08H031Z, 08H0X1Z, 08H131Z, 08H1X1Z, 085E3ZZ, 085F3ZZ, 08BE3ZZ, 08BF3ZZ, 085G3ZZ, 085H3ZZ, 089A00Z, 089A0ZZ, 089A30Z, 089A3ZZ, 089B00Z, 089B0ZZ, 089B30Z, 089B3ZZ, 089E30Z, 089E3ZZ, 089F30Z, 089F3ZZ, 089G30Z, 089G3ZZ, 089H30Z, 089H3ZZ, 08CG3ZZ, 08CH3ZZ, 08NA0ZZ, 08NA3ZZ, 08NB0ZZ, 08NB3ZZ, 08NE3ZZ, 08NF3ZZ, 08NG3ZZ, 08NH3ZZ, 08QA0ZZ, 08QA3ZZ, 08QB0ZZ, 08QB3ZZ, 08QE3ZZ, 08QF3ZZ, 08QG3ZZ, 08QH3ZZ, 08RG37Z, 08RG3JZ, 08RG3KZ, 08RH37Z, 08RH3JZ, 08RH3KZ, 08SG3ZZ, 08SH3ZZ, 08UG07Z, 08UG0JZ, 08UG0KZ, 08UG37Z, 08UG3JZ, 08UG3KZ, 08UH07Z, 08UH0JZ, 08UH0KZ, 08UH37Z, 08UH3JZ, 08UH3KZ |
| Other major eye surgery^h^ | ICD-9-CM procedure codes: 11, 12, 15, 16  ICD-10-CM procedure codes: 08C8XZZ, 08C9XZZ, 0898X0Z, 0898XZZ, 0899X0Z, 0899XZZ, 08D8XZX, 08D8XZZ, 08D9XZX, 08D9XZZ, 0898XZX, 0899XZX, 08B8XZX, 08B9XZX, 08J0XZZ, 08J1XZZ, 08QSXZZ, 08QTXZZ, 08R8X7Z, 08R8XKZ, 08R9X7Z, 08R9XKZ, 08U8X7Z, 08U8XKZ, 08U9X7Z, 08U9XKZ, 08BSXZZ, 08BTXZZ, 0858XZZ, 0859XZZ, 08B8XZZ, 08B9XZZ, 08Q8XZZ, 08Q9XZZ, 08U807Z, 08U837Z, 08U907Z, 08U937Z, 08N8XZZ, 08N9XZZ, 08R83KZ, 08R93KZ, 08R837Z, 08R937Z, 08U80KZ, 08U83KZ, 08U90KZ, 08U93KZ, 08R83JZ, 08R8XJZ, 08R93JZ, 08R9XJZ, 08U80JZ, 08U83JZ, 08U8XJZ, 08U90JZ, 08U93JZ, 08U9XJZ, 3E0C3MZ, 3E0C7MZ, 3E0CXMZ, 08P0XJZ, 08P1XJZ, 08T8XZZ, 08T9XZZ, 08C23ZZ, 08C33ZZ, 08QC3ZZ, 08QD3ZZ, 089C30Z, 089C3ZZ, 089D30Z, 089D3ZZ, 08CC3ZZ, 08CD3ZZ, 08BC3ZZ, 08BD3ZZ, 08TC3ZZ, 08TD3ZZ, 08923ZX, 08933ZX, 089C3ZX, 089D3ZX, 08BC3ZX, 08BD3ZX, 0896XZX, 0897XZX, 08B6XZX, 08B7XZX, 08NC3ZZ, 08ND3ZZ, 08N23ZZ, 08N33ZZ, 08NJ3ZZ, 08NK3ZZ, 08N43ZZ, 08N53ZZ, 08RC37Z, 08RC3JZ, 08RC3KZ, 08RD37Z, 08RD3JZ, 08RD3KZ, 08SC3ZZ, 08SD3ZZ, 08UC07Z, 08UC0JZ, 08UC0KZ, 08UC37Z, 08UC3JZ, 08UC3KZ, 08UD07Z, 08UD0JZ, 08UD0KZ, 08UD37Z, 08UD3JZ, 08UD3KZ, 08523ZZ, 08533ZZ, 085C3ZZ, 085D3ZZ, 085A0ZZ, 085A3ZZ, 085B0ZZ, 085B3ZZ, 08BA0ZZ, 08BA3ZZ, 08BB0ZZ, 08BB3ZZ, 08923ZZ, 08933ZZ, 089230Z, 089330Z, 08Q23ZZ, 08Q33ZZ, 08B6XZZ, 08B7XZZ, 08123Z4, 08133Z4, 08123J4, 08123K4, 08133J4, 08133K4, 08Q6XZZ, 08Q7XZZ, 0856XZZ, 0857XZZ, 08R6X7Z, 08R6XJZ, 08R6XKZ, 08R7X7Z, 08R7XJZ, 08R7XKZ, 0896X0Z, 0896XZZ, 0897X0Z, 0897XZZ, 08N6XZZ, 08N7XZZ, 3E0C33Z, 3E0C3BZ, 3E0C3GC, 3E0C3HZ, 3E0C3KZ, 3E0C3SF, 3E0C3TZ, 08QA0ZZ, 08QA3ZZ, 08QB0ZZ, 08QB3ZZ, 08RA07Z, 08RA0JZ, 08RA0KZ, 08RA37Z, 08RA3JZ, 08RA3KZ, 08RB07Z, 08RB0JZ, 08RB0KZ, 08RB37Z, 08RB3JZ, 08RB3KZ, 089L0ZX, 089L3ZX, 089M0ZX, 089M3ZX, 08BL0ZX, 08BL3ZX, 08BM0ZX, 08BM3ZX, 08JL0ZZ, 08JLXZZ, 08JM0ZZ, 08JMXZZ, 08SL0ZZ, 08SL3ZZ, 08SM0ZZ, 08SM3ZZ, 08XL0ZZ, 08XL3ZZ, 08XM0ZZ, 08XM3ZZ, 08TL0ZZ, 08TL3ZZ, 08TM0ZZ, 08TM3ZZ, 08QL0ZZ, 08QL3ZZ, 08QM0ZZ, 08QM3ZZ, 08BL0ZZ, 08BL3ZZ, 08BM0ZZ, 08BM3ZZ, 08NL0ZZ, 08NL3ZZ, 08NM0ZZ, 08NM3ZZ, 085L0ZZ, 085L3ZZ, 085M0ZZ, 085M3ZZ, 089L00Z, 089L0ZZ, 089L30Z, 089L3ZZ, 089M00Z, 089M0ZZ, 089M30Z, 089M3ZZ, 08CL0ZZ, 08CL3ZZ, 08CM0ZZ, 08CM3ZZ, 08PL00Z, 08PL30Z, 08PM00Z, 08PM30Z, 08UL07Z, 08UL0JZ, 08UL0KZ, 08UL37Z, 08UL3JZ, 08UL3KZ, 08UM07Z, 08UM0JZ, 08UM0KZ, 08UM37Z, 08UM3JZ, 08UM3KZ, 08WL00Z, 08WL30Z, 08WM00Z, 08WM30Z, 0NRP07Z, 0NRP37Z, 0NRP47Z, 0NRQ07Z, 0NRQ37Z, 0NRQ47Z, 0NRP0JZ, 0NRP3JZ, 0NRP4JZ, 0NRQ0JZ, 0NRQ3JZ, 0NRQ4JZ, 0NUP0JZ, 0NUP3JZ, 0NUP4JZ, 0NUQ0JZ, 0NUQ3JZ, 0NUQ4JZ, 0J810ZZ, 0J813ZZ, 0N8P0ZZ, 0N8P3ZZ, 0N8P4ZZ, 0N8Q0ZZ, 0N8Q3ZZ, 0N8Q4ZZ, 0N9P00Z, 0N9P0ZZ, 0N9P30Z, 0N9P40Z, 0N9P4ZZ, 0N9Q00Z, 0N9Q0ZZ, 0N9Q30Z, 0N9Q40Z, 0N9Q4ZZ, 08C0XZZ, 08C1XZZ, 0N9P3ZX, 0N9Q3ZX, 08B00ZX, 08B03ZX, 08B0XZX, 08B10ZX, 08B13ZX, 08B1XZX, 0NBP0ZX, 0NBP3ZX, 0NBP4ZX, 0NBQ0ZX, 0NBQ3ZX, 0NBQ4ZX, 0890XZX, 0891XZX, 0N9P0ZX, 0N9P4ZX, 0N9Q0ZX, 0N9Q4ZX, 08R00JZ, 08R10JZ, 08T0XZZ, 08T1XZZ, 08R007Z, 08R00KZ, 08R037Z, 08R03JZ, 08R03KZ, 08R107Z, 08R10KZ, 08R137Z, 08R13JZ, 08R13KZ, 0NBR0ZZ, 0NBR4ZZ, 0NBP0ZZ, 0NBP4ZZ, 0NBS0ZZ, 0NBS4ZZ, 0NBQ0ZZ, 0NBQ4ZZ, 0NBP3ZZ, 0NBQ3ZZ, 0KX10ZZ, 0KX14ZZ, 08W00JZ, 08W03JZ, 08W10JZ, 08W13JZ, 0JR107Z, 0JR10KZ, 0JR137Z, 0JR13KZ, 0WQ20ZZ, 0WQ23ZZ, 0WQ24ZZ, 0WQ2XZZ, 08P003Z, 08P00JZ, 08P103Z, 08P10JZ, 0NPW0JZ, 0NPW3JZ, 0NPW4JZ, 0NQP0ZZ, 0NQP3ZZ, 0NQP4ZZ, 0NQPXZZ, 0NQQ0ZZ, 0NQQ3ZZ, 0NQQ4ZZ, 0NQQXZZ, 08Q0XZZ, 08Q1XZZ, 3E0C329, 08B00ZZ, 08B03ZZ, 08B0XZZ, 08B10ZZ, 08B13ZZ, 08B1XZZ, 0850XZZ, 0851XZZ, 0890X0Z, 0890XZZ, 0891X0Z, 0891XZZ, 08H033Z, 08H0X3Z, 08H133Z, 08H1X3Z, 08N0XZZ, 08N1XZZ, 08P000Z, 08P001Z, 08P007Z, 08P00KZ, 08P030Z, 08P031Z, 08P033Z, 08P037Z, 08P03JZ, 08P03KZ, 08P071Z, 08P073Z, 08P077Z, 08P07KZ, 08P081Z,08P083Z, 08P087Z, 08P08KZ, 08P0X1Z,08P0X7Z, 08P0XKZ, 08P100Z, 08P101Z, 08P107Z, 08P10KZ,08P130Z, 08P131Z, 08P133Z, 08P137Z, 08P13JZ, 08P13KZ, 08P171Z, 08P173Z, 08P177Z, 08P17KZ, 08P181Z, 08P183Z, 08P187Z, 08P18KZ, 08P1X7Z, 08P1XKZ, 08PL07Z, 08PL0JZ, 08PL0KZ, 08PL37Z, 08PL3JZ, 08PL3KZ, 08PM07Z, 08PM0JZ, 08PM0KZ, 08PM37Z, 08PM3JZ, 08PM3KZ, 08W000Z, 08W003Z, 08W007Z, 08W00CZ, 08W00DZ, 08W00KZ, 08W030Z, 08W033Z, 08W037Z, 08W03CZ, 08W03DZ, 08W03KZ, 08W070Z, 08W073Z, 08W077Z, 08W07CZ, 08W07DZ, 08W07JZ, 08W07KZ, 08W080Z, 08W083Z, 08W087Z, 08W08CZ, 08W08DZ, 08W08JZ, 08W08KZ, 08W100Z, 08W103Z, 08W107Z, 08W10CZ, 08W10DZ, 08W10KZ, 08W130Z, 08W133Z, 08W137Z, 08W13CZ, 08W13DZ, 08W13KZ, 08W170Z, 08W173Z, 08W177Z, 08W17CZ, 08W17DZ, 08W17JZ, 08W17KZ, 08W180Z, 08W183Z, 08W187Z, 08W18CZ, 08W18DZ, 08W18JZ, 08W18KZ,08WL07Z,08WL0JZ,08WL0KZ, 08WL37Z, 08WL3JZ, 08WL3KZ, 08WM07Z, 08WM0JZ, 08WM0KZ, 08WM37Z, 08WM3JZ, 08WM3KZ |
|  | |
| Non-ophthalmic comorbidities | |
| Hypertension | ICD-9-CM diagnosis codes: 401-405  ICD-10-CM diagnosis codes: I10-I15 |
| Ischemic heart disease | ICD-9-CM diagnosis codes: 411, 413, 414  ICD-10-CM diagnosis codes: I20, I24, I25 |
| Myocardial infarction | ICD-9-CM diagnosis codes: 410, 412  ICD-10-CM diagnosis codes: I21, I22, I25.2 |
| Coronary artery bypass grafting | ICD-9-CM procedure codes: 36.1, 36.2  ICD-10-CM procedure codes: 0210093, 02100A3, 02100J3, 02100K3, 02100Z3, 0210493, 02104A3, 02104J3, 02104K3, 02104Z3, 021009W, 02100AW, 02100JW, 02100KW, 021049W, 02104AW, 02104JW, 02104KW, 021109W, 02110AW, 02110JW, 02110KW, 021149W, 02114AW, 02114JW, 02114KW, 021209W, 02120AW, 02120JW, 02120KW, 021249W, 02124AW, 02124JW, 02124KW, 021309W, 02130AW, 02130JW, 02130KW, 021349W, 02134AW, 02134JW, 02134KW, 0210098, 0210099, 021009C, 02100A8, 02100A9, 02100AC, 02100J8, 02100J9, 02100JC, 02100K8, 02100K9, 02100KC, 02100Z8, 02100Z9, 02100ZC, 0210498, 0210499, 021049C, 02104A8, 02104A9, 02104AC, 02104J8,02104J9, 02104JC, 02104K8, 02104K9, 02104KC, 02104Z8, 02104Z9, 02104ZC, 0211098, 0211099, 021109C, 02110A8, 02110A9, 02110AC, 02110J8, 02110J9, 02110JC, 02110K8, 02110K9, 02110KC, 02110Z8, 02110Z9, 02110ZC, 0211498, 0211499, 021149C, 02114A8, 02114A9, 02114AC, 02114J8, 02114J9, 02114JC, 02114K8, 02114K9, 02114KC, 02114Z8, 02114Z9, 02114ZC, 0212098, 0212099, 021209C, 02120A8, 02120A9, 02120AC, 02120J8, 02120J9, 02120JC, 02120K8, 02120K9, 02120KC, 02120Z8, 02120Z9, 02120ZC, 0212498, 0212499, 021249C, 02124A8, 02124A9, 02124AC, 02124J8, 02124J9, 02124JC, 02124K8, 02124K9, 02124KC, 02124Z8, 02124Z9, 02124ZC, 0213098, 0213099, 021309C, 02130A8, 02130A9, 02130AC, 02130J8, 02130J9, 02130JC, 02130K8, 02130K9, 02130KC, 02130Z8, 02130Z9, 02130ZC, 0213498, 0213499, 021349C, 02134A8, 02134A9, 02134AC, 02134J8, 02134J9, 02134JC, 02134K8, 02134K9, 02134KC, 02134Z8, 02134Z9, 02134ZC, 021009F, 02100AF, 02100JF, 02100KF, 02100ZF, 021049F, 02104AF, 02104JF, 02104KF, 02104ZF, 021109F, 02110AF, 02110JF, 02110KF, 02110ZF, 021149F, 02114AF, 02114JF, 02114KF, 02114ZF, 021209F, 02120AF, 02120JF, 02120KF, 02120ZF, 021249F, 02124AF, 02124JF, 02124KF, 02124ZF, 021309F, 02130AF, 02130JF, 02130KF, 02130ZF, 021349F, 02134AF, 02134JF, 02134KF, 02134ZF, 0210093, 02100A3, 02100J3, 02100K3, 02100Z3, 0210493, 02104A3, 02104J3, 02104K3, 02104Z3, 0211093, 02110A3, 02110J3, 02110K3, 02110Z3, 0211493, 02114A3, 02114J3, 02114K3, 02114Z3, 0212093, 02120A3, 02120J3, 02120K3, 02120Z3, 0212493, 02124A3, 02124J3, 02124K3, 02124Z3, 0213093, 02130A3, 02130J3, 02130K3, 02130Z3, 0213493, 02134A3, 02134J3, 02134K3, 02134Z3, 021K0Z8, 021K0Z9, 021K0ZC, 021K0ZF, 021K0ZW, 021K4Z8, 021K4Z9, 021K4ZC, 021K4ZF, 021K4ZW, 021L09P, 021L09Q, 021L09R, 021L0AP, 021L0AQ, 021L0AR, 021L0JP, 021L0JQ, 021L0JR, 021L0KP, 021L0KQ, 021L0KR, 021L0Z8, 021L0Z9, 021L0ZC, 021L0ZF, 021L0ZP, 021L0ZQ, 021L0ZR, 021L4AP, 021L4AQ, 021L4AR, 021L4KP, 021L4KQ, 021L4KR, 021L4Z8, 021L4Z9, 021L4ZC, 021L4ZF, 021L4ZP, 021L4ZQ, 021L4ZR  Taiwan health insurance service claims codes: 68023A, 68023B, 68024A, 68024B, 68025A, 68025B, 83064A1, 97901K, 97902A, 97903B, 97906K, 97907A, 97908B, 97911K, 97912A, 97913B,97916K, 97917A, 97918B |
| Percutaneous transluminal coronary angioplasty | ICD-9-CM procedure codes: 00.66, 36.01, 36.02, 36.05, 36.06, 36.07, 36.09  ICD-10-CM procedure codes: 02703ZZ, 02704ZZ, 3E07317, 02713ZZ, 02714ZZ, 02723ZZ, 02724ZZ, 02733ZZ, 02734ZZ, 02C03ZZ, 02C04ZZ, 02C13ZZ, 02C14ZZ, 02C23ZZ, 02C24ZZ, 02C33ZZ, 02C34ZZ  Taiwan health insurance service claims codes: 33076A, 33076B, 33077A, 33077B, 33078A, 33078B, 97511K, 97512A, 97513B, 97516K, 97517A, 97518B, 97521K, 97522A, 97523B |
| Cardiac dysrhythmia | ICD-9-CM diagnosis codes: 427  ICD-10-CM diagnosis codes: I46-I49, R00.1 |
| Congestive heart failure | ICD-9-CM diagnosis codes: 428, 398.91, 402.01, 402.11, 402.91, 404.01, 404.11, 404.91, 404.03, 404.13, 404.93  ICD-10-CM diagnosis codes: I50, I09.81, I11.0, I13.0, I13.2 |
| Cerebrovascular disease | ICD-9-CM diagnosis codes: 430-438  ICD-10-CM diagnosis codes: I60-I63, I65-I69 (except I67.3, I67.83), G45, G46 |
| Peripheral vascular disease | ICD-9-CM diagnosis codes: 440.2, 440.4, 443.81, 443.9  ICD-10-CM diagnosis codes: I70.2, I70.92, I75.0, I79.1, I79.8, I73.9 |
| Hyperlipidemia | ICD-9-CM diagnosis codes: 272  ICD-10-CM diagnosis codes: E71.30, E75.21, E75.22, E75.24, E75.3, E75.5, E75.6, E77, E78 (except E78.71, E78.72), E88.1, E88.2, E88.9 |
| Diabetes mellitus | ICD-9-CM diagnosis codes: 250  ICD-10-CM diagnosis codes: E08, E09, E10, E11, E13 |
| Chronic lung disease | ICD-9-CM diagnosis codes: 490-496, 500-508  ICD-10-CM diagnosis codes: J40-J47 (except J45.990), J60-J70 (except J70.5) |
| Chronic liver disease | ICD-9-CM diagnosis codes: 070.2, 070.3, 070.41, 070.44, 070.51, 070.54, V02.61, V02.62, 571.0-571.6  ICD-10-CM diagnosis codes: B16, B17, B18.0-B18.2, B19.1, B19.2, Z22.51, Z22.52, K70, K73, K74.0, K74.3-K74.6, K75.4, K75.81 |
| Gastritis or peptic ulcer disease | ICD-9-CM diagnosis codes: 531-535, 578.0, 578.1, 578.9  ICD-10-CM diagnosis codes: K25-K29, K31.82, K52.81, K92.0-K92.2 |
| Chronic kidney disease | ICD-9-CM diagnosis codes: 403.00, 403.01, 403.10, 403.11, 403.90, 403.91, 404.00, 404.01, 404.02, 404.03, 404.10, 404.11, 404.12, 404.13, 404.90, 404.91, 404.92, 404.93, 585, V45.1, V56.0, V56.8  ICD-10-CM diagnosis codes: I12, I13, N18, Z49.3, Z99.2  ICD-9-CM procedure codes: 39.95, 54.98  ICD-10-CM procedure codes: 3E1M39Z, 5A1D00Z, 5A1D60Z |

U.S., United States.

^a^Only applied in the Taiwanese data.

^b^Only applied in the U.S. data.

^c^Lower respiratory tract infections included pneumonia, influenza, empyema, and lung and mediastinum abscess.

^d^Bone infections included osteomyelitis and necrotizing fasciitis.

^e^Acute respiratory tract infections included acute nasopharyngitis, acute sinusitis, acute pharyngitis, acute tonsillitis, acute laryngitis and tracheitis, acute upper respiratory infection of multiple or unspecified sites, and acute bronchitis and bronchiolitis.

^f^Severe head trauma included fracture of skull base, orbital floor, and other skull or facial bones.

^g^Severe eye trauma included contusion of orbital tissues and eyeball, unspecified contusion of eye, penetrating wound of orbit, open wound of eyeball, and burn of eye.

^h^Other major eye surgery included operations on cornea, iris, ciliary body, sclera, anterior chamber, extraocular muscles, orbit, and eyeball.

**Table S6**. Anatomical Therapeutic Chemical (ATC) classification system codes used to identify medication use at baseline^a^

| Medications | ATC classification system codes |
| --- | --- |
| Ophthalmic medications | |
| Anti-bacterials | S01AA, S01AB, S01AE, S01CA, S01CB, S01CC |
| Anti-virals | S01AD |
| Corticosteriods | S01BA, S01BB, S01CA, S01CB |
| NSAIDs | S01BC, S01CC |
| Anti-glaucoma | S01E |
| Anti-neovascularisation agents | S01LA |
|  | |
| Non-ophthalmic medications | |
| ACEIs/ARBs | C09 |
| Beta blocking agents | C07 |
| Calcium channel blockers | C08 |
| Diuretics | C03, C07B, C07C, C07D, C08G |
| Other anti-hypertensive agents | C02 |
| Nitrates | C01DA |
| Anti-arrhythmic agents | C01B |
| Digoxin | C01AA |
| Aspirin | B01AC06, N02BA01 |
| Clopidogrel | B01AC04 |
| Warfarin | B01AA03 |
| New oral anticoagulants | B01AE, B01AF |
| Statins | C10AA |
| Fibrates | C10AB |
| Insulin | A10A |
| Metformin | A10BA02, A10BD02, A10BD03, A10BD05, A10BD07, A10BD08, A10BD10, A10BD11, A10BD13-A10BD18, A10BD20, A10BD22, A10BD23, A10BD25, A10BD26 |
| Sulfonylurea | A10BB, A10BD01, A10BD02, A10BD04, A10BD06 |
| Alpha-glucosidase inhibitors | A10BF, A10BD17 |
| Thiazolidinedione | A10BG, A10BD03-A10BD06, A10BD09, A10BD12, A10BD26 |
| Glinides | A10BX02, A10BX03, A10BX08, A10BD14 |
| Dipeptidyl peptidase-4 inhibitors | A10BH, A10BD07-A10BD13, A10BD18, A10BD19, A10BD21, A10BD22, A10BD24, A10BD25 |
| Glucagon-like peptide-1 receptor agonists | A10BJ |
| Sodium glucose cotransporter 2 inhibitors | A10BK, A10BD15, A10BD16, A10BD19-A10BD21, A10BD23-A10BD25 |
| Non-study antibiotics | J01 (except J01MA, J01DC, J01DD, J01DA18, J01DE, J01CR01, J01CR02, J01CR04) |
| Systemic corticosteroids | H02AB, H02BX |

ACEIs, angiotensin-converting enzyme inhibitors; ARBs, angiotensin II receptor blockers; NSAIDs, Non-steroidal anti-inflammatory drugs.

^a^Based on outpatient visit, emergency department visit, and inpatient (Taiwanese data only) pharmacy dispensing claims.

**Table S7.** Summary of study cohort assembly in each database

|  | Taiwan  NHIRD | U.S.  IBM MarketScan |
| --- | --- | --- |
|  | Number of patients | |
| Patients with a prescription fill of oral fluoroquinolones, amoxicillin/clavulanate or ampicillin/sulbactam, or extended-spectrum cephalosporins during the study period^a^ | 13,187,639 | 27,392,901 |
| Excluded due to: |  |  |
| - Patients without interactions with the healthcare systems within 180 days before the cohort entry date^b^ | 516,499 | 6,321,872 |
| - Patients with any prescription fills of oral or injectable^c^ study antibiotics within 180 days before the cohort entry date^b^ | 337,283 | 592,114 |
| - Patients with any prescription fills of injectable^c^ study antibiotics at cohort entry | 1,486,062 | NA^c^ |
| Patients who initiated oral study antibiotics | 10,847,795 | 20,478,915 |
| Excluded due to |  |  |
| - Patients with age less than 18 years, more than 100 years, or unknown/missing age at cohort entry | 2,542,903 | 4,044,815 |
| - Patients with unknown/missing sex | 173,572 | 0 |
| - Patients receiving more than one class of study antibiotic at cohort entry | 26,399 | 28,807 |
| - Patients receiving more than one fluoroquinolone at cohort entry | 4,042 | 2,097 |
| - Patients with a diagnosis of retinal detachment or defects or receiving retinal-related clinical management before cohort entry^d^ | 149,945 | 120,761 |
| - Patients who were blindness, received evisceration of eyeball, or received enucleation of eyeball^d^ | 5,894 | 55,023 |
| - Patients with death records before cohort entry^e^ | 420 | NA^e^ |
| Eligible patients | 7,944,620 | 16,227,412 |
| - Oral fluoroquinolones | 2,664,929 | 7,472,539 |
| - Oral amoxicillin/clavulanate or ampicillin/sulbactam | 3,739,941 | 6,463,853 |
| - Oral extended-spectrum cephalosporins | 1,539,750 | 2,291,020 |

NA, not applicable; NHIRD, National Health Insurance Research Database; U.S., United States.

^a^Patients with study antibiotic use were identified from the Taiwanese data between 2009/1/1 and 2018/8/31 and from the U.S. data between 2011/1/1 and 2020/9/30.

^b^The cohort entry date was defined as the date of the first dispensing of a study antibiotic.

^c^Exclusion of baseline injectable antibiotic use was only conducted in the Taiwan data.

^d^Information on retinal detachment or defects, retinal-related clinical management, blindness, or evisceration or enucleation of eyeball was looked back as far as early before cohort entry (2008/1/1 for Taiwanese data and 2010/1/1 for the U.S. data).

^e^Death records were not available in the U.S. data.

**Table S8.** Distribution of study antibiotics in the eligible cohort in each database and across databases

|  | Taiwan  NHIRD | U.S.  IBM MarketScan | Overall |
| --- | --- | --- | --- |
|  | Number of patients (%) | | |
| Eligible patients | 7,944,620 | 16,227,412 | 24,172,032 |
| Fluoroquinolones | 2,664,929 (33.54) | 7,472,539 (46.05) | 10,137,468 (41.94) |
| Amoxicillin/clavulanate or ampicillin/sulbactam | 3,739,941 (47.08) | 6,463,853 (39.83) | 10,203,794 (42.21) |
| Extended-spectrum cephalosporins | 1,539,750 (19.38) | 2,291,020 (14.12) | 3,830,770 (15.85) |
|  |  |  |  |
|  | Number of patients (%) | | |
| Fluoroquinolones | 2,664,929 | 7,472,539 | 10,137,468 |
| Respiratory fluoroquinolone | 522,092 (19.59) | 2,490,847 (33.33) | 3,012,939 (29.72) |
| Levofloxacin | 397,188 (14.9) | 2,208,380 (29.55) | 2,605,568 (25.70) |
| Moxifloxacin | 96,839 (3.63) | 274,505 (3.67) | 371,344 (3.66) |
| Gemifloxacin | 28,065 (1.05) | 7,962 (0.11) | 36,027 (0.36) |
| Non-respiratory fluoroquinolones | 2,142,837 (80.41) | 4,981,692 (66.67) | 7,124,529 (70.28) |
| Ofloxacin | 1,246,156 (46.76) | 3,373 (0.05) | 1,249,529 (12.33) |
| Ciprofloxacin | 627,522 (23.55) | 4,978,195 (66.62) | 5,605,717 (55.3) |
| Norfloxacin | 262,896 (9.87) | 0 (0) | 262,896 (2.59) |
| Lomefloxacin | 6,240 (0.23) | 0 (0) | 6,240 (0.06) |
| Enoxacin or sparfloxacin^a^ | 23 (<0.01) | 0 (0) | 23 (<0.01) |
| Delafloxacin | 0 (0) | 124 (<0.01) | 124 (<0.01) |
|  |  | | |
|  | Number of patients (%) | | |
| Amoxicillin/clavulanate or ampicillin/sulbactam | 3,739,941 | 6,463,853 | 10,203,794 |
| Amoxicillin/clavulanate | 3,588,219 (95.94) | 6,463,853 (100) | 10,052,072 (98.51) |
| Sultamicillin | 151,722 (4.06) | 0 (0) | 151,722 (1.49) |
|  |  |  |  |
|  | Number of patients (%) | | |
| Extended-spectrum cephalosporins | 1,539,750 | 2,291,020 | 3,830,770 |
| 2^nd^ generation cephalosporins | 1,285,686 (83.5) | 951,824 (41.55) | 2,237,510 (58.41) |
| Ccefuroxime | 276,441 (17.95) | 822,928 (35.92) | 1,099,369 (28.70) |
| Cefaclor | 1,009,245 (65.55) | 15,650 (0.68) | 1,024,895 (26.75) |
| Cefprozil | 0 (0) | 113,246 (4.94) | 113,246 (2.96) |
| 3^rd^ generation cephalosporins | 254,064 (16.50) | 1,339,196 (58.45) | 1,593,260 (41.59) |
| Cefixime | 154,740 (10.05) | 55,084 (2.40) | 209,824 (5.48) |
| Cefpodoxime | 276 (0.02) | 41,632 (1.82) | 41,908 (1.09) |
| Ceftibuten | 99,048 (6.43) | 0 (0) | 99,048 (2.59) |
| Cefdinir | 0 (0) | 1,242,480 (54.23) | 1,242,480 (32.43) |

NHIRD, National Health Insurance Research Database; U.S., United States.

^a^Data are combined to ensure that a cell number ≤3 for enoxacin or sparfloxacin could not be recalculated, which follows the data protection policy in Taiwan.

**Table S9.** Patient characteristics among initiators of fluoroquinolones and initiators of amoxicillin/clavulanate or ampicillin/sulbactam before and after propensity score matching in the Taiwan NHIRD

|  | Before matching (n=6,404,870) | | | After matching (n=3,954,828) | | |
| --- | --- | --- | --- | --- | --- | --- |
|  | Fluoroquinolones | Amoxicillin/clavulanate or ampicillin/sulbactam | aSMD | Fluoroquinolones | Amoxicillin/clavulanate or ampicillin/sulbactam | aSMD |
| Variables^a^ | n=2,664,929 | n=3,739,941 |  | n=1,977,414 | n=1,977,414 |  |
| Demographics |  |  |  |  |  |  |
| Age in years, mean (SD) | 47.79 (18.34) | 45.11 (17.77) | 0.149 | 47.24 (18.31) | 46.68 (18.01) | 0.031 |
| Male, n (%) | 1,115,914 (41.87) | 1,821,751 (48.71) | -0.138 | 943,170 (47.70) | 941,158 (47.60) | 0.002 |
| Calendar year of the cohort entry date, n (%) | | | | | | |
| 2009 to 2011 | 973,600 (36.53) | 1,111,160 (29.71) | 0.145 | 672,170 (33.99) | 672,170 (33.99) | NA^b^ |
| 2012 to 2014 | 835,279 (31.34) | 1,141,029 (30.51) | 0.018 | 627,096 (31.71) | 627,096 (31.71) | NA^b^ |
| 2015 to 2018 | 856,050 (32.12) | 1,487,752 (39.78) | -0.160 | 678,148 (34.29) | 678,148 (34.29) | NA^b^ |
| Source of index prescription |  |  |  |  |  |  |
| Medical centers | 304,097 (11.41) | 604,971 (16.18) | -0.139 | 253,648 (12.83) | 248,393 (12.56) | 0.008 |
| Regional hospitals | 354,508 (13.30) | 757,283 (20.25) | -0.187 | 276,684 (13.99) | 267,176 (13.51) | 0.014 |
| District hospitals | 245,756 (9.22) | 437,207 (11.69) | -0.081 | 180,338 (9.12) | 179,015 (9.05) | 0.002 |
| Primary care clinics | 1,760,568 (66.06) | 1,940,480 (51.89) | 0.291 | 1,266,744 (64.06) | 1,282,830 (64.87) | -0.017 |
| Acute infection diagnoses, n (%) |  |  |  |  |  |  |
| Lower respiratory tract infections^c^ | 199,376 (7.48) | 336,068 (8.99) | -0.055 | 184,054 (9.31) | 193,115 (9.77) | -0.016 |
| Genitourinary tract infections | 743,858 (27.91) | 142,157 (3.80) | 0.699 | 150,769 (7.62) | 141,893 (7.18) | 0.017 |
| Skin, soft tissue, and bone infections^d^ | 142,322 (5.34) | 454,219 (12.15) | -0.243 | 137,322 (6.94) | 128,453 (6.50) | 0.018 |
| Intra-abdominal infections | 14,398 (0.54) | 25,997 (0.70) | -0.020 | 13,015 (0.66) | 12,867 (0.65) | 0.001 |
| Septicemia | 21,506 (0.81) | 22,899 (0.61) | 0.023 | 15,512 (0.78) | 14,022 (0.71) | 0.009 |
| Acute respiratory tract infections^e^ | 1,169,802 (43.90) | 2,233,353 (59.72) | -0.321 | 1,122,272 (56.75) | 1,135,825 (57.44) | -0.014 |
| Ophthalmic comorbidities, n (%) |  |  |  |  |  |  |
| Endophthalmitis | 1,030 (0.04) | 1,328 (0.04) | 0.002 | 771 (0.04) | 749 (0.04) | 0.001 |
| Intra-ocular foreign body | 246 (0.01) | 248 (0.01) | 0.003 | 177 (0.01) | 156 (0.01) | 0.001 |
| Myopia | 15,117 (0.57) | 26,476 (0.71) | -0.018 | 11,996 (0.61) | 11,527 (0.58) | 0.003 |
| Diabetic retinopathy | 12,460 (0.47) | 15,182 (0.41) | 0.009 | 9,029 (0.46) | 8,672 (0.44) | 0.003 |
| Retinal vascular occlusion | 1,545 (0.06) | 2,085 (0.06) | 0.001 | 1,157 (0.06) | 1,143 (0.06) | <0.001 |
| Other retinal disorders | 27,300 (1.02) | 35,868 (0.96) | 0.007 | 20,134 (1.02) | 19,385 (0.98) | 0.004 |
| Chorioretinitis | 272 (0.01) | 326 (0.01) | 0.002 | 190 (0.01) | 196 (0.01) | <0.001 |
| Glaucoma | 37,792 (1.42) | 50,936 (1.36) | 0.005 | 28,184 (1.43) | 26,922 (1.36) | 0.005 |
| Cataract | 129,133 (4.85) | 148,298 (3.97) | 0.043 | 91,900 (4.65) | 87,883 (4.44) | 0.010 |
| Disorder of orbit | 1,949 (0.07) | 5,509 (0.15) | -0.022 | 1,658 (0.08) | 1,594 (0.08) | 0.001 |
| Disorders of vitreous body | 23,911 (0.90) | 32,714 (0.87) | 0.002 | 17,652 (0.89) | 16,869 (0.85) | 0.004 |
| Severe head trauma^f^ | 1,590 (0.06) | 4,042 (0.11) | -0.017 | 1,309 (0.07) | 1,333 (0.07) | <0.001 |
| Severe eye trauma^g^ | 3,067 (0.12) | 4,914 (0.13) | -0.005 | 2,414 (0.12) | 2,326 (0.12) | 0.001 |
| Cytomegalovirus diseases | 122 (<0.01) | 146 (<0.01) | 0.001 | 96 (<0.01) | 91 (<0.01) | <0.001 |
| Acquired immunodeficiency syndrome | 2,383 (0.09) | 4,614 (0.12) | -0.010 | 2,068 (0.10) | 2,045 (0.10) | <0.001 |
| Intravitreal injection | 323 (0.01) | 409 (0.01) | 0.001 | 226 (0.01) | 248 (0.01) | -0.001 |
| Choroidal, retinal, or vitreous biopsy | ≤3^h^ | ≤3^h^ | NA^h^ | ≤3^h^ | ≤3^h^ | NA^h^ |
| Removal of lesion of retina and choroid | 50 (<0.01) | 56 (<0.01) | 0.001 | 34 (<0.01) | 32 (<0.01) | <0.001 |
| Other major eye surgery^i^ | 1,922 (0.07) | 2,779 (0.07) | -0.001 | 1,422 (0.07) | 1,334 (0.07) | 0.002 |
| Non-ophthalmic comorbidities, n (%) |  |  |  |  |  |  |
| Hypertension | 585,841 (21.98) | 685,930 (18.34) | 0.091 | 422,604 (21.37) | 404,710 (20.47) | 0.022 |
| Ischemic heart disease | 148,145 (5.56) | 179,351 (4.80) | 0.034 | 108,094 (5.47) | 103,990 (5.26) | 0.009 |
| Myocardial infarction | 10,560 (0.40) | 14,269 (0.38) | 0.002 | 8,220 (0.42) | 7,924 (0.40) | 0.002 |
| Coronary artery bypass grafting | 595 (0.02) | 1,485 (0.04) | -0.010 | 545 (0.03) | 548 (0.03) | <0.001 |
| Percutaneous transluminal coronary angioplasty | 4,423 (0.17) | 6,274 (0.17) | <0.001 | 3,492 (0.18) | 3,391 (0.17) | 0.001 |
| Cardiac dysrhythmia | 80,211 (3.01) | 98,536 (2.63) | 0.023 | 58,487 (2.96) | 55,820 (2.82) | 0.008 |
| Congestive heart failure | 53,496 (2.01) | 64,811 (1.73) | 0.020 | 39,967 (2.02) | 38,240 (1.93) | 0.006 |
| Cerebrovascular disease | 104,934 (3.94) | 123,785 (3.31) | 0.034 | 74,632 (3.77) | 70,571 (3.57) | 0.011 |
| Peripheral vascular disease | 15,162 (0.57) | 18,215 (0.49) | 0.011 | 10,849 (0.55) | 10,468 (0.53) | 0.003 |
| Hyperlipidemia | 331,319 (12.43) | 421,374 (11.27) | 0.036 | 241,465 (12.21) | 232,586 (11.76) | 0.014 |
| Diabetes mellitus | 286,814 (10.76) | 345,945 (9.25) | 0.050 | 206,584 (10.45) | 197,583 (9.99) | 0.015 |
| Chronic lung disease | 248,858 (9.34) | 397,843 (10.64) | -0.043 | 208,334 (10.54) | 206,043 (10.42) | 0.004 |
| Chronic liver disease | 102,225 (3.84) | 138,828 (3.71) | 0.007 | 78,443 (3.97) | 75,234 (3.80) | 0.008 |
| Gastritis or peptic ulcer disease | 542,171 (20.34) | 638,699 (17.08) | 0.084 | 388,969 (19.67) | 376,316 (19.03) | 0.016 |
| Chronic kidney disease | 58,565 (2.20) | 67,272 (1.80) | 0.029 | 43,657 (2.21) | 41,810 (2.11) | 0.006 |
| aDCSI (0-13), mean (SD) | 0.10 (0.52) | 0.09 (0.48) | 0.032 | 0.10 (0.52) | 0.10 (0.51) | 0.009 |
| Deyo version of Charlson comorbidity index (0-37), mean (SD) | 0.69 (1.35) | 0.62 (1.27) | 0.051 | 0.70 (1.37) | 0.68 (1.36) | 0.018 |
| Ophthalmic medication use, n (%) |  |  |  |  |  |  |
| Anti-bacterials | 507,941 (19.06) | 714,200 (19.10) | -0.001 | 379,144 (19.17) | 366,599 (18.54) | 0.016 |
| Anti-virals | 1,025 (0.04) | 1,460 (0.04) | <0.001 | 794 (0.04) | 718 (0.04) | 0.002 |
| Corticosteroids | 384,020 (14.41) | 492,127 (13.16) | 0.036 | 280,976 (14.21) | 272,469 (13.78) | 0.012 |
| NSAIDs | 1,412 (0.05) | 1,593 (0.04) | 0.005 | 993 (0.05) | 983 (0.05) | <0.001 |
| Anti-glaucoma | 159,164 (5.97) | 200,131 (5.35) | 0.027 | 115,260 (5.83) | 110,549 (5.59) | 0.010 |
| Anti-neovascularization agents | 107 (<0.01) | 158 (<0.01) | <0.001 | 77 (<0.01) | 85 (<0.01) | -0.001 |
| Non-ophthalmic medication use, n (%) | | | | | | |
| ACEIs/ARBs | 381,498 (14.32) | 468,402 (12.52) | 0.053 | 281,009 (14.21) | 269,551 (13.63) | 0.017 |
| Beta blocking agents | 342,618 (12.86) | 405,341 (10.84) | 0.062 | 244,526 (12.37) | 234,376 (11.85) | 0.016 |
| Calcium channel blockers | 397,587 (14.92) | 459,670 (12.29) | 0.077 | 286,149 (14.47) | 273,668 (13.84) | 0.018 |
| Diuretics | 205,583 (7.71) | 218,561 (5.84) | 0.074 | 144,908 (7.33) | 138,147 (6.99) | 0.013 |
| Other anti-hypertensive agents | 88,090 (3.31) | 75,746 (2.03) | 0.080 | 58,700 (2.97) | 54,668 (2.76) | 0.012 |
| Nitrates | 86,806 (3.26) | 104,093 (2.78) | 0.028 | 63,997 (3.24) | 61,636 (3.12) | 0.007 |
| Anti-arrhythmic agents | 42,890 (1.61) | 59,932 (1.60) | 0.001 | 32,944 (1.67) | 31,354 (1.59) | 0.006 |
| Digoxin | 17,154 (0.64) | 20,380 (0.54) | 0.013 | 12,631 (0.64) | 12,024 (0.61) | 0.004 |
| Aspirin | 209,897 (7.88) | 250,785 (6.71) | 0.045 | 152,131 (7.69) | 145,490 (7.36) | 0.013 |
| Clopidogrel | 32,246 (1.21) | 40,926 (1.09) | 0.011 | 24,597 (1.24) | 23,770 (1.20) | 0.004 |
| Warfarin | 13,299 (0.50) | 19,047 (0.51) | -0.001 | 10,315 (0.52) | 9,952 (0.50) | 0.003 |
| New oral anticoagulants | 4,841 (0.18) | 7,288 (0.19) | -0.003 | 3,851 (0.19) | 3,628 (0.18) | 0.003 |
| Statins | 218,514 (8.20) | 274,620 (7.34) | 0.032 | 160,190 (8.10) | 153,678 (7.77) | 0.012 |
| Fibrates | 49,670 (1.86) | 60,726 (1.62) | 0.018 | 36,541 (1.85) | 35,294 (1.78) | 0.005 |
| Insulin | 59,837 (2.25) | 70,639 (1.89) | 0.025 | 43,953 (2.22) | 42,079 (2.13) | 0.006 |
| Metformin | 203,655 (7.64) | 244,507 (6.54) | 0.043 | 146,718 (7.42) | 140,721 (7.12) | 0.012 |
| Sulfonylurea | 156,442 (5.87) | 178,549 (4.77) | 0.049 | 111,257 (5.63) | 106,707 (5.40) | 0.010 |
| Alpha-glucosidase inhibitors | 34,245 (1.29) | 40,510 (1.08) | 0.019 | 24,657 (1.25) | 23,842 (1.21) | 0.004 |
| Thiazolidinedione | 33,354 (1.25) | 38,021 (1.02) | 0.022 | 23,702 (1.20) | 22,737 (1.15) | 0.005 |
| Glinides | 21,854 (0.82) | 24,241 (0.65) | 0.020 | 15,680 (0.79) | 14,849 (0.75) | 0.005 |
| DPP-4is | 57,198 (2.15) | 78,123 (2.09) | 0.004 | 43,733 (2.21) | 42,168 (2.13) | 0.005 |
| GLP-1 RAs | 489 (0.02) | 910 (0.02) | -0.004 | 391 (0.02) | 370 (0.02) | 0.001 |
| SGLT2is | 2,232 (0.08) | 4,315 (0.12) | -0.010 | 1,787 (0.09) | 1,728 (0.09) | 0.001 |
| Non-study antibiotics | 1,518,073 (56.96) | 2,186,817 (58.47) | -0.031 | 1,101,646 (55.71) | 1,088,923 (55.07) | 0.013 |
| Systemic corticosteroids | 630,285 (23.65) | 825,720 (22.08) | 0.037 | 495,377 (25.05) | 487,856 (24.67) | 0.009 |
| Resource utilization, mean (SD) |  |  |  |  |  |  |
| No. of outpatient visits | 11.15 (8.67) | 10.23 (8.03) | 0.111 | 10.88 (8.48) | 10.66 (8.32) | 0.026 |
| No. of hospitalizations | 0.16 (0.57) | 0.14 (0.53) | 0.028 | 0.16 (0.57) | 0.15 (0.59) | 0.012 |
| No. of ophthalmological outpatient visits | 0.50 (1.40) | 0.43 (1.26) | 0.053 | 0.48 (1.35) | 0.46 (1.36) | 0.013 |
| No. of ophthalmological hospitalizations | <0.001 (0.02) | <0.001 (0.02) | 0.002 | <0.01 (0.02) | <0.01 (0.02) | <0.001 |

ACEIs, angiotensin-converting enzyme inhibitors; aDCSI, adapted diabetes complications severity index; ARBs, angiotensin II receptor blockers; aSMD, absolute standardized mean difference; DPP-4is, dipeptidyl peptidase-4 inhibitors; GLP-1 RAs, glucagon-like peptide-1 receptor agonists; NHIRD, National Health Insurance Research Database; NSAIDs, non-steroidal anti-inflammatory drugs; NA, not applicable; SD, standard deviation; SGLT-2is, sodium glucose cotransporter 2 inhibitors.

^a^Data are presented as number and proportion of patents unless otherwise specified.

^b^Propensity score estimation and matching was conducted separately in each calendar year of the cohort entry date.

^c^Lower respiratory tract infections included pneumonia, influenza, empyema, and lung and mediastinum abscess.

^d^Bone infections included osteomyelitis and necrotizing fasciitis.

^e^Acute respiratory tract infections included acute nasopharyngitis, acute sinusitis, acute pharyngitis, acute tonsillitis, acute laryngitis and tracheitis, acute upper respiratory infection of multiple or unspecified sites, and acute bronchitis and bronchiolitis.

^f^Severe head trauma included fracture of skull base, orbital floor, and other skull or facial bones.

^g^Severe eye trauma included contusion of orbital tissues and eyeball, unspecified contusion of eye, penetrating wound of orbit, open wound of eyeball, and burn of eye.

^h^Data are not presented because of a cell number ≤3, which follows the data protection policy in Taiwan.

^i^Other major eye surgery included operations on cornea, iris, ciliary body, sclera, anterior chamber, extraocular muscles, orbit, and eyeball.

**Table S10.** Patient characteristics among initiators of fluoroquinolones and initiators of amoxicillin/clavulanate or ampicillin/sulbactam before and after propensity score matching in the U.S. IBM MarketScan Database

|  | Before matching (n= 13,936,392) | | | After matching (n=4,971,654) | | |
| --- | --- | --- | --- | --- | --- | --- |
|  | Fluoroquinolones | Amoxicillin/clavulanate or ampicillin/sulbactam | aSMD | Fluoroquinolones | Amoxicillin/clavulanate or ampicillin/sulbactam | aSMD |
| Variables^a^ | n=7,472,539 | n=6,463,853 |  | n=2,485,827 | n=2,485,827 |  |
| Demographics |  |  |  |  |  |  |
| Age in years, mean (SD) | 49.95 (16.71) | 43.57 (15.10) | 0.401 | 49.69 (16.15) | 48.89 (15.63) | 0.050 |
| Male, n (%) | 2,860,805 (38.28) | 2,817,680 (43.59) | -0.108 | 965,929 (38.86) | 943,654 (37.96) | 0.019 |
| Calendar year of the cohort entry date, n (%) | | | | | | |
| 2011 to 2013 | 4,527,138 (60.58) | 2,937,263 (45.44) | 0.307 | 1,499,148 (60.31) | 1,499,148 (60.31) | NA^b^ |
| 2014 to 2016 | 1,925,951 (25.77) | 1,764,913 (27.30) | -0.035 | 605,760 (24.37) | 605,760 (24.37) | NA^b^ |
| 2017 to 2020 | 1,019,450 (13.64) | 1,761,677 (27.25) | -0.342 | 380,919 (15.32) | 380,919 (15.32) | NA^b^ |
| Acute infection diagnoses, n (%) |  |  |  |  |  |  |
| Lower respiratory tract infections^c^ | 341,892 (4.58) | 96,025 (1.49) | 0.181 | 98,285 (3.95) | 78,191 (3.15) | 0.043 |
| Genitourinary tract infections | 1,777,234 (23.78) | 91,419 (1.41) | 0.716 | 103,418 (4.16) | 90,744 (3.65) | 0.026 |
| Skin, soft tissue, and bone infections^d^ | 167,345 (2.24) | 313,513 (4.85) | -0.141 | 100,584 (4.05) | 106,992 (4.30) | -0.012 |
| Intra-abdominal infections | 222,746 (2.98) | 73,698 (1.14) | 0.130 | 98,403 (3.96) | 73,146 (2.94) | 0.056 |
| Septicemia | 79,256 (1.06) | 55,661 (0.86) | 0.021 | 25,053 (1.01) | 20,257 (0.82) | 0.020 |
| Acute respiratory tract infections^e^ | 1,109,249 (14.84) | 2,931,991 (45.36) | -0.706 | 510,127 (20.52) | 486,836 (19.58) | 0.023 |
| Ophthalmic comorbidities, n (%) |  |  |  |  |  |  |
| Endophthalmitis | 1,645 (0.02) | 1,153 (0.02) | <0.001 | 585 (0.02) | 520 (0.02) | <0.001 |
| Intra-ocular foreign body | 363 (<0.01) | 299 (<0.01) | <0.001 | 146 (0.01) | 148 (0.01) | <0.001 |
| Myopia | 41,123 (0.55) | 38,317 (0.59) | -0.005 | 14,340 (0.58) | 14,195 (0.57) | 0.001 |
| Diabetic retinopathy | 41,143 (0.55) | 22,761 (0.35) | 0.030 | 14,658 (0.59) | 13,758 (0.55) | 0.005 |
| Retinal vascular occlusion | 11,972 (0.16) | 5,437 (0.08) | 0.023 | 3,795 (0.15) | 3,341 (0.13) | 0.005 |
| Other retinal disorders | 156,206 (2.09) | 71,500 (1.11) | 0.078 | 49,740 (2.00) | 44,321 (1.78) | 0.016 |
| Chorioretinitis | 1,870 (0.03) | 1,190 (0.02) | 0.006 | 663 (0.03) | 601 (0.02) | 0.006 |
| Glaucoma | 280,052 (3.75) | 145,260 (2.25) | 0.088 | 91,648 (3.69) | 84,317 (3.39) | 0.016 |
| Cataract | 323,131 (4.32) | 165,080 (2.55) | 0.097 | 111,638 (4.49) | 102,635 (4.13) | 0.018 |
| Disorder of orbit | 5,313 (0.07) | 8,596 (0.13) | -0.019 | 2,581 (0.10) | 2,641 (0.11) | -0.003 |
| Disorders of vitreous body | 97,113 (1.30) | 56,647 (0.88) | 0.040 | 32,959 (1.33) | 31,041 (1.25) | 0.007 |
| Severe head trauma^f^ | 3,106 (0.04) | 8,161 (0.13) | -0.031 | 1,390 (0.06) | 1,289 (0.05) | 0.004 |
| Severe eye trauma^g^ | 4,826 (0.06) | 4,536 (0.07) | -0.004 | 1,695 (0.07) | 1,623 (0.07) | <0.001 |
| Cytomegalovirus diseases | 1,070 (0.01) | 497 (0.01) | <0.001 | 476 (0.02) | 372 (0.02) | <0.001 |
| Acquired immunodeficiency syndrome | 17,618 (0.24) | 11,456 (0.18) | 0.013 | 7,321 (0.30) | 7,134 (0.29) | 0.002 |
| Intravitreal injection | 22,362 (0.30) | 9,148 (0.14) | 0.034 | 6,942 (0.28) | 6,023 (0.24) | 0.008 |
| Choroidal, retinal, or vitreous biopsy | 110 (<0.01) | 18 (<0.01) | <0.001 | 23 (<0.01) | 16 (<0.01) | <0.001 |
| Removal of lesion of retina and choroid | 194 (<0.01) | 146 (<0.01) | <0.001 | 66 (<0.01) | 58 (<0.01) | <0.001 |
| Other major eye surgery^h^ | 675,167 (9.04) | 486,228 (7.52) | 0.055 | 266,050 (10.70) | 252,424 (10.16) | 0.018 |
| Non-ophthalmic comorbidities, n (%) |  |  |  |  |  |  |
| Hypertension | 2,067,692 (27.67) | 1,221,988 (18.90) | 0.209 | 714,400 (28.74) | 681,805 (27.43) | 0.029 |
| Ischemic heart disease | 404,120 (5.41) | 182,935 (2.83) | 0.130 | 136,688 (5.50) | 121,373 (4.88) | 0.028 |
| Myocardial infarction | 69,243 (0.93) | 29,335 (0.45) | 0.058 | 22,652 (0.91) | 19,488 (0.78) | 0.014 |
| Coronary artery bypass grafting | 26,731 (0.36) | 9,928 (0.15) | 0.042 | 9,044 (0.36) | 7,728 (0.31) | 0.009 |
| Percutaneous transluminal coronary angioplasty | 13,886 (0.19) | 6,287 (0.10) | 0.024 | 4,572 (0.18) | 4,042 (0.16) | 0.005 |
| Cardiac dysrhythmia | 380,432 (5.09) | 185,586 (2.87) | 0.114 | 130,154 (5.24) | 114,421 (4.60) | 0.030 |
| Congestive heart failure | 153,975 (2.06) | 54,915 (0.85) | 0.101 | 49,859 (2.01) | 40,490 (1.63) | 0.028 |
| Cerebrovascular disease | 202,207 (2.71) | 84,475 (1.31) | 0.100 | 63,861 (2.57) | 55,798 (2.25) | 0.021 |
| Peripheral vascular disease | 121,904 (1.63) | 48,866 (0.76) | 0.080 | 41,846 (1.68) | 36,189 (1.46) | 0.018 |
| Hyperlipidemia | 1,738,384 (23.26) | 1,093,792 (16.92) | 0.159 | 597,043 (24.02) | 584,850 (23.53) | 0.012 |
| Diabetes mellitus | 894,908 (11.98) | 515,935 (7.98) | 0.134 | 312,553 (12.57) | 296,032 (11.91) | 0.020 |
| Chronic lung disease | 794,332 (10.63) | 505,116 (7.81) | 0.098 | 336,466 (13.54) | 319,886 (12.87) | 0.020 |
| Chronic liver disease | 51,266 (0.69) | 26,977 (0.42) | 0.036 | 17,928 (0.72) | 16,117 (0.65) | 0.008 |
| Gastritis or peptic ulcer disease | 262,877 (3.52) | 122,535 (1.90) | 0.100 | 102,010 (4.10) | 88,032 (3.54) | 0.029 |
| Chronic kidney disease | 187,404 (2.51) | 72,252 (1.12) | 0.104 | 60,414 (2.43) | 50,803 (2.04) | 0.026 |
| aDCSI (0-13), mean (SD) | 0.13 (0.65) | 0.07 (0.47) | 0.106 | 0.14 (0.68) | 0.12 (0.64) | 0.030 |
| Deyo version of Charlson comorbidity index (0-37), mean (SD) | 0.65 (1.42) | 0.36 (0.99) | 0.237 | 0.70 (1.41) | 0.62 (1.35) | 0.058 |
| Ophthalmic medication use, n (%) |  |  |  |  |  |  |
| Anti-bacterials | 261,326 (3.50) | 275,475 (4.26) | -0.039 | 106,909 (4.30) | 104,163 (4.19) | 0.005 |
| Anti-virals | 1,954 (0.03) | 1,312 (0.02) | 0.006 | 745 (0.03) | 704 (0.03) | <0.001 |
| Corticosteroids | 138,142 (1.85) | 103,888 (1.61) | 0.018 | 52,591 (2.12) | 50,759 (2.04) | 0.006 |
| NSAIDs | 32,447 (0.43) | 16,659 (0.26) | 0.029 | 10,951 (0.44) | 10,013 (0.40) | 0.006 |
| Anti-glaucoma | 157,574 (2.11) | 64,317 (1.00) | 0.090 | 48,622 (1.96) | 43,572 (1.75) | 0.016 |
| Anti-neovascularization agents | 139 (<0.01) | 75 (<0.01) | <0.001 | 48 (<0.01) | 41 (<0.01) | <0.001 |
| Non-ophthalmic medication use, n (%) | | | | | | |
| ACEIs/ARBs | 1,732,137 (23.18) | 1,062,435 (16.44) | 0.170 | 601,512 (24.20) | 581,726 (23.40) | 0.019 |
| Beta blocking agents | 1,082,678 (14.49) | 593,546 (9.18) | 0.165 | 374,437 (15.06) | 350,753 (14.11) | 0.027 |
| Calcium channel blockers | 762,291 (10.20) | 423,207 (6.55) | 0.132 | 258,498 (10.40) | 242,719 (9.76) | 0.021 |
| Diuretics | 1,301,771 (17.42) | 764,553 (11.83) | 0.159 | 462,058 (18.59) | 441,585 (17.76) | 0.022 |
| Other anti-hypertensive agents | 144,321 (1.93) | 71,506 (1.11) | 0.067 | 49,017 (1.97) | 44,220 (1.78) | 0.014 |
| Nitrates | 112,703 (1.51) | 45,906 (0.71) | 0.076 | 37,871 (1.52) | 32,702 (1.32) | 0.017 |
| Anti-arrhythmic agents | 48,818 (0.65) | 29,656 (0.46) | 0.026 | 18,473 (0.74) | 16,807 (0.68) | 0.007 |
| Digoxin | 49,638 (0.66) | 18,674 (0.29) | 0.054 | 16,220 (0.65) | 13,749 (0.55) | 0.013 |
| Aspirin | 30,913 (0.41) | 19,025 (0.29) | 0.020 | 10,561 (0.43) | 9,753 (0.39) | 0.006 |
| Clopidogrel | 173,280 (2.32) | 74,725 (1.16) | 0.089 | 57,710 (2.32) | 51,373 (2.07) | 0.017 |
| Warfarin | 140,206 (1.88) | 66,453 (1.03) | 0.071 | 51,963 (2.09) | 45,482 (1.83) | 0.019 |
| New oral anticoagulants | 54,902 (0.74) | 32,517 (0.50) | 0.031 | 20,073 (0.81) | 18,266 (0.74) | 0.008 |
| Statins | 1,604,156 (21.47) | 948,122 (14.67) | 0.177 | 549,153 (22.09) | 529,097 (21.29) | 0.019 |
| Fibrates | 183,321 (2.45) | 111,367 (1.72) | 0.051 | 66,393 (2.67) | 63,742 (2.56) | 0.007 |
| Insulin | 217,078 (2.91) | 131,705 (2.04) | 0.056 | 80,299 (3.23) | 75,545 (3.04) | 0.011 |
| Metformin | 547,099 (7.32) | 355,352 (5.50) | 0.074 | 194,450 (7.82) | 189,073 (7.61) | 0.008 |
| Sulfonylurea | 239,718 (3.21) | 131,925 (2.04) | 0.073 | 83,035 (3.34) | 78,792 (3.17) | 0.010 |
| Alpha-glucosidase inhibitors | 2,689 (0.04) | 1,375 (0.02) | 0.012 | 909 (0.04) | 845 (0.03) | 0.005 |
| Thiazolidinedione | 74,780 (1.00) | 38,356 (0.59) | 0.046 | 25,464 (1.02) | 24,313 (0.98) | 0.004 |
| Glinides | 7,336 (0.10) | 3,420 (0.05) | 0.018 | 2,508 (0.10) | 2,267 (0.09) | 0.003 |
| DPP-4is | 127,357 (1.70) | 73,290 (1.13) | 0.048 | 44,335 (1.78) | 42,477 (1.71) | 0.005 |
| GLP-1 RAs | 54,353 (0.73) | 41,384 (0.64) | 0.011 | 20,929 (0.84) | 20,714 (0.83) | 0.001 |
| SGLT2is | 23,890 (0.32) | 25,218 (0.39) | -0.012 | 9,327 (0.38) | 9,320 (0.38) | <0.001 |
| Non-study antibiotics | 3,274,267 (43.82) | 6,463,853 (100.00) | -1.601 | 2,485,827 (100.00) | 2,485,827 (100.00) | <0.001 |
| Systemic corticosteroids | 1,168,113 (15.63) | 1,145,350 (17.72) | -0.056 | 549,986 (22.13) | 548,549 (22.07) | 0.001 |
| Resource utilization, mean (SD) |  |  |  |  |  |  |
| No. of outpatient visits | 7.52 (8.99) | 5.95 (7.30) | 0.192 | 8.39 (8.78) | 8.09 (9.32) | 0.033 |
| No. of hospitalizations | 0.11 (0.40) | 0.06 (0.28) | 0.145 | 0.13 (0.42) | 0.10 (0.38) | 0.075 |
| No. of ophthalmological outpatient visits | 0.13 (0.57) | 0.08 (0.44) | 0.098 | 0.13 (0.58) | 0.12 (0.56) | 0.018 |
| No. of ophthalmological hospitalizations | <0.01 (0.02) | <0.01 (0.01) | <0.001 | <0.01 (0.02) | <0.01 (0.02) | <0.001 |

ACEIs, angiotensin-converting enzyme inhibitors; aDCSI, adapted diabetes complications severity index; ARBs, angiotensin II receptor blockers; aSMD, absolute standardized mean difference; DPP-4is, dipeptidyl peptidase-4 inhibitors; GLP-1 RAs, glucagon-like peptide-1 receptor agonists; NSAIDs, non-steroidal anti-inflammatory drugs; NA, not applicable; SD, standard deviation; SGLT-2is, sodium glucose cotransporter 2 inhibitors; U.S., United States.

^a^Data are presented as number and proportion of patents unless otherwise specified.

^b^Propensity score estimation and matching was conducted separately in each calendar year of the cohort entry date.

^c^Lower respiratory tract infections included pneumonia, influenza, empyema, and lung and mediastinum abscess.

^d^Bone infections included osteomyelitis and necrotizing fasciitis.

^e^Acute respiratory tract infections included acute nasopharyngitis, acute sinusitis, acute pharyngitis, acute tonsillitis, acute laryngitis and tracheitis, acute upper respiratory infection of multiple or unspecified sites, and acute bronchitis and bronchiolitis.

^f^Severe head trauma included fracture of skull base, orbital floor, and other skull or facial bones.

^g^Severe eye trauma included contusion of orbital tissues and eyeball, unspecified contusion of eye, penetrating wound of orbit, open wound of eyeball, and burn of eye.

^h^Other major eye surgery included operations on cornea, iris, ciliary body, sclera, anterior chamber, extraocular muscles, orbit, and eyeball.

**Table S11.** Patient characteristics among initiators of fluoroquinolones and initiators of extended-spectrum cephalosporins before and after propensity score matching in the Taiwan NHIRD

|  | Before matching (n=4,204,679) | | | After matching (n=2,981,968) | | |
| --- | --- | --- | --- | --- | --- | --- |
|  | Fluoroquinolones | Extended-spectrum cephalosporins | aSMD | Fluoroquinolones | Extended-spectrum cephalosporins | aSMD |
| Variables^a^ | n=2,664,929 | n=1,539,750 |  | n=1,490,984 | n=1,490,984 |  |
| Demographics |  |  |  |  |  |  |
| Age in years, mean (SD) | 47.79 (18.34) | 44.96 (17.67) | 0.150 | 46.50 (17.78) | 45.25 (17.71) | 0.070 |
| Male | 1,115,914 (41.87) | 608,333 (39.51) | 0.048 | 608,266 (40.80) | 601,566 (40.35) | 0.009 |
| Calendar year of the cohort entry date, n (%) | | | | | | |
| 2009 to 2011 | 973,600 (36.53) | 593,579 (38.55) | -0.042 | 550,192 (36.90) | 550,192 (36.90) | NA^b^ |
| 2012 to 2014 | 835,279 (31.34) | 449,103 (29.17) | 0.047 | 448,531 (30.08) | 448,531 (30.08) | NA^b^ |
| 2015 to 2018 | 856,050 (32.12) | 497,068 (32.28) | -0.003 | 492,261 (33.02) | 492,261 (33.02) | NA^b^ |
| Source of index prescription |  |  |  |  |  |  |
| Medical centers | 304,097 (11.41) | 161,836 (10.51) | 0.029 | 168,587 (11.31) | 160,099 (10.74) | 0.018 |
| Regional hospitals | 354,508 (13.30) | 144,559 (9.39) | 0.124 | 157,820 (10.58) | 144,231 (9.67) | 0.030 |
| District hospitals | 245,756 (9.22) | 72,155 (4.69) | 0.179 | 82,453 (5.53) | 72,090 (4.84) | 0.031 |
| Primary care clinics | 1,760,568 (66.06) | 1,161,200 (75.41) | -0.207 | 1,082,124 (72.58) | 1,114,564 (74.75) | -0.049 |
| Acute infection diagnoses, n (%) |  |  |  |  |  |  |
| Lower respiratory tract infections^c^ | 199,376 (7.48) | 58,745 (3.82) | 0.159 | 70,820 (4.75) | 58,738 (3.94) | 0.040 |
| Genitourinary tract infections | 743,858 (27.91) | 224,694 (14.59) | 0.330 | 214,550 (14.39) | 224,597 (15.06) | -0.019 |
| Skin, soft tissue, and bone Infections^d^ | 142,322 (5.34) | 74,273 (4.82) | 0.024 | 76,659 (5.14) | 73,630 (4.94) | 0.009 |
| Intra-abdominal infections | 14,398 (0.54) | 10,919 (0.71) | -0.021 | 10,619 (0.71) | 10,717 (0.72) | -0.001 |
| Septicemia | 21,506 (0.81) | 12,209 (0.79) | 0.002 | 12,406 (0.83) | 11,811 (0.79) | 0.004 |
| Acute respiratory tract infections^e^ | 1,169,802 (43.90) | 984,902 (63.97) | -0.411 | 944,278 (63.33) | 936,289 (62.80) | 0.011 |
| Ophthalmic comorbidities, n (%) |  |  |  |  |  |  |
| Endophthalmitis | 1,030 (0.04) | 569 (0.04) | 0.001 | 577 (0.04) | 540 (0.04) | 0.001 |
| Intra-ocular foreign body | 246 (0.01) | 66 (<0.01) | 0.006 | 85 (0.01) | 66 (<0.01) | 0.002 |
| Myopia | 15,117 (0.57) | 10,647 (0.69) | -0.016 | 10,136 (0.68) | 9,965 (0.67) | 0.001 |
| Diabetic retinopathy | 12,460 (0.47) | 5,776 (0.38) | 0.014 | 6,306 (0.42) | 5,701 (0.38) | 0.006 |
| Retinal vascular occlusion | 1,545 (0.06) | 789 (0.05) | 0.003 | 848 (0.06) | 771 (0.05) | 0.002 |
| Other retinal disorders | 27,300 (1.02) | 14,653 (0.95) | 0.007 | 15,275 (1.02) | 14,229 (0.95) | 0.007 |
| Chorioretinitis | 272 (0.01) | 125 (0.01) | 0.002 | 138 (0.01) | 121 (0.01) | 0.001 |
| Glaucoma | 37,792 (1.42) | 20,639 (1.34) | 0.007 | 21,476 (1.44) | 20,084 (1.35) | 0.008 |
| Cataract | 129,133 (4.85) | 63,379 (4.12) | 0.035 | 67,866 (4.55) | 62,217 (4.17) | 0.019 |
| Disorder of orbit | 1,949 (0.07) | 1,117 (0.07) | <0.001 | 1,153 (0.08) | 1,079 (0.07) | 0.002 |
| Disorders of vitreous body | 23,911 (0.90) | 13,313 (0.86) | 0.003 | 13,933 (0.93) | 12,970 (0.87) | 0.007 |
| Severe head trauma^f^ | 1,590 (0.06) | 792 (0.05) | 0.003 | 813 (0.05) | 783 (0.05) | 0.001 |
| Severe eye trauma^g^ | 3,067 (0.12) | 1,649 (0.11) | 0.002 | 1,770 (0.12) | 1,613 (0.11) | 0.003 |
| Cytomegalovirus diseases | 122 (<0.01) | 53 (<0.01) | 0.002 | 55 (<0.01) | 52 (<0.01) | <0.001 |
| Acquired immunodeficiency syndrome | 2,383 (0.09) | 1,172 (0.08) | 0.005 | 1,281 (0.09) | 1,169 (0.08) | 0.003 |
| Intravitreal injection | 323 (0.01) | 188 (0.01) | <0.001 | 182 (0.01) | 180 (0.01) | <0.001 |
| Choroidal, retinal, or vitreous biopsy | ≤3^h^ | ≤3^h^ | NA^h^ | ≤3^h^ | ≤3^h^ | NA^h^ |
| Removal of lesion of retina and choroid | 50 (<0.01) | 18 (<0.01) | 0.002 | 18 (<0.01) | 15 (<0.01) | 0.001 |
| Other major eye surgery^i^ | 1,922 (0.07) | 938 (0.06) | 0.004 | 992 (0.07) | 923 (0.06) | 0.002 |
| Non-ophthalmic comorbidities, n (%) |  |  |  |  |  |  |
| Hypertension | 585,841 (21.98) | 275,683 (17.90) | 0.102 | 300,356 (20.14) | 272,257 (18.26) | 0.048 |
| Ischemic heart disease | 148,145 (5.56) | 69,104 (4.49) | 0.049 | 75,035 (5.03) | 68,106 (4.57) | 0.022 |
| Myocardial infarction | 10,560 (0.40) | 4,872 (0.32) | 0.013 | 5,446 (0.37) | 4,823 (0.32) | 0.007 |
| Coronary artery bypass grafting | 595 (0.02) | 285 (0.02) | 0.003 | 288 (0.02) | 283 (0.02) | <0.001 |
| Percutaneous transluminal coronary angioplasty | 4,423 (0.17) | 2,040 (0.13) | 0.009 | 2,224 (0.15) | 2,015 (0.14) | 0.004 |
| Cardiac dysrhythmia/ atrial fibrillation | 80,211 (3.01) | 38,219 (2.48) | 0.032 | 41,182 (2.76) | 37,532 (2.52) | 0.015 |
| Congestive heart failure | 53,496 (2.01) | 22,353 (1.45) | 0.043 | 24,939 (1.67) | 22,148 (1.49) | 0.015 |
| Cerebrovascular disease | 104,934 (3.94) | 45,472 (2.95) | 0.054 | 50,011 (3.35) | 44,859 (3.01) | 0.020 |
| Peripheral vascular disease | 15,162 (0.57) | 6,783 (0.44) | 0.018 | 7,392 (0.50) | 6,715 (0.45) | 0.007 |
| Hyperlipidemia | 331,319 (12.43) | 169,726 (11.02) | 0.044 | 181,530 (12.18) | 166,796 (11.19) | 0.031 |
| Diabetes mellitus | 286,814 (10.76) | 136,168 (8.84) | 0.065 | 148,332 (9.95) | 134,133 (9.00) | 0.033 |
| Chronic lung disease | 248,858 (9.34) | 116,451 (7.56) | 0.064 | 128,804 (8.64) | 114,805 (7.70) | 0.034 |
| Chronic liver disease | 102,225 (3.84) | 54,288 (3.53) | 0.016 | 57,859 (3.88) | 53,288 (3.57) | 0.016 |
| Gastritis or peptic ulcer disease | 542,171 (20.34) | 269,628 (17.51) | 0.072 | 285,648 (19.16) | 265,773 (17.83) | 0.034 |
| Chronic kidney disease | 58,565 (2.20) | 27,817 (1.81) | 0.028 | 30,149 (2.02) | 27,492 (1.84) | 0.013 |
| aDCSI (0-13), mean (SD) | 0.10 (0.52) | 0.08 (0.45) | 0.049 | 0.09 (0.48) | 0.08 (0.46) | 0.020 |
| Deyo version of Charlson comorbidity index (0-37), mean (SD) | 0.69 (1.35) | 0.55 (1.20) | 0.104 | 0.62 (1.27) | 0.56 (1.21) | 0.048 |
| Ophthalmic medication use, n (%) |  |  |  |  |  |  |
| Anti-bacterials | 507,941 (19.06) | 290,076 (18.84) | 0.006 | 297,448 (19.95) | 278,959 (18.71) | 0.031 |
| Anti-virals | 1,025 (0.04) | 481 (0.03) | 0.004 | 536 (0.04) | 479 (0.03) | 0.002 |
| Corticosteroids | 384,020 (14.41) | 218,738 (14.21) | 0.006 | 224,836 (15.08) | 210,587 (14.12) | 0.027 |
| NSAIDs | 1,412 (0.05) | 688 (0.04) | 0.004 | 711 (0.05) | 679 (0.05) | 0.001 |
| Anti-glaucoma | 159,164 (5.97) | 86,228 (5.60) | 0.016 | 90,002 (6.04) | 83,708 (5.61) | 0.018 |
| Anti-neovascularization agents | 107 (<0.01) | 70 (<0.01) | -0.001 | 63 (<0.01) | 65 (<0.01) | <0.001 |
| Non-ophthalmic medication use, n (%) | | | | | | |
| ACEIs/ ARBs | 381,498 (14.32) | 181,895 (11.81) | 0.074 | 198,185 (13.29) | 179,410 (12.03) | 0.038 |
| Beta blocking agents | 342,618 (12.86) | 166,735 (10.83) | 0.063 | 179,051 (12.01) | 163,762 (10.98) | 0.032 |
| Calcium channel blockers | 397,587 (14.92) | 184,514 (11.98) | 0.086 | 201,240 (13.50) | 182,394 (12.23) | 0.038 |
| Diuretics | 205,583 (7.71) | 87,660 (5.69) | 0.081 | 96,912 (6.50) | 86,735 (5.82) | 0.028 |
| Other anti-hypertensive agents | 88,090 (3.31) | 32,262 (2.10) | 0.075 | 36,209 (2.43) | 32,167 (2.16) | 0.018 |
| Nitrates | 86,806 (3.26) | 37,622 (2.44) | 0.049 | 41,521 (2.78) | 37,218 (2.50) | 0.018 |
| Anti-arrhythmic agents | 42,890 (1.61) | 23,565 (1.53) | 0.006 | 24,232 (1.63) | 23,134 (1.55) | 0.006 |
| Digoxin | 17,154 (0.64) | 6,920 (0.45) | 0.026 | 7,686 (0.52) | 6,843 (0.46) | 0.008 |
| Aspirin | 209,897 (7.88) | 96,015 (6.24) | 0.064 | 105,011 (7.04) | 94,713 (6.35) | 0.028 |
| Clopidogrel | 32,246 (1.21) | 14,744 (0.96) | 0.024 | 15,985 (1.07) | 14,575 (0.98) | 0.009 |
| Warfarin | 13,299 (0.50) | 6,302 (0.41) | 0.013 | 6,759 (0.45) | 6,186 (0.41) | 0.006 |
| New oral anticoagulants | 4,841 (0.18) | 2,511 (0.16) | 0.004 | 2,715 (0.18) | 2,495 (0.17) | 0.004 |
| Statins | 218,514 (8.20) | 109,723 (7.13) | 0.040 | 118,305 (7.93) | 107,967 (7.24) | 0.026 |
| Fibrates | 49,670 (1.86) | 24,057 (1.56) | 0.023 | 25,940 (1.74) | 23,783 (1.60) | 0.011 |
| Insulin | 59,837 (2.25) | 25,751 (1.67) | 0.041 | 28,571 (1.92) | 25,464 (1.71) | 0.016 |
| Metformin | 203,655 (7.64) | 96,385 (6.26) | 0.054 | 105,292 (7.06) | 95,046 (6.37) | 0.027 |
| Sulfonylurea | 156,442 (5.87) | 72,932 (4.74) | 0.051 | 79,810 (5.35) | 71,876 (4.82) | 0.024 |
| Alpha-glucosidase inhibitors | 34,245 (1.29) | 16,067 (1.04) | 0.023 | 17,519 (1.17) | 15,800 (1.06) | 0.011 |
| Thiazolidinedione | 33,354 (1.25) | 15,463 (1.00) | 0.023 | 16,965 (1.14) | 15,260 (1.02) | 0.011 |
| Glinides | 21,854 (0.82) | 9,388 (0.61) | 0.025 | 10,361 (0.69) | 9,297 (0.62) | 0.009 |
| DPP-4is | 57,198 (2.15) | 28,702 (1.86) | 0.020 | 31,310 (2.10) | 28,423 (1.91) | 0.014 |
| GLP-1 RAs | 489 (0.02) | 299 (0.02) | -0.001 | 308 (0.02) | 295 (0.02) | 0.001 |
| SGLT2is | 2,232 (0.08) | 1,234 (0.08) | 0.001 | 1,335 (0.09) | 1,223 (0.08) | 0.003 |
| Non-study antibiotics | 1,518,073 (56.96) | 845,652 (54.92) | 0.041 | 828,257 (55.55) | 816,349 (54.75) | 0.016 |
| Systemic corticosteroids | 630,285 (23.65) | 315,043 (20.46) | 0.077 | 336,047 (22.54) | 313,752 (21.04) | 0.036 |
| Resource utilization, mean (SD) |  |  |  |  |  |  |
| No. of outpatient visits | 11.15 (8.67) | 10.40 (8.05) | 0.090 | 10.93 (8.31) | 10.44 (8.09) | 0.059 |
| No. of hospitalizations | 0.16 (0.57) | 0.12 (0.48) | 0.067 | 0.14 (0.54) | 0.12 (0.48) | 0.023 |
| No. of ophthalmological outpatient visits | 0.50 (1.40) | 0.47 (1.31) | 0.026 | 0.50 (1.36) | 0.47 (1.32) | 0.026 |
| No. of ophthalmological hospitalizations | <0.001 (0.02) | <0.001 (0.02) | 0.007 | <0.01 (0.02) | <0.01 (0.02) | 0.002 |

ACEIs, angiotensin-converting enzyme inhibitors; aDCSI, adapted diabetes complications severity index; ARBs, angiotensin II receptor blockers; aSMD, absolute standardized mean difference; DPP-4is, dipeptidyl peptidase-4 inhibitors; GLP-1 RAs, glucagon-like peptide-1 receptor agonists; NHIRD, National Health Insurance Research Database; NSAIDs, non-steroidal anti-inflammatory drugs; NA, not applicable; SD, standard deviation; SGLT-2is, sodium glucose cotransporter 2 inhibitors.

^a^Data are presented as number and proportion of patents unless otherwise specified.

^b^Propensity score estimation and matching was conducted separately in each calendar year of the cohort entry date.

^c^Lower respiratory tract infections included pneumonia, influenza, empyema, and lung and mediastinum abscess.

^d^Bone infections included osteomyelitis and necrotizing fasciitis.

^e^Acute respiratory tract infections included acute nasopharyngitis, acute sinusitis, acute pharyngitis, acute tonsillitis, acute laryngitis and tracheitis, acute upper respiratory infection of multiple or unspecified sites, and acute bronchitis and bronchiolitis.

^f^Severe head trauma included fracture of skull base, orbital floor, and other skull or facial bones.

^g^Severe eye trauma included contusion of orbital tissues and eyeball, unspecified contusion of eye, penetrating wound of orbit, open wound of eyeball, and burn of eye.

^h^Data are not presented because of a cell number ≤3, which follows the data protection policy in Taiwan.

^i^Other major eye surgery included operations on cornea, iris, ciliary body, sclera, anterior chamber, extraocular muscles, orbit, and eyeball.

**Table S12.** Patient characteristics among initiators of fluoroquinolones and initiators of extended-spectrum cephalosporins before and after propensity score matching in the U.S. IBM MarketScan Database

|  | Before matching (n=9,763,559) | | | After matching (n=4,028,750) | | |
| --- | --- | --- | --- | --- | --- | --- |
|  | Fluoroquinolones | Extended-spectrum cephalosporins | aSMD | Fluoroquinolones | Extended-spectrum cephalosporins | aSMD |
| Variables^a^ | n=7,472,539 | n=2,291,020 |  | n=2,014,375 | n=2,014,375 |  |
| Demographics |  |  |  |  |  |  |
| Age in years, mean (SD) | 49.95 (16.71) | 44.75 (16.27) | 0.315 | 47.84 (15.81) | 46.50 (15.93) | 0.084 |
| Male, n (%) | 2,860,805 (38.28) | 891,124 (38.90) | -0.013 | 816,658 (40.54) | 798,959 (39.66) | 0.018 |
| Calendar year of the cohort entry date, n (%) | | | | | | |
| 2011 to 2013 | 4,527,138 (60.58) | 1,249,337 (54.53) | 0.123 | 1,160,647 (57.62) | 1,160,647 (57.62) | NA^b^ |
| 2014 to 2016 | 1,925,951 (25.77) | 560,620 (24.47) | 0.030 | 501,796 (24.91) | 501,796 (24.91) | NA^b^ |
| 2017 to 2020 | 1,019,450 (13.64) | 481,063 (21.00) | -0.195 | 351,932 (17.47) | 351,932 (17.47) | NA^b^ |
| Acute infection diagnoses, n (%) |  |  |  |  |  |  |
| Lower respiratory tract infections^c^ | 341,892 (4.58) | 82,925 (3.62) | 0.048 | 97,780 (4.85) | 80,110 (3.98) | 0.042 |
| Genitourinary tract infections | 1,777,234 (23.78) | 92,613 (4.04) | 0.595 | 90,215 (4.48) | 92,428 (4.59) | -0.005 |
| Skin, soft tissue, and bone infections^d^ | 167,345 (2.24) | 45,387 (1.98) | 0.018 | 57,288 (2.84) | 43,821 (2.18) | 0.042 |
| Intra-abdominal infections | 222,746 (2.98) | 5,820 (0.25) | 0.218 | 10,411 (0.52) | 5,810 (0.29) | 0.036 |
| Septicemia | 79,256 (1.06) | 24,572 (1.07) | -0.001 | 28,465 (1.41) | 20,605 (1.02) | 0.036 |
| Acute respiratory tract infections^e^ | 1,109,249 (14.84) | 1,195,243 (52.17) | -0.861 | 923,546 (45.85) | 918,780 (45.61) | 0.005 |
| Ophthalmic comorbidities, n (%) |  |  |  |  |  |  |
| Endophthalmitis | 1,645 (0.02) | 322 (0.01) | 0.008 | 407 (0.02) | 304 (0.02) | <0.001 |
| Intra-ocular foreign body | 363 (<0.01) | 83 (<0.01) | <0.001 | 104 (0.01) | 76 (<0.01) | 0.014 |
| Myopia | 41,123 (0.55) | 12,811 (0.56) | -0.001 | 14,458 (0.72) | 11,157 (0.55) | 0.021 |
| Diabetic retinopathy | 41,143 (0.55) | 8,393 (0.37) | 0.027 | 10,963 (0.54) | 8,154 (0.41) | 0.019 |
| Retinal vascular occlusion | 11,972 (0.16) | 2,456 (0.11) | 0.014 | 3,161 (0.16) | 2,354 (0.12) | 0.011 |
| Other retinal disorders | 156,206 (2.09) | 32,472 (1.42) | 0.051 | 41,264 (2.05) | 30,978 (1.54) | 0.038 |
| Chorioretinitis | 1,870 (0.03) | 489 (0.02) | 0.006 | 546 (0.03) | 439 (0.02) | 0.006 |
| Glaucoma | 280,052 (3.75) | 60,491 (2.64) | 0.063 | 74,809 (3.71) | 57,845 (2.87) | 0.047 |
| Cataract | 323,131 (4.32) | 69,057 (3.01) | 0.070 | 86,219 (4.28) | 66,501 (3.30) | 0.051 |
| Disorder of orbit | 5,313 (0.07) | 1,828 (0.08) | -0.004 | 2,286 (0.11) | 1,654 (0.08) | 0.010 |
| Disorders of vitreous body | 97,113 (1.30) | 22,655 (0.99) | 0.029 | 27,774 (1.38) | 21,355 (1.06) | 0.029 |
| Severe head trauma^f^ | 3,106 (0.04) | 899 (0.04) | <0.001 | 1,222 (0.06) | 833 (0.04) | 0.009 |
| Severe eye trauma^g^ | 4,826 (0.06) | 1,176 (0.05) | 0.004 | 1,445 (0.07) | 1,068 (0.05) | 0.008 |
| Cytomegalovirus diseases | 1,070 (0.01) | 220 (0.01) | <0.001 | 297 (0.02) | 213 (0.01) | 0.008 |
| Acquired immunodeficiency syndrome | 17,618 (0.24) | 3,283 (0.14) | 0.023 | 4,127 (0.21) | 3,210 (0.16) | 0.012 |
| Intravitreal injection | 22,362 (0.30) | 4,280 (0.19) | 0.022 | 5,670 (0.28) | 4,135 (0.21) | 0.014 |
| Choroidal, retinal, or vitreous biopsy | 110 (<0.01) | 8 (<0.01) | <0.001 | 9 (<0.01) | 6 (<0.01) | <0.001 |
| Removal of lesion of retina and choroid | 194 (<0.01) | 45 (<0.01) | <0.001 | 51 (<0.01) | 42 (<0.01) | <0.001 |
| Other major eye surgery^h^ | 675,167 (9.04) | 167,783 (7.32) | 0.063 | 194,584 (9.66) | 155,438 (7.72) | 0.069 |
| Non-ophthalmic comorbidities, n (%) |  |  |  |  |  |  |
| Hypertension | 2,067,692 (27.67) | 482,844 (21.08) | 0.154 | 573,105 (28.45) | 461,888 (22.93) | 0.127 |
| Ischemic heart disease | 404,120 (5.41) | 86,000 (3.75) | 0.079 | 110,791 (5.50) | 83,004 (4.12) | 0.065 |
| Myocardial infarction | 69,243 (0.93) | 14,410 (0.63) | 0.034 | 19,165 (0.95) | 13,826 (0.69) | 0.029 |
| Coronary artery bypass grafting | 26,731 (0.36) | 3,755 (0.16) | 0.039 | 5,480 (0.27) | 3,707 (0.18) | 0.019 |
| Percutaneous transluminal coronary angioplasty | 13,886 (0.19) | 2,876 (0.13) | 0.015 | 3,787 (0.19) | 2,759 (0.14) | 0.012 |
| Cardiac dysrhythmia | 380,432 (5.09) | 90,942 (3.97) | 0.054 | 115,798 (5.75) | 84,548 (4.20) | 0.071 |
| Congestive heart failure | 153,975 (2.06) | 33,370 (1.46) | 0.046 | 45,655 (2.27) | 31,950 (1.59) | 0.049 |
| Cerebrovascular disease | 202,207 (2.71) | 40,869 (1.78) | 0.063 | 54,161 (2.69) | 39,300 (1.95) | 0.049 |
| Peripheral vascular disease | 121,904 (1.63) | 21,046 (0.92) | 0.063 | 28,540 (1.42) | 20,619 (1.02) | 0.036 |
| Hyperlipidemia | 1,738,384 (23.26) | 421,416 (18.39) | 0.120 | 485,101 (24.08) | 403,223 (20.02) | 0.098 |
| Diabetes mellitus | 894,908 (11.98) | 197,599 (8.62) | 0.111 | 245,822 (12.20) | 190,314 (9.45) | 0.089 |
| Chronic lung disease | 794,332 (10.63) | 241,835 (10.56) | 0.002 | 282,269 (14.01) | 225,847 (11.21) | 0.084 |
| Chronic liver disease | 51,266 (0.69) | 9,736 (0.42) | 0.036 | 12,702 (0.63) | 9,373 (0.47) | 0.022 |
| Gastritis or peptic ulcer disease | 262,877 (3.52) | 49,234 (2.15) | 0.083 | 58,503 (2.90) | 47,710 (2.37) | 0.033 |
| Chronic kidney disease | 187,404 (2.51) | 34,986 (1.53) | 0.070 | 47,354 (2.35) | 33,850 (1.68) | 0.048 |
| aDCSI (0-13), mean (SD) | 0.13 (0.65) | 0.08 (0.51) | 0.086 | 0.13 (0.640) | 0.09 (0.538) | 0.068 |
| Deyo version of Charlson comorbidity index (0-37), mean (SD) | 0.65 (1.42) | 0.43 (1.06) | 0.176 | 0.62 (1.27) | 0.47 (1.11) | 0.126 |
| Ophthalmic medication use, n (%) |  |  |  |  |  |  |
| Anti-bacterials | 261,326 (3.50) | 97,319 (4.25) | -0.039 | 105,333 (5.23) | 81,908 (4.07) | 0.055 |
| Anti-virals | 1,954 (0.03) | 552 (0.02) | 0.006 | 705 (0.04) | 508 (0.03) | 0.005 |
| Corticosteroids | 138,142 (1.85) | 39,559 (1.73) | 0.009 | 47,246 (2.35) | 35,859 (1.78) | 0.040 |
| NSAIDs | 32,447 (0.43) | 7,109 (0.31) | 0.020 | 8,869 (0.44) | 6,812 (0.34) | 0.016 |
| Anti-glaucoma | 157,574 (2.11) | 28,390 (1.24) | 0.068 | 36,421 (1.81) | 27,819 (1.38) | 0.034 |
| Anti-neovascularization agents | 139 (<0.01) | 28 (<0.01) | <0.001 | 41 (<0.01) | 27 (<0.01) | <0.001 |
| Non-ophthalmic medication use, n (%) | | | | | | |
| ACEIs/ARBs | 1,732,137 (23.18) | 421,193 (18.39) | 0.118 | 496,124 (24.63) | 403,013 (20.01) | 0.111 |
| Beta blocking agents | 1,082,678 (14.49) | 256,750 (11.21) | 0.098 | 312,820 (15.53) | 243,638 (12.10) | 0.100 |
| Calcium channel blockers | 762,291 (10.20) | 175,518 (7.66) | 0.089 | 215,603 (10.70) | 168,113 (8.35) | 0.080 |
| Diuretics | 1,301,771 (17.42) | 317,169 (13.84) | 0.099 | 375,764 (18.65) | 302,430 (15.01) | 0.097 |
| Other anti-hypertensive agents | 144,321 (1.93) | 31,051 (1.36) | 0.045 | 39,798 (1.98) | 29,511 (1.47) | 0.039 |
| Nitrates | 112,703 (1.51) | 23,567 (1.03) | 0.043 | 31,095 (1.54) | 22,877 (1.14) | 0.035 |
| Anti-arrhythmic agents | 48,818 (0.65) | 17,610 (0.77) | -0.014 | 21,274 (1.06) | 15,249 (0.76) | 0.032 |
| Digoxin | 49,638 (0.66) | 12,101 (0.53) | 0.017 | 16,105 (0.80) | 11,449 (0.57) | 0.028 |
| Aspirin | 30,913 (0.41) | 6,621 (0.29) | 0.020 | 8,414 (0.42) | 6,251 (0.31) | 0.018 |
| Clopidogrel | 173,280 (2.32) | 35,749 (1.56) | 0.055 | 45,982 (2.28) | 34,808 (1.73) | 0.039 |
| Warfarin | 140,206 (1.88) | 39,513 (1.73) | 0.011 | 51,643 (2.56) | 36,282 (1.80) | 0.052 |
| New oral anticoagulants | 54,902 (0.74) | 15,664 (0.68) | 0.007 | 19,353 (0.96) | 14,207 (0.71) | 0.027 |
| Statins | 1,604,156 (21.47) | 381,222 (16.64) | 0.123 | 449,424 (22.31) | 366,535 (18.20) | 0.102 |
| Fibrates | 183,321 (2.45) | 45,693 (1.99) | 0.031 | 55,406 (2.75) | 44,227 (2.20) | 0.035 |
| Insulin | 217,078 (2.91) | 48,714 (2.13) | 0.050 | 62,041 (3.08) | 46,625 (2.32) | 0.047 |
| Metformin | 547,099 (7.32) | 131,717 (5.75) | 0.064 | 159,032 (7.90) | 125,070 (6.21) | 0.066 |
| Sulfonylurea | 239,718 (3.21) | 51,328 (2.24) | 0.060 | 64,900 (3.22) | 49,849 (2.48) | 0.044 |
| Alpha-glucosidase inhibitors | 2,689 (0.04) | 597 (0.03) | 0.005 | 788 (0.04) | 570 (0.03) | 0.005 |
| Thiazolidinedione | 74,780 (1.00) | 16,236 (0.71) | 0.032 | 20,165 (1.00) | 15,886 (0.79) | 0.022 |
| Glinides | 7,336 (0.10) | 1,503 (0.07) | 0.010 | 1,963 (0.10) | 1,460 (0.07) | 0.010 |
| DPP-4is | 127,357 (1.70) | 30,091 (1.31) | 0.032 | 37,371 (1.86) | 29,015 (1.44) | 0.033 |
| GLP-1 RAs | 54,353 (0.73) | 15,557 (0.68) | 0.006 | 18,385 (0.91) | 14,395 (0.72) | 0.021 |
| SGLT2is | 23,890 (0.32) | 8,330 (0.36) | -0.007 | 9,257 (0.46) | 7,288 (0.36) | 0.016 |
| Non-study antibiotics | 3,274,267 (43.82) | 890,353 (38.86) | 0.101 | 857,143 (42.55) | 825,449 (40.98) | 0.032 |
| Systemic corticosteroids | 1,168,113 (15.63) | 506,762 (22.12) | -0.166 | 513,017 (25.47) | 435,916 (21.64) | 0.090 |
| Resource utilization, mean (SD) |  |  |  |  |  |  |
| No. of outpatient visits | 7.52 (8.99) | 6.56 (7.73) | 0.114 | 8.04 (8.84) | 6.79 (7.94) | 0.149 |
| No. of hospitalizations | 0.11 (0.40) | 0.07 (0.31) | 0.113 | 0.11 (0.40) | 0.07 (0.32) | 0.111 |
| No. of ophthalmological outpatient visits | 0.13 (0.57) | 0.09 (0.48) | 0.076 | 0.13 (0.57) | 0.10 (0.50) | 0.056 |
| No. of ophthalmological hospitalizations | <0.01 (0.02) | <0.01 (0.01) | <0.001 | <0.01 (0.01) | <0.01 (0.01) | <0.001 |

ACEIs, angiotensin-converting enzyme inhibitors; aDCSI, adapted diabetes complications severity index; ARBs, angiotensin II receptor blockers; aSMD, absolute standardized mean difference; DPP-4is, dipeptidyl peptidase-4 inhibitors; GLP-1 RAs, glucagon-like peptide-1 receptor agonists; NSAIDs, non-steroidal anti-inflammatory drugs; NA, not applicable; SD, standard deviation; SGLT-2is, sodium glucose cotransporter 2 inhibitors; U.S., United States.

^a^Data are presented as number and proportion of patents unless otherwise specified.

^b^Propensity score estimation and matching was conducted separately in each calendar year of the cohort entry date.

^c^Lower respiratory tract infections included pneumonia, influenza, empyema, and lung and mediastinum abscess.

^d^Bone infections included osteomyelitis and necrotizing fasciitis.

^e^Acute respiratory tract infections included acute nasopharyngitis, acute sinusitis, acute pharyngitis, acute tonsillitis, acute laryngitis and tracheitis, acute upper respiratory infection of multiple or unspecified sites, and acute bronchitis and bronchiolitis.

^f^Severe head trauma included fracture of skull base, orbital floor, and other skull or facial bones.

^g^Severe eye trauma included contusion of orbital tissues and eyeball, unspecified contusion of eye, penetrating wound of orbit, open wound of eyeball, and burn of eye.

^h^Other major eye surgery included operations on cornea, iris, ciliary body, sclera, anterior chamber, extraocular muscles, orbit, and eyeball.

**Table S13.** Number of patients and events, follow-up duration, and incidence rate of rhegmatogenous retinal detachment comparing fluoroquinolones to amoxicillin/clavulanate or ampicillin/sulbactam before and after propensity score matching in each database

|  | Before matching (n=20,341,262) | | After matching (n=8,926,482) | |
| --- | --- | --- | --- | --- |
|  | Fluoroquinolones | Amoxicillin/clavulanate or ampicillin/sulbactam | Fluoroquinolones | Amoxicillin/clavulanate or ampicillin/sulbactam |
|  | Taiwan NHIRD | | | |
| Number of patients | 2,664,929 | 3,739,941 | 1,977,414 | 1,977,414 |
| Number of events | 154 | 277 | 121 | 144 |
| Mean follow-up days, mean (SD) | 89.67 (4.49) | 89.74 (3.99) | 89.65 (4.59) | 89.67 (4.49) |
| Incidence rate (95% CI)^a^ | 0.24 (0.20 ,0.28) | 0.30 (0.27, 0.34) | 0.25 (0.21, 0.30) | 0.30 (0.25, 0.35) |
|  |  | | | |
|  | U.S. IBM MarketScan | | | |
| Number of patients | 7,472,539 | 6,463,853 | 2,485,827 | 2,485,827 |
| Number of events | 836 | 507 | 253 | 234 |
| Mean follow-up days, mean (SD) | 86.15 (14.59) | 85.84 (15.32) | 86.18 (14.61) | 85.84 (15.28) |
| Incidence rate (95% CI)^a^ | 0.47 (0.44, 0.51) | 0.33 (0.31, 0.36) | 0.43 (0.38, 0.49) | 0.40 (0.35, 0.46) |

CI, confidence intervals; NHIRD, National Health Insurance Research Database; SD, standard deviation; U.S., United States.

^a^Per 1,000 person-years.

**Table S14.** Number of patients and events, follow-up duration, and incidence rate of rhegmatogenous retinal detachment comparing fluoroquinolones to extended-spectrum cephalosporins before and after propensity score matching in each database

|  | Before matching (n=13,968,238) | | After matching (n=7,010,718) | |
| --- | --- | --- | --- | --- |
|  | Fluoroquinolones | Extended-spectrum cephalosporins | Fluoroquinolones | Extended-spectrum cephalosporins |
|  | Taiwan NHIRD | | | |
| Number of patients | 2,664,929 | 1,539,750 | 1,490,984 | 1,490,984 |
| Number of events | 154 | 104 | 101 | 99 |
| Mean follow-up days, mean (SD) | 89.67 (4.49) | 89.79 (3.55) | 89.75 (3.88) | 89.78 (3.61) |
| Incidence rate (95% CI)^a^ | 0.24 (0.20,0.28) | 0.27 (0.23,0.33) | 0.28 (0.23,0.34) | 0.27 (0.22,0.33) |
|  |  | | | |
|  | U.S. IBM MarketScan | | | |
| Number of patients | 7,472,539 | 2,291,020 | 2,014,375 | 2,014,375 |
| Number of events | 836 | 199 | 215 | 193 |
| Mean follow-up days, mean (SD) | 86.15 (14.59) | 85.85 (15.32) | 85.94 (15.07) | 85.84 (15.36) |
| Incidence rate (95% CI)^a^ | 0.47 (0.44, 0.51) | 0.37 (0.32, 0.42) | 0.45 (0.40, 0.52) | 0.41 (0.35, 0.47) |

CI, confidence intervals; NHIRD, National Health Insurance Research Database; SD, standard deviation; U.S., United States.

^a^Per 1,000 person-years

**Table S15.** Association between fluoroquinolones versus amoxicillin/clavulanate ampicillin/sulbactam and rhegmatogenous retinal detachment before and after propensity score matching in each database

|  | HR (95% CI) | | | |
| --- | --- | --- | --- | --- |
|  | Before matching | | After matching | |
|  | Fluoroquinolones | Amoxicillin/clavulanate or ampicillin/sulbactam | Fluoroquinolones | Amoxicillin/clavulanate or ampicillin/sulbactam |
| Taiwan NHIRD | 0.78 (0.64, 0.95) | Ref | 0.84 (0.66, 1.07) | Ref |
| U.S. IBM MarketScan | 1.42 (1.27, 1.59) | Ref | 1.08 (0.90, 1.29) | Ref |
| *P*-value for heterogeneity testing | <0.001 | | 0.101 | |

CI, confidence intervals; HR, hazard ratios; NHIRD, National Health Insurance Research Database; U.S., United States.

**Table S16.** Association between fluoroquinolones versus extended-spectrum cephalosporins and rhegmatogenous retinal detachment before and after propensity score matching

|  | HR (95% CI) | | | |
| --- | --- | --- | --- | --- |
|  | Before matching | | After matching | |
|  | Fluoroquinolones | Extended-spectrum cephalosporins | Fluoroquinolones | Extended-spectrum cephalosporins |
| Taiwan NHIRD | 0.86 (0.67, 1.10) | Ref | 1.02 (0.77, 1.35) | Ref |
| U.S. IBM MarketScan | 1.28 (1.10, 1.50) | Ref | 1.11 (0.92, 1.35) | Ref |
| *P*-value for heterogeneity testing | 0.008 | | 0.628 | |

CI, confidence intervals; HR, hazard ratios; NHIRD, National Health Insurance Research Database; U.S., United States.

**Table S17.** Sensitivity analysis of number of patients and events, incidence rate, and HR of rhegmatogenous retinal detachment comparing fluoroquinolones to amoxicillin/clavulanate or ampicillin/sulbactam before and after propensity score matching across databases, by follow-up duration

|  | Before matching | | After matching | |
| --- | --- | --- | --- | --- |
|  | Fluoroquinolones | Amoxicillin/clavulanate or ampicillin/sulbactam | Fluoroquinolones | Amoxicillin/clavulanate or ampicillin/sulbactam |
|  | 1 to 60 days | | | |
| Number of patients | 10,137,468 | 10,203,794 | 4,463,241 | 4,463,241 |
| Number of events | 689 | 520 | 247 | 242 |
| Mean follow-up days (SD) | 58.72 (6.94) | 45.86 (6.83) | 52.48 (6.13) | 52.39 (6.41) |
| Incidence rate (95% CI)^a,b^ | 0.35 (0.17, 0.69) | 0.31 (0.28, 0.36) | 0.33 (0.21, 0.51) | 0.33 (0.26, 0.42) |
| HR (95% CI)^b^ | 1.12 (0.65, 1.94) | Ref | 1.02 (0.85, 1.22) | Ref |
|  |  | | | |
|  | 1 to 30 days | | | |
| Number of patients | 10,137,468 | 10,203,794 | 4,463,241 | 4,463,241 |
| Number of events | 337 | 252 | 114 | 108 |
| Mean follow-up days (SD) | 29.69 (2.41) | 23.23 (2.41) | 26.49 (2.14) | 26.47 (2.26) |
| Incidence rate (95% CI)^a,b^ | 0.32 (0.13, 0.76) | 0.30 (0.27, 0.34) | 0.29 (0.14, 0.59) | 0.30 (0.25, 0.36) |
| HR (95% CI)^b^ | 1.05 (0.48, 2.31) | Ref | 0.98 (0.54, 1.77) | Ref |
|  |  | | | |
|  | 1 to 14 days | | | |
| Number of patients | 10,137,468 | 10,203,794 | 4,463,241 | 4,463,241 |
| Number of events | 143 | 113 | 45 | 46 |
| Mean follow-up days (SD) | 13.94 (0.74) | 10.92 (0.76) | 12.43 (0.66) | 12.42 (0.71) |
| Incidence rate (95% CI)^a,b^ | 0.28 (0.11, 0.71) | 0.29 (0.24, 0.35) | 0.25 (0.11, 0.53) | 0.27 (0.20, 0.35) |
| HR (95% CI)^b^ | 0.97 (0.38, 2.47) | Ref | 0.91 (0.44, 1.89) | Ref |

CI, confidence intervals; HR, hazard ratios; SD, standard deviation.

^a^Per 1,000 person-years.

^b^Data are pooled across databases using random-effects meta-analysis.

**Table S18.** Sensitivity analysis of number of patients and events, incidence rate, and HR of rhegmatogenous retinal detachment comparing fluoroquinolones to extended-spectrum cephalosporins before and after propensity score matching across databases, by follow-up duration

|  | Before matching | | After matching | |
| --- | --- | --- | --- | --- |
|  | Fluoroquinolones | Extended-spectrum cephalosporins | Fluoroquinolones | Extended-spectrum cephalosporins |
|  | 1 to 60 days | | | |
| Number of patients | 10,137,468 | 3,830,770 | 3,505,359 | 3,505,359 |
| Number of events | 689 | 215 | 230 | 207 |
| Mean follow-up days (SD) | 58.72 (6.94) | 58.84 (6.66) | 58.91 (6.43) | 58.87 (6.56) |
| Incidence rate (95% CI)^a,b^ | 0.35 (0.17, 0.69) | 0.35 (0.29, 0.43) | 0.38 (0.23, 0.64) | 0.36 (0.27, 0.47) |
| HR (95% CI)^b^ | 1.03 (0.63, 1.67) | Ref | 1.10 (0.86,1.40) | Ref |
|  |  | | | |
|  | 1 to 30 days | | | |
| Number of patients | 10,137,468 | 3,830,770 | 3,505,359 | 3,505,359 |
| Number of events | 337 | 101 | 104 | 98 |
| Mean follow-up days (SD) | 29.69 (2.41) | 29.71 (2.36) | 29.73 (2.25) | 29.72 (2.32) |
| Incidence rate (95% CI)^a,b^ | 0.32 (0.13, 0.76) | 0.31 (0.21, 0.46) | 0.34 (0.19, 0.61) | 0.33 (0.21, 0.52) |
| HR (95% CI)^b^ | 1.05 (0.64,1.72) | Ref | 1.06 (0.81,1.40) | Ref |
|  |  | | | |
|  | 1 to 14 days | | | |
| Number of patients | 10,137,468 | 3,830,770 | 3,505,359 | 3,505,359 |
| Number of events | 143 | 32 | 47 | 32 |
| Mean follow-up days (SD) | 13.94 (0.74) | 13.94 (0.74) | 13.95 (0.70) | 13.94 (0.72) |
| Incidence rate (95% CI)^a,b^ | 0.28 (0.11, 0.71) | 0.22 (0.16, 0.32) | 0.31 (0.13, 0.75) | 0.24 (0.17, 0.34) |
| HR (95% CI)^b^ | 1.36 (0.68, 2.74) | Ref | 1.45 (0.89, 2.36) | Ref |

CI, confidence intervals; HR, hazard ratios; SD, standard deviation.

^a^Per 1,000 person-years.

^b^Data are pooled across databases using random-effects meta-analysis.

**Table S19.** Subgroup analysis of number of patients and events, incidence rate, and HR of rhegmatogenous retinal detachment comparing fluoroquinolones to amoxicillin/clavulanate or ampicillin/sulbactam before and after propensity score matching across databases, by patient characteristic

|  | Before matching | | After matching | |
| --- | --- | --- | --- | --- |
|  | Fluoroquinolones | Amoxicillin/clavulanate or ampicillin/sulbactam | Fluoroquinolones | Amoxicillin/clavulanate or ampicillin/sulbactam |
|  | ≥65 years | | | |
| Number of patients | 1,750,204 | 1,007,962 | 710,611 | 710,611 |
| Number of events | 250 | 114 | 69 | 78 |
| Mean follow-up days (SD) | 87.22 (12.40) | 87.72 (11.43) | 87.70 (11.42) | 87.59 (11.73) |
| Incidence rate (95% CI)^a,b^ | 0.42 (0.12, 1.42) | 0.45 (0.16, 1.28) | 0.38 (0.15, 0.97) | 0.41 (0.14, 1.24) |
| HR (95% CI)^b^ | 0.97 (0.77, 1.22) | Ref | 0.88 (0.64,1.22) | Ref |
|  | <65 years | | | |
| Number of patients | 8,387,264 | 9,195,832 | 3,706,622 | 3,706,622 |
| Number of events | 740 | 670 | 295 | 300 |
| Mean follow-up days (SD) | 87.05 (12.73) | 87.22 (12.45) | 87.72 (11.18) | 87.53 (11.66) |
| Incidence rate (95% CI)^a,b^ | 0.32 (0.18, 0.55) | 0.30 (0.28, 0.33) | 0.32 (0.22, 0.46) | 0.33 (0.26, 0.42) |
| HR (95% CI)^b^ | 1.04 (0.59, 1.83) | Ref | 0.98 (0.84, 1.16) | Ref |
|  |  | | | |
|  | Male | | | |
| Number of patients | 3,976,719 | 4,639,431 | 1,883,976 | 1,883,976 |
| Number of events | 573 | 467 | 220 | 234 |
| Mean follow-up days (SD) | 87.11 (12.62) | 87.37 (12.11) | 87.88 (10.83) | 87.71 (11.28) |
| Incidence rate (95% CI)^a,b^ | 0.48 (0.22, 1.07) | 0.41 (0.31, 0.54) | 0.46 (0.24, 0.88) | 0.50 (0.28, 0.90) |
| HR (95% CI)^b^ | 1.19 (0.70, 2.02) | Ref | 0.94 (0.78, 1.13) | Ref |
|  | Female | | | |
| Number of patients | 6,160,749 | 5,564,363 | 2,576,461 | 2,576,461 |
| Number of events | 417 | 317 | 152 | 160 |
| Mean follow-up days (SD) | 87.05 (12.72) | 87.18 (12.52) | 87.59 (11.53) | 87.42 (11.97) |
| Incidence rate (95% CI)^a,b^ | 0.24 (0.14, 0.43) | 0.24 (0.21, 0.27) | 0.24 (0.17, 0.33) | 0.26 (0.22, 0.31) |
| HR (95% CI)^b^ | 0.998 (0.51,1.96) | Ref | 0.93 (0.69, 1.25) | Ref |
|  |  | | | |
|  | With diabetes | | | |
| Number of patients | 1,181,931 | 862,064 | 513,445 | 513,445 |
| Number of events | 165 | 124 | 64 | 79 |
| Mean follow-up days (SD) | 86.90 (13.04) | 87.27 (12.36) | 87.36 (12.11) | 87.25 (12.40) |
| Incidence rate (95% CI)^a,b^ | 0.50 (0.28, 0.91) | 0.58 (0.40, 0.84) | 0.53 (0.41, 0.68) | 0.61 (0.37, 1.01) |
| HR (95% CI)^b^ | 0.91 (0.72, 1.15) | Ref | 0.81 (0.58, 1.13) | Ref |
|  | Without diabetes | | | |
| Number of patients | 8,955,537 | 9,341,730 | 3,941,075 | 3,941,075 |
| Number of events | 825 | 660 | 312 | 315 |
| Mean follow-up days (SD) | 87.10 (12.63) | 87.27 (12.33) | 87.76 (11.12) | 87.57 (11.60) |
| Incidence rate (95% CI)^a,b^ | 0.32 (0.16, 0.64) | 0.29 (0.27, 0.32) | 0.32 (0.20, 0.50) | 0.33 (0.28, 0.40) |
| HR (95% CI)^b^ | 1.08 (0.58, 2.03) | Ref | 0.97 (0.72, 1.30) | Ref |
|  |  | | | |
|  | With ophthalmic conditions^c^ | | | |
| Number of patients | 1,902,626 | 1,728,537 | 939,680 | 939,680 |
| Number of events | 445 | 305 | 160 | 170 |
| Mean follow-up days (SD) | 87.48 (11.76) | 87.97 (10.61) | 88.04 (10.41) | 87.96 (10.65) |
| Incidence rate (95% CI)^a,b^ | 0.74 (0.25, 2.22) | 0.72 (0.40, 1.32) | 0.69 (0.37, 1.28) | 0.73 (0.38, 1.40) |
| HR (95% CI)^b^ | 1.04 (0.64, 1.70) | Ref | 0.94 (0.76, 1.17) | Ref |
|  | Without ophthalmic conditions^c^ | | | |
| Number of patients | 8,234,842 | 8,475,257 | 3,531,644 | 3,531,644 |
| Number of events | 545 | 479 | 211 | 215 |
| Mean follow-up days (SD) | 86.98 (12.89) | 87.12 (12.66) | 87.62 (11.46) | 87.42 (11.98) |
| Incidence rate (95% CI)^a,b^ | 0.23 (0.13, 0.42) | 0.24 (0.22, 0.26) | 0.23 (0.14, 0.39) | 0.25 (0.22, 0.29) |
| HR (95% CI)^b^ | 1.01 (0.60, 1.69) | Ref | 0.92 (0.55, 1.55) | Ref |

CI, confidence intervals; HR, hazard ratios; SD, standard deviation.

^a^Per 1,000 person-years.

^b^Data were pooled across databases using random-effects meta-analysis.

^c^Ophthalmic conditions included ophthalmic comorbidities and ophthalmic medication use.

**Table S20.** Subgroup analysis of number of patients and events, follow-up duration, incidence rate, and HR of rhegmatogenous retinal detachment comparing fluoroquinolones to extended-spectrum cephalosporins before and after propensity score matching across databases, by patient characteristic

|  | Before matching | | After matching | |
| --- | --- | --- | --- | --- |
|  | Fluoroquinolones | Extended-spectrum cephalosporins | Fluoroquinolones | Extended-spectrum cephalosporins |
|  | ≥65 years | | | |
| Number of patients | 1,750,204 | 448,083 | 445,827 | 445,827 |
| Number of events | 250 | 47 | 51 | 47 |
| Mean follow-up days (SD) | 87.22 (12.40) | 87.64 (11.56) | 87.72 (11.32) | 87.64 (11.56) |
| Incidence rate (95% CI)^a,b^ | 0.42 (0.12, 1.42) | 0.41 (0.15, 1.10) | 0.43 (0.13, 1.38) | 0.41 (0.15, 1.10) |
| HR (95% CI)^b^ | 1.10 (0.80, 1.51) | Ref | 1.08 (0.73, 1.61) | Ref |
|  | <65 years | | | |
| Number of patients | 8,387,264 | 3,382,687 | 3,048,070 | 3,048,070 |
| Number of events | 740 | 256 | 258 | 243 |
| Mean follow-up days (SD) | 87.05 (12.73) | 87.41 (12.01) | 87.56 (11.59) | 87.50 (11.80) |
| Incidence rate (95% CI)^a,b^ | 0.32 (0.18, 0.55) | 0.31 (0.26, 0.38) | 0.33 (0.19, 0.56) | 0.32 (0.23, 0.45) |
| HR (95% CI)^b^ | 1.04 (0.72, 1.49) | Ref | 1.06 (0.89, 1.27) | Ref |
|  |  | | | |
|  | Male | | | |
| Number of patients | 3,976,719 | 1,499,457 | 1,400,510 | 1,400,510 |
| Number of events | 573 | 161 | 160 | 153 |
| Mean follow-up days (SD) | 87.11 (12.62) | 87.43 (12.00) | 87.55 (11.66) | 87.50 (11.84) |
| Incidence rate (95% CI)^a,b^ | 0.48 (0.22, 1.07) | 0.44 (0.32, 0.60) | 0.43 (0.22, 0.87) | 0.44 (0.29, 0.65) |
| HR (95% CI)^b^ | 1.13 (0.69, 1.84) | Ref | 1.03 (0.77, 1.36) | Ref |
|  | Female | | | |
| Number of patients | 6,160,749 | 2,331,313 | 2,101,436 | 2,101,436 |
| Number of events | 417 | 142 | 153 | 134 |
| Mean follow-up days (SD) | 87.05 (12.72) | 87.44 (11.93) | 87.58 (11.53) | 87.53 (11.72) |
| Incidence rate (95% CI)^a,b^ | 0.24 (0.14, 0.43) | 0.25 (0.19, 0.33) | 0.28 (0.15, 0.54) | 0.26 (0.18, 0.38) |
| HR (95% CI)^b^ | 1.00 (0.73, 1.37) | Ref | 1.14 (0.91, 1.44) | Ref |
|  |  | | | |
|  | With diabetes | | | |
| Number of patients | 1,181,931 | 333,815 | 328,534 | 328,534 |
| Number of events | 165 | 37 | 42 | 36 |
| Mean follow-up days (SD) | 86.90 (13.04) | 87.32 (12.24) | 87.43 (11.97) | 87.34 (12.22) |
| Incidence rate (95% CI)^a,b^ | 0.50 (0.28, 0.91) | 0.47 (0.32, 0.68) | 0.52 (0.32, 0.84) | 0.46 (0.32, 0.66) |
| HR (95% CI)^b^ | 1.16 (0.81, 1.66) | Ref | 1.16 (0.75,1.82) | Ref |
|  | Without diabetes | | | |
| Number of patients | 8,955,537 | 3,496,955 | 3,172,792 | 3,172,792 |
| Number of events | 825 | 266 | 274 | 256 |
| Mean follow-up days (SD) | 87.10 (12.63) | 87.44 (11.93) | 87.59 (11.54) | 87.53 (11.73) |
| Incidence rate (95% CI)^a,b^ | 0.32 (0.16, 0.64) | 0.31 (0.24, 0.40) | 0.32 (0.14, 0.72) | 0.33 (0.23, 0.47) |
| HR (95% CI)^b^ | 1.04 (0.69, 1.58) | Ref | 1.00 (0.65,1.53) | Ref |
|  |  | | | |
|  | With ophthalmic conditions^c^ | | | |
| Number of patients | 1,902,626 | 673,842 | 645,047 | 645,047 |
| Number of events | 445 | 118 | 125 | 116 |
| Mean follow-up days (SD) | 87.48 (11.76) | 88.10 (10.31) | 88.18 (10.03) | 88.12 (10.25) |
| Incidence rate (95% CI)^a,b^ | 0.74 (0.25, 2.22) | 0.73 (0.37, 1.43) | 0.77 (0.28, 2.08) | 0.74 (0.36, 1.51) |
| HR (95% CI)^b^ | 1.05 (0.70, 1.58) | Ref | 1.07 (0.80, 1.42) | Ref |
|  | Without ophthalmic conditions^c^ | | | |
| Number of patients | 8,234,842 | 3,156,928 | 2,857,427 | 2,857,427 |
| Number of events | 545 | 185 | 148 | 173 |
| Mean follow-up days (SD) | 86.98 (12.89) | 87.29 (12.29) | 87.43 (11.92) | 87.37 (12.10) |
| Incidence rate (95% CI)^a,b^ | 0.23 (0.13, 0.42) | 0.24 (0.18, 0.32) | 0.20 (0.13, 0.31) | 0.24 (0.16, 0.38) |
| HR (95% CI)^b^ | 1.03 (0.80, 1.34) | Ref | 0.86 (0.69, 1.07) | Ref |

CI, confidence intervals; HR, hazard ratios; SD, standard deviation.

^a^Per 1,000 person-years.

^b^Data were pooled across databases using random-effects meta-analysis.

^c^Ophthalmic conditions included ophthalmic comorbidities and ophthalmic medication use.

**Table S21.** Subgroup analysis of number of patients and events, incidence rate, and HR of rhegmatogenous retinal detachment comparing fluoroquinolones to amoxicillin/clavulanate or ampicillin/sulbactam before and after propensity score matching across databases, by fluoroquinolone

|  | Before matching | | After matching | |
| --- | --- | --- | --- | --- |
|  | Fluoroquinolones | Amoxicillin/clavulanate or ampicillin/sulbactam | Fluoroquinolones | Amoxicillin/clavulanate or ampicillin/sulbactam |
|  | Respiratory fluoroquinolones | | | |
| Number of patients | 3,012,939 | 10,203,794 | 1,639,645 | 1,639,645 |
| Number of events | 387 | 784 | 161 | 167 |
| Mean follow-up days (SD) | 86.43 (14.14) | 87.27 (12.34) | 86.99 (13.01) | 86.87 (13.26) |
| Incidence rate (95% CI)^a,b^ | 0.46 (0.27, 0.78) | 0.32 (0.29, 0.35) | 0.40 (0.31, 0.53) | 0.42 (0.33, 0.53) |
| HR (95% CI)^b^ | 1.44 (0.93, 2.24) | Ref | 0.92 (0.75, 1.12) | Ref |
|  | Levofloxacin | | | |
| Number of patients | 2,605,568 | 10,203,794 | 1,390,552 | 1,390,552 |
| Number of events | 306 | 784 | 125 | 130 |
| Mean follow-up days (SD) | 86.30 (14.40) | 87.27 (12.34) | 86.82 (13.37) | 86.71 (13.58) |
| Incidence rate (95% CI)^a,b^ | 0.41 (0.22, 0.75) | 0.32 (0.29, 0.35) | 0.37 (0.27, 0.49) | 0.35 (0.21, 0.60) |
| HR (95% CI)^b^ | 1.28 (0.78, 2.12) | Ref | 0.96 (0.75, 1.23) | Ref |
|  | Moxifloxacin | | | |
| Number of patients | 371,344 | 10,203,794 | 231,465 | 231,465 |
| Number of events | 77 | 784 | 29 | 23 |
| Mean follow-up days (SD) | 87.17 (12.62) | 87.27 (12.34) | 87.63 (11.60) | 87.38 (12.18) |
| Incidence rate (95% CI)^a,b^ | 0.75 (0.39, 1.44) | 0.32 (0.29, 0.35) | 0.52 (0.36, 0.75) | 0.42 (0.28, 0.63) |
| HR (95% CI)^b^ | 2.39 (1.38, 4.14) | Ref | 1.26 (0.73, 2.17) | Ref |
|  | Gemifloxacin^c^ | | | |
| Number of patients | 28,065 | 3,739,941 | 28,058 | 28,058 |
| Number of events | NA^d^ | 277 | NA^d^ | NA^d^ |
| Mean follow-up days (SD) | 89.15 (7.08) | 89.74 (3.99) | 89.15 (7.08) | 89.13 (7.24) |
| Incidence rate (95% CI)^a^ | 0.44 (0.14, 1.36) | 0.30 (0.27, 0.34) | 0.44 (0.14, 1.36) | 0.44 (0.14, 1.36) |
| HR (95% CI) | 1.46 (0.47, 4.54) | Ref | 1.00 (0.20, 4.955) | Ref |
|  |  |  |  |  |
|  | Non-respiratory fluoroquinolones | | | |
| Number of patients | 7,124,529 | 10,203,794 | 3,075,884 | 3,075,884 |
| Number of events | 603 | 784 | 233 | 292 |
| Mean follow-up days (SD) | 87.35 (12.01) | 87.27 (12.34) | 88.07 (10.25) | 87.87 (10.83) |
| Incidence rate (95% CI)^a,b^ | 0.30 (0.15, 0.59) | 0.32 (0.29, 0.35) | 0.31 (0.16, 0.57) | 0.39 (0.28, 0.54) |
| HR (95% CI)^b^ | 0.94 (0.53, 1.66) | Ref | 0.80 (0.55, 1.17) | Ref |
|  | Ofloxacin^e^ | | | |
| Number of patients | 1,246,156 | 3,739,941 | 1,007,222 | 1,007,222 |
| Number of events | 71 | 277 | 59 | 55 |
| Mean follow-up days (SD) | 89.92 (2.19) | 89.74 (3.99) | 89.92 (2.21) | 89.90 (2.52) |
| Incidence rate (95% CI)^a^ | 0.23 (0.18, 0.29) | 0.30 (0.27, 0.34) | 0.24 (0.18, 0.31) | 0.22 (0.17, 0.29) |
| HR (95% CI) | 0.77 (0.59, 1.00) | Ref | 1.07 (0.74, 1.55) | Ref |
|  | Ciprofloxacin | | | |
| Number of patients | 5,605,717 | 10,203,794 | 2,049,832 | 2,049,832 |
| Number of events | 518 | 784 | 174 | 178 |
| Mean follow-up days (SD) | 86.66 (13.53) | 87.27 (12.34) | 87.15 (12.51) | 86.86 (13.22) |
| Incidence rate (95% CI)^a,b^ | 0.27 (0.11, 0.66) | 0.32 (0.29, 0.35) | 0.29 (0.13, 0.63) | 0.37 (0.31, 0.42) |
| HR (95% CI)^b^ | 0.86 (0.39, 1.89) | Ref | 0.83 (0.45, 1.54) | Ref |
|  | Norfloxacin^e^ | | | |
| Number of patients | 262,896 | 3,739,941 | 192,965 | 192,965 |
| Number of events | 13 | 277 | 10 | 11 |
| Mean follow-up days (SD) | 89.83 (3.23) | 89.74 (3.99) | 89.80 (3.54) | 89.74 (3.93) |
| Incidence rate (95% CI)^a^ | 0.20 (0.12, 0.35) | 0.30 (0.27, 0.34) | 0.21 (0.11, 0.39) | 0.23 (0.13, 0.42) |
| HR (95% CI) | 0.67 (0.38,1.17) | Ref | 0.91 (0.39, 2.14) | Ref |

CI, confidence intervals; HR, hazard ratios; NA, not applicable; SD, standard deviation.

^a^Per 1,000 person-years.

^b^Data are pooled across databases using random-effects meta-analysis.

^c^Only Taiwanese data contributed to the analysis given no cases receiving amoxicillin/clavulanate or ampicillin/sulbactam in the U.S. data.

^d^Data are not presented because of a cell number ≤3 from Taiwanese data, which follows the data protection policy in Taiwan.

^e^Only Taiwanese data contributed to the analysis given few patients received ofloxacin and norfloxacin in the U.S. data.

**Table S22.** Subgroup analysis of number of patients and events, follow-up duration, incidence rate, and HR of rhegmatogenous retinal detachment comparing fluoroquinolones to extended-spectrum cephalosporins before and after propensity score matching across databases, by fluoroquinolone

|  | Before PS matching | | After PS matching | |
| --- | --- | --- | --- | --- |
|  | Fluoroquinolones | Extended-spectrum cephalosporins | Fluoroquinolones | Extended-spectrum cephalosporins |
|  | Respiratory fluoroquinolones | | | |
| Number of patients | 3,012,939 | 3,830,770 | 2,095,203 | 2,095,203 |
| Number of events | 387 | 303 | 264 | 216 |
| Mean follow-up days (SD) | 86.43 (14.14) | 87.43 (11.96) | 86.54 (13.94) | 86.48 (14.09) |
| Incidence rate (95% CI)^a,b^ | 0.46 (0.27, 0.78) | 0.32 (0.23, 0.43) | 0.49 (0.35, 0.68) | 0.42 (0.35, 0.51) |
| HR (95% CI)^b^ | 1.47 (1.16, 1.85) | Ref | 1.01 (0.90, 1.13) | Ref |
|  | Levofloxacin | | | |
| Number of patients | 2,605,568 | 3,830,770 | 1,951,027 | 1,951,027 |
| Number of events | 306 | 303 | 210 | 199 |
| Mean follow-up days (SD) | 86.30 (14.40) | 87.43 (11.96) | 86.45 (14.12) | 86.40 (14.24) |
| Incidence rate (95% CI)^a,b^ | 0.41 (0.22, 0.75) | 0.32 (0.23, 0.43) | 0.40 (0.25, 0.64) | 0.40 (0.30, 0.54) |
| HR (95% CI)^b^ | 1.31 (0.97, 1.76) | Ref | 1.06 (0.87, 1.28) | Ref |
|  | Moxifloxacin | | | |
| Number of patients | 371,344 | 3,830,770 | 341,151 | 341,151 |
| Number of events | 77 | 303 | 72 | NA^c^ |
| Mean follow-up days (SD) | 87.17 (12.62) | 87.43 (11.96) | 87.02 (12.96) | 86.74 (13.61) |
| Incidence rate (95% CI)^a,b^ | 0.75 (0.39, 1.44) | 0.32 (0.23, 0.43) | 0.85 (0.59, 1.22) | 0.35 (0.11, 1.06) |
| HR (95% CI)^b^ | 2.46 (1.78, 3.40) | Ref | 1.96 (1.14, 3.35) | Ref |
|  | Gemifloxacin | | | |
| Number of patients | 36,027 | 3,830,770 | 34,145 | 34,145 |
| Number of events | NA^c^ | 303 | NA^c^ | NA^c^ |
| Mean follow-up days (SD) | 88.70 (8.67) | 87.43 (11.96) | 88.67 (8.77) | 88.36 (9.82) |
| Incidence rate (95% CI)^a,b^ | 0.46 (0.17, 1.24) | 0.32 (0.23, 0.43) | 0.48 (0.18, 1.30) | 0.29 (0.07, 1.20) |
| HR (95% CI)^b^ | 1.55 (0.58, 4.16) | Ref | 1.93 (0.33, 11.16) | Ref |
|  |  | | | |
|  | Non-respiratory fluoroquinolones | | | |
| Number of patients | 7,124,529 | 3,830,770 | 2,724,263 | 2,724,263 |
| Number of events | 603 | 303 | 243 | 226 |
| Mean follow-up days (SD) | 87.35 (12.01) | 87.43 (11.96) | 88.06 (10.28) | 87.95 (10.67) |
| Incidence rate (95% CI)^a,b^ | 0.30 (0.15, 0.59) | 0.32 (0.23, 0.43) | 0.35 (0.16, 0.79) | 0.34 (0.23, 0.51) |
| HR (95% CI)^b^ | 0.95 (0.65, 1.38) | Ref | 1.03 (0.70, 1.53) | Ref |
|  | Ofloxacin^d^ | | | |
| Number of patients | 1,246,156 | 1,539,750 | 1,009,382 | 1,009,382 |
| Number of events | 71 | 104 | 59 | 71 |
| Mean follow-up days (SD) | 89.92 (2.19) | 89.79 (3.55) | 89.92 (2.22) | 89.90 (2.43) |
| Incidence rate (95% CI)^a^ | 0.23 (0.18, 0.29) | 0.27 (0.23, 0.33) | 0.24 (0.18, 0.31) | 0.29 (0.23, 0.36) |
| HR (95% CI) | 0.84 (0.62, 1.14) | Ref | 0.83 (0.59, 1.17) | Ref |
|  | Ciprofloxacin | | | |
| Number of patients | 5,605,717 | 3,830,770 | 1,867,729 | 1,867,729 |
| Number of events | 518 | 303 | 184 | 155 |
| Mean follow-up days (SD) | 86.66 (13.53) | 87.43 (11.96) | 87.19 (12.42) | 87.03 (12.89) |
| Incidence rate (95% CI)^a,b^ | 0.27 (0.11, 0.66) | 0.32 (0.23, 0.43) | 0.31 (0.10, 0.93) | 0.32 (0.19, 0.52) |
| HR (95% CI)^b^ | 0.87 (0.48, 1.56) | Ref | 1.01 (0.56, 1.85) | Ref |
|  | Norfloxacin^d^ | | | |
| Number of patients | 262,896 | 1,539,750 | 227,869 | 227,869 |
| Number of events | 13 | 104 | 11 | 14 |
| Mean follow-up days (SD) | 89.83 (3.23) | 89.79 (3.55) | 89.83 (3.26) | 89.81 (3.39) |
| Incidence rate (95% CI)^a^ | 0.20 (0.12, 0.35) | 0.27 (0.23, 0.33) | 0.20 (0.11, 0.35) | 0.25 (0.15, 0.42) |
| HR (95% CI) | 0.73 (0.41, 1.30) | Ref | 0.79 (0.36, 1.73) | Ref |

CI, confidence intervals; HR, hazard ratios; NA, not applicable; SD, standard deviation.

^a^Per 1,000 person-years.

^b^Data are pooled across databases using random-effects meta-analysis.

^c^Data are not presented because of a cell number ≤3 from Taiwanese data, which follows the data protection policy in Taiwan.

^d^Only Taiwanese data contributed to the analysis given few patients received ofloxacin and norfloxacin in the U.S. data.

**Table S23.** Association between fluoroquinolones versus comparison antibiotics and rhegmatogenous retinal detachment before and after propensity score matching in the Taiwan NHIRD, by treatment setting

|  | HR (95% CI) | |
| --- | --- | --- |
|  | Fluoroquinolones versus  amoxicillin/clavulanate or ampicillin/sulbactam | Fluoroquinolones versus  extended-spectrum cephalosporins |
| Any settings | 0.84 (0.66, 1.07) | 1.02 (0.77, 1.35) |
| Outpatient visits | 0.94 (0.71, 1.23) | 0.90 (0.66, 1.24) |
| Emergent department visits or during hospitalization | 0.74 (0.42, 1.32) | 0.76 (0.40, 1.46) |

CI, confidence intervals; HR, hazard ratios; NHIRD, National Health Insurance Research Database.

**Table S24.** Crude incidence rates of retinal detachment between fluoroquinolones users and comparison groups in available cohort studies and our study

| Study[Reference] | Country | Sample size  for each exposure group and in total | Mean age, years | Male % | Follow-up duration | Crude incidence rate  for each exposure group^a^ |
| --- | --- | --- | --- | --- | --- | --- |
| Pasternak et al. 2013[13] | Denmark | - Fluoroquinolones: n=748,792 - Non-fluoroquinolones: n=5,520,446   Total n=6,269,238 | 59 | 34 | 10 days | - Fluoroquinolones: 0.25 - Non-fluoroquinolones: 0.19 |
| Eftekhari et al. 2014[15] | U.K. | - Fluoroquinolones: n=247,073 - Beta-lactam antibiotics: n=3,303,641   Total n=3,550,714 | 41 | 56 | 365 days | - Fluoroquinolones: 0.09 - Beta-lactam antibiotics: 0.06 |
| Daneman et al. 2015[17] | Canada | - Fluoroquinolones: n=657,950 - Non-fluoroquinolones: n=1,086,410   Total n=1,744,360 | 65 | 49 | 30 days | - Fluoroquinolones: 0.3 - Non-fluoroquinolones: 0.2 |
| Our study | Taiwan and the U.S. | - Fluoroquinolones: n=10,137,468 - Amoxicillin/clavulanate or ampicillin/sulbactam: n=10,203,794 - Extended-spectrum cephalosporins: n=3,830,770   Total n= 24,172,032 | 46 | 42 | 90 days | - Fluoroquinolones: 0.34 - Amoxicillin/clavulanate or ampicillin/sulbactam: 0.32 - Extended-spectrum cephalosporins: 0.32 |

U.K., United Kingdom; U.S., United States.

^a^Per 1,000 person-years.

**Figure S1.** Summary of available studies examining the association between fluoroquinolones use and retinal detachment (rhegmatogenous type mainly)

CI, confidence interval; EHR, electronic medical records; THIN, The Health Improvement Network; NR, not reported; U.K., United Kingdom; U.S., United States.

^a^Case only studies included self-controlled case-series studies (Chui et al. 2014, Shin et al. 2018, and Londhe et al. 2022), case cross-over studies (Raguideau et al. 2016), and one sequence symmetry analysis (Baek et al. 2018).

^b^Based on random-sampled datasets

^c^Time intervals before outcome occurrence (case-only or nested case-control studies) or after fluoroquinolone exposure (cohort studies)

^d^A prescription termination date overlapping with the date of outcome occurrence

^e^The odds ratios (95% CI) were 4.50 (3.56-5.70) and 1.39 (1.23-1.57) for current use and any use of fluoroquinolones within 1 year before outcome occurrence, respectively.

^f^The odds ratios (95% CI) were 1.25 (0.95-1.64) and 1.18 (1.11-1.26) for current use and any use of fluoroquinolones within 1 year before outcome occurrence, respectively.

^g^The odds ratios (95% CI) were 0.75 (0.40-1.39) and 1.00 (0.81-1.24) for any use of fluoroquinolones within 10 days and within 1 year before outcome occurrence, respectively.

^h^The hazard ratios (95% CI) were 1.29 (0.53-3.13), 0.97 (0.46-2.05), 1.37 (0.80-2.35), and 1.27 (0.93-1.75) for any use of fluoroquinolones within 0-10 days, within 11-30 days, within 31-60 days, and within 61-180 days after cohort entry, respectively.

^i^The hazard ratios (95% CI) were 0.56 (0.08-4.14) and 0.89 (0.58-1.36) for any use of fluoroquinolones within 30 days and within 1 year after cohort entry, respectively.

^j^The relative risk was not reported but the absolute risk was available: 0.002% (fluoroquinolones), 0.002% (macrolides), and 0.001% (β-lactams) within 30 days after cohort entry; corresponding number was 0.03%, 0.02%, and 0.03% for each antibiotic, respectively, within 1 year after cohort entry.

**REFERENCES**

1. National Health Insurance Administration. National Health Insurance Annual Report 2022-2023 [Chinese]. <https://www.nhi.gov.tw/Content_List.aspx?n=9223A12B5B31CB37&topn=4864A82710DE35ED> Accessed on December 13, 2023.

2. Lin LY, Warren-Gash C, Smeeth L, Chen PC. Data resource profile: the National Health Insurance Research Database (NHIRD). Epidemiol Health. 2018:40:e2018062.

3. IBM Watson Health^TM^. IBM MarketScan Research Databases for life sciences researchers. <https://www.ibm.com/downloads/cas/0NKLE57Y#:~:text=The%20IBM%C2%AE%20MarketScan%C2%AE,risk%20assessments%20(HRAs)%2C%20hospital> Accessed on December 13, 2023.

4. Chui CS, Man KK, Cheng CL, Chan EW, Lau WC, Cheng VC, et al. An investigation of the potential association between retinal detachment and oral fluoroquinolones: a self-controlled case series study. J Antimicrob Chemother. 2014;69(9):2563-2567.

5. Raguideau F, Lemaitre M, Dray-Spira R, Zureik M. Association between oral fluoroquinolone use and retinal detachment. JAMA Ophthalmol. 2016;134(4):415-421.

6. Shin JY, Jeong S, Jeon HL, Byun S, Park KH, Jeong HE, et al. The risk profile of rhegmatogenous retinal detachment before and after using a fluoroquinolone: a 12 year nationwide self-controlled case series study. J Antimicrob Chemother. 2018;73(12):3442-3453.

7. Baek YH, Park SJ, Jeong S, Oh IS, Jeong HE, Park KH, et al. Signal detection between fluoroquinolone use and the risk of rhegmatogenous retinal detachment: sequence symmetry analysis using nationwide South Korean healthcare database between 2004 and 2015. Clin Drug Investig. 2018;38(12):1179-1188.

8. Londhe AA, Holy CE, Weaver J, Fonseca S, Villasis-Keever A, Fife D. Risk of retinal detachment and exposure to fluoroquinolones, common antibiotics, and febrile illness using a self-controlled case series study design: retrospective analyses of three large healthcare databases in the US. PLoS one. 2022;17(10):e0275796.

9. Etminan M, Forooghian F, Brophy JM, Bird ST, Maberley D. Oral fluoroquinolones and the risk of retinal detachment. JAMA. 2012;307(13):1414-1419.

10. Fife D, Zhu V, Voss E, Levy-Clarke G, Ryan P. Exposure to oral fluoroquinolones and the risk of retinal detachment: retrospective analyses of two large healthcare databases. Drug Saf. 2014;37(3):171-182.

11. Choi SY, Lim HA, Yim HW, Park YH. Administration of oral fluoroquinolone and the risk of rhegmatogenous retinal detachment: a nationwide population-based study in Korea. PLoS one. 2018;13(4):e0195563.

12. Taher MK, Crispo JAG, Fortin Y, Moog R, McNair D, Bjerre LM, et al. Systemic quinolones and risk of retinal detachment III: a nested case–control study using a US electronic health records database. Eur J Clin Pharmacol. 2022;78(6):1019-1028.

13. Pasternak B, Svanström H, Melbye M, Hviid A. Association between oral fluoroquinolone use and retinal detachment. JAMA. 2013;310(20):2184-2190.

14. Kuo SC, Chen YT, Lee YT, Fan NW, Chen SJ, Li SY, et al. Association between recent use of fluoroquinolones and rhegmatogenous retinal detachment: a population-based cohort study. Clin Infect Dis. 2014;58(2):197-203.

15. Eftekhari K, Ghodasra DH, Haynes K, Kempen JH, VanderBeek BL. Risk of retinal tear or detachment with oral fluoroquinolone use: a cohort study. Pharmacoepidemiol Drug Saf. 2014;23(7):745-752.

16. Kapoor KG, Hodge DO, Sauver JLS, Barkmeier AJ. Oral fluoroquinolones and the incidence of rhegmatogenous retinal detachment and symptomatic retinal breaks: a population-based study. Ophthalmology. 2014;121(6):1269-1273.

17. Daneman N, Lu H, Redelmeier DA. Fluoroquinolones and collagen associated severe adverse events: a longitudinal cohort study. BMJ Open. 2015;5(11):e010077.
